# A health digital twin framework for discrete event simulation based optimised critical care workflows

**DOI:** 10.1038/s41746-025-01738-4

**Published:** 2025-06-19

**Authors:** Gayan Dihantha Kuruppu Kuruppu Appuhamilage, Maqbool Hussain, Mohsin Zaman, Wajahat Ali Khan

**Affiliations:** 1 School of Computing, College of Science and Engineering, University of Derby, Kedleston Road, Derby, UK DE22 1GB,; 2 Northampton General Hospital NHS Trust, Cliftonville, Northampton, UK NN1 5BD,

**Keywords:** Computer science, Risk factors, Health care economics

## Abstract

Digital twins have been used in industries and is now gaining traction in healthcare, particularly in precision medicine. Discrete Event Simulation is a modelling methodology for simulating processes and workflows in healthcare. This paper presents a methodology that integrates these technologies to optimise critical care workflows based on real-time state changes, emphasising patient safety, operational efficiency, and sustainability. This study proposes a novel dual-layer architecture to monitor physical and conceptual entities in the Critical Care Unit. In the current scope, this study aims to establish a methodology using Azure cloud to track treatment workflows in real-time. The results indicated that by reviewing observation forms alone successfully tracked 72% of staff-performed tasks in real-time. This study underscores the potential of digital twins to transform precision care in critical care delivery by bridging the gap between actual and ideal clinical practices.

## Introduction

Healthcare is a high-risk profession^1^, continuously evolving to integrate new technologies that enhance precision medicine. However, studies show some worrying estimated numbers relating to the harm and death caused by medical errors. In the United States, this is estimated to be greater than 750,000 cases of severe harm or death from medical error every year^1^. In the European Union, World Health Organisation estimates that one in ten patients experiences harm^2^. In the United Kingdom, the national state of patient safety report 2024 estimated that annual cost of these errors alone is 14 billion pounds^2^. The reason is 40–50% of these errors are thought to be preventable^2^. The healthcare professionals strive to reduce harm to their patients primarily because of vocational responsibility if not the Hippocratic oaths. The key question, then, is how stakeholders should approach healthcare provision to better identify sources of harm and implement strategies to minimise them.

This study started by looking at what has been done to understand the problems. This approach to understanding harm comes from a combination of systems theory, complexity science and human factors and ergonomics. All three share a common holistic perspective that recognises the interconnectedness of things, yet when it comes to understanding causality, they differ and thus the solutions offered might be different. Why need all three as the sources of error not only lie in human factors and ergonomics^3^, but also systems and complexity science as this can contribute almost 40% of the harm that comes to people^3^.

Healthcare provision is an interplay of multiple professionals, from doctors, nurses, paramedics, pharmacists, physiotherapists, to porters, managers and discharge specialists. All have a role to play, yet the way they function within that system is vastly different. The Accident and Emergency (A&E) department is very different to the pathology lab, which in turn is vastly different to how an operation theatre works. So even though these people all work together they are frequently interacting in novel ways while working in parallel. The other difference is how they interpret task data. The working pattern of a nurse or pharmacist, for example, is heavily structured. They have prescribed tasks that must be carried out in a fixed format with well-defined time intervals and responses. On the other hand, doctors work on the other end of the spectrum, their approach is frequently unstructured, they are individualistic in their approach and only bring structure after a diagnosis is made.

Overall healthcare delivery requires multiple professionals or agents who interact in both linear and non-liner behaviour. These professionals frequently adopt activity to reflect changing knowledge or ground conditions. These behaviours are always sensitive to initial conditions and legacy activity is frequently reflected in how people work. There are feedback loops influencing future behaviour. It is often difficult to identify the boundaries of the system and it is important to acknowledge the role of distributed control, i.e., there is no way to organise the entire systems behaviour. So, healthcare is a complex adaptive system.

This study looked at where the harm comes from through the lens of human factors and ergonomics to map frequent state changes and interconnected linear and non-linear agents in a clinical setting. Based on the contributors-to-error proportions came from human cognition, work environment, culture, service design, and the vast gap between work as done as opposed to how its imagined^4^. To resolve this, existing literature proposes Discrete Event Simulation to optimise workflows. Whose characteristics such as real-time simulation of discrete events and state changes in agents are considered, the best option appears to be the application of digital twin technology. Unlike most legacy work, the focus is not a condition or disease, but the system that provides healthcare to improve patient safety. So, this study looked at these factors more closely about cognitive errors^5–7^ coming from the tendency of the human brain to look for patterns and seek a first fit approach to diagnosis and decision making. Clinicians tend to not question their first assumptions even when a case does not progress as anticipated. The uncertainty is frequently identified and disregarded when it comes to clinical management plans. A clinician frequently needs to make decisions with partial data, is heavily reliant on heuristics and tends to only question decisions if things go catastrophically wrong.

Discrete Event Simulation is a modelling methodology to simulate processes or workflows of complex systems using state changes of activities and assets^8^. Digital twin is a simulation methodology to mirror physical entities in real-time, predicting, and evaluating the behaviour^9^. It has its origins in the manufacturing domain, where it was initially applied within product lifecycle management systems^10^. The legacy work (aerospace^10^, energy^11^, maritime^12^) shows reliable performance and digital twins were well-suited not only to the manufacturing industry^13^ but also to other domains/industries as well. As their potential became evident, digital twins gained attention from other sectors, becoming a strategic technology for key business players^14^. Although digital twins are now being explored in diverse domains, including healthcare, achieving optimal benefits relies on how well the domain aligns with the core characteristics of digital twin models^15^. However, compared to other sectors, realising similar capabilities in healthcare is more complex. Clinical workflows at a hospital such as emergency care and critical care units have many interrelated time critical activities performing unstructured diagnosis by doctors and structured treatment by nurses. Discrete Event Simulation based on historical and synthetic timestamp data to simulate complex systems has proved useful in many contexts. But in many contexts the main limitation of Discrete Event Simulation is that it does not provide/support a real-time representation of the workflows. Digital twins have the capability to represent physical entities in real-time, but it cannot be used as it is to represent complex workflows.

From a domain aspect, digital twins in health revolutionised management and delivery, disease treatments and prevention, product design and development, and personalised medicine. Existing research suggests the applications such as resource utilisation and managing workflows using digital twins to revolutionise the cyclical care process and enhance patient care^9^. On the other hand, Discrete Event Simulation in healthcare is used for stochastic modelling to address departments in hospitals such as emergency care to assess the impact of care process, patient flows, resource usage, and operational level issues. The applications were designed to address time and efficiency related matrices using hybrid models^16^.

To the best of our knowledge there are fewer applications for digital twins in healthcare for workflow optimisation due to limitations in demonstrating discrete events of dynamic and complex systems such as hospital departments^17–22^. Existing literature utilised digital twin applications to simulate physical entities such as staff, patients, and others rather than conceptual events such as care process or workflow in a hospital department^23–25^. The inherent limitation for that is real-time gathering data about care process or workflows does not exist and complicated process in a clinical setting^20^. But data related to real-time state of physical entities can be obtained through electronic health records, job lists, and observation forms.

As mentioned earlier, stochastic modelling using discrete events has helped researchers to simulate care processes and workflows to simulate patient flow or resource allocation. This study gathered data about physical entities and timestamps of events in a clinical process from various sources such as clinical databases, biomedical devices, and others, and then simulated these clinical events using software such as AnyLogic or MATLAB Simulink. Even though Discrete Event Simulation has helped to simulate clinical processes it is limited with unavailability of critical data points and does not simulate real-time state of the clinical process. Some of the legacy work in digital twin is presented as follows.

Penverne et al. used a simulation-based digital twin for emergency medical communion centre operations to assess accessibility on organisational scenarios to enable flexible call distribution using discrete events. This study was implemented using Witness by Lanner which is a Discrete Event Simulation software and simulated using historical data from emergency medical communication centres in Pays-de-la-Loire region in France. This study improved service quality by 17% to 22%^17^.

Gorelova et al. conducted simulations of patient flow of two assisted reproduction clinics in Alicante and Madrid using Discrete Event Simulation. This study used MATLAB Simulink environment to simulate digital twins of patients, hormone biosensors device, and patient care plans entities using real and synthetic data collected from the infertility clinics. This proposed method allowed identification of bottlenecks and proposes optimal flows by modelling patient flow and patient health status resulting reduction of patient wait times, reduced number of patient departures, and decrease in staff workload. This study concluded to use real-time simulation of the patients and staff to develop precise digital twins of the infertility clinics as their future work^18^.

Zhong et al. investigated modelling an integrated care unit to support resource management using a hybrid approach combining agent-based simulation to model physical entities and Discrete Event Simulation for model care pathways. This study used AnyLogic to simulate critical care pathways based on the historical records. This approach leveraged personalised treatments based on trajectories and care services based on patient care needs. This study stated the lack of operational data which are not easy to record, visualise, or provide insights. Limited performance matrices at system level causes issues in validation of data and improving operational efficiency of the integrated care unit. The results show use of autonomous agents such as equipment to model physical aspects and rosters, schedule change disciplines, and handoff process can be used to model service parameters in the simulation. This study concluded to develop a digital twin of the integrated care unit with two-way communication between the physical and virtual entities in real-time for personalised prediction based on real patient data^19^.

Zheng et al. introduced Discrete Event Simulation modelling to study operating room workflows to investigate the impacts of changes in resource demand, staff level, and operating room policies. This study collected data from a tertiary public hospital in Beijing, China and used Any Logic to simulate operating room workflows. This study performed what-if analysis on demand accommodation and demand for anaesthetists and operating rooms^20^.

Hassanzadeh et al. proposed Discrete Event Simulation to model operating theatres to assess the configuration of surgery affects, the key performance indicators related to efficiency. This study used SimPy library to implement the simulation using administrative databases of a major Australian hospital. The model was validated using different scenarios and performance metrics were included in this study^22^.

Zhong et al. investigated adopting the Systems Engineering for Patient Safety (SEIPS) 2.0 to develop conceptual representations of critical care delivery processes in digital twin of integrated care units. The hybrid simulation model integrated discrete-time events and autonomous agents to capture interactions in the integrated care unit by calibrating electronic health record data from Mayo Clinic Rochester. The results show that the simulation model can be used as in-silico testbed to investigate clinical process and workflows in real-time^21^.

Based on domain perspective, in the last 5, years researchers have published simulations of workflows based on discrete events to simulate complex systems in healthcare (e.g., hospitals, clinics) in order to design new workflows, evaluate strategies, and predict changes by changing key variables in the workflow in clinical settings such as operating room^20^ and paediatric otolaryngology clinics^26^. The research of digital twins in healthcare was based on real-time or historical data collected using monitoring equipment and electronic health record systems to simulate the state of physical resources and processes such as intensive care unit^27^, emergency medical communication centres^17^, and hospital operations^28^. Few studies suggested to simulate real-time overview using discrete events and digital twins as their future work^18,19^. To the best of our knowledge, the existing research does not provide any evidence of a real-time overview of the workflow, processes, and outcomes in healthcare due to high variability, uncertainty, and dynamic socio-technical system where human interaction influence the processes and outcomes.

Balasubramanyam et al. surveyed the research question and objectives, concepts and benefits, principles and frameworks, application areas, and challenges and limitations of digital twins in personalised healthcare. This study identified four layers such as device layer, data layer, modelling layer, and application layer to support design principles such as data collection and integration, data modelling and simulation, real-time analysis and decision support, security and privacy, interoperability, scalability, and user centric design when implementing digital twin frameworks in healthcare. This study showed end to end digital twin platforms such as Eclipse, Unity, or Azure cloud to resolve time, costs, resources, standards, and scalability constraints involving cloud-based digital twins healthcare applications^23^.

Wang et al. explored real-time patient data sharing, storage, and processing of emergency health. This study compared cloud platforms such as Amazon Web Services, Microsoft Azure, and Google cloud based on zone availability, security, and cost factors to manage healthcare data using data transmission formats and storage file formats based on standards such as Health Level Seven Fast Healthcare Interoperability Resources (HL7 FHIR), and Digital Imaging and Communications in Medicine (DICOM). This study showed using Amazon Web Services cloud to implement emergency healthcare solutions due to real-time data processing and analytics integrated tools^24^.

Jameil et al. addressed the limitations involving real-time patient monitoring due to low latency data transmission and efficiency resource management. This study implemented low-cost, low-latency multimodal sensors to simulate various physiological parameters based on dynamic optimisation modelling using Python based Pyomo software and Azure cloud and verified using machine learning algorithms. This study shows Azure cloud helped horizontal scaling to support robustness with increasing patient and data volumes, effective digital twin modelling and integration using c-sharp and JavaScript Object Notation based Linked Data (JSON-LD) languages, and visual tools such as Azure Digital Twin Explorer to support visual exploration and administration^25^.

From a technology persepective, the literature review relies on existing studies such as AnyLogic^19,20^ or related proprietary software^17,18,22,27^ to simulate discrete events based on historical databases. To achieve real-time simulation, study conducted review literature based on proprietary, opensource, or custom cloud-based methodologies to explore capabilities such as establishing two-way communication^25^, scalable data storage and computation services^24^, and analytical capabilities in healthcare. The existing literature suggest to use cloud-based platforms, since Discrete Event Simulation software does not provide horizontal scaling which is essential to patient demand in a clinical setting. The current scope used Microsoft Azure cloud to implement the digital twin framework to identify stability, latency, and performance of the Azure services and the next phases will compare the performance with other proprietary platforms.

Overall, the literature review on both domain and technology perspectives of this study has identified the quality of the discrete event data, scope limitations, technical complexity, regulatory gaps, ethical concerns and cost all contributed to challenges faced by different projects. Also, the evaluation matrices for clinical workflows were limited due to difficulty of data collection of the states in a workflow^19^. To evaluate the framework, this study has identified the following criteria based on the methodology:

1. All interventions are time neutral.
2. Counterbalance with time reduction.
3. No added time spent on data collection.

The final approach was to bring this together by adoption of Discrete Event Simulation using Cynefin framework based on digital twin technology, i.e. can digital twin track real-time activities, identify which of the four domains the activity falls in, compare activity to ideal (compare work as done to work as imagined) in areas of best and good practice, and finally identify if stakeholders can if fixed, governing and enabling constraints existed and if so, so how effective were they.

The basic questions study has resolved in the methodology:

1. Can each activity be tracked in real-time in clinical workflow settings?
  This to be based on whether the activity can be classified as clear, complicated, complex or chaotic. In real life most of these overlap in the provision of care of any given patient in any clinical setting. From a technical perspective this study has resolved the complexities regarding data collection of physical states associated with critical care workflows. The relationship between entities such as patients, staff, and activities were tracked real-time using discrete events during the treatment process.
2. Can the framework identify areas of best and good practice?
  First, this study identified areas of good and best practice. This study then analysed documents from five NHS trusts in the UK. This study focused on looking at the Standard Operating Procedures (SOPs) and seeing if SOPs metthe criteria for ISO 31000 Risk Framework^4^. SOPs met the criteria were used to generate flow charts from more than half the documents, and that none of the documents identified hazards with each step of the SOPs or any risk mitigation strategy. The documents were focused on work as imagined and as such seemed to disregard work as done. So just building on existing documents was not an option.
  Last, this study decided to formulate process maps from scratch, identify fault points and build on this by mapping this against previous incidents. This study identified areas of good and best practice based on national standards and guidance. For some roles like nurses and nutritionists this was relatively easy, yet for others like physicians this was rather difficult, or in simple terms structured tasks usually had some evidence base or recommendations behind them whereas unstructured tasks did not.
3. Can stakeholders learn on past mistakes?
  This study trained doctors on incident analysis and reports from the incident reporting tool commonly used in the NHS (Datix incident & risk reporting). The findings, though somewhat concerning, were not entirely unexpected, system factors were poorly identified, including environment, or organisational culture, as contributors and as such the solution offered frequently was training. This frequent lack of relevant data meant that past data could not be used as it was not reliable to train any system or map against the flow charts.
4. Can stakeholders modify the behaviour?
  This is required for new doctors to the critical care unit department who are not accustomed to the culture and environment. Buy-in was sought from existing workforce and the way the intervention was structured was to introduce gamification into the clinical space. Some of the new starters were inducted into gamification, they were given avatars and tasks that gave them points or took them away based on behaviour. This was quite successful in confirming the anticipated outcomes.

## Results

The overall results are concluded from two perspectives: domain and technical perspectives. The goal of the domain perspective is to prove that the digital twin framework can calculate and measure state changes in the physical digital twin layer is used to synchronise the conceptual digital twin layer to track the tasks and activities in real-time. The goal of the technical perspective is to identify how cloud infrastructure helped to seamlessly connect both physical and digital entities in real time. The data does not contain any patient or staff specific information and is anonymised by randomly generated resource identities for personnel associated during the data collection due to ethical reasons.

### Domain perspective

**Fig. 1:**
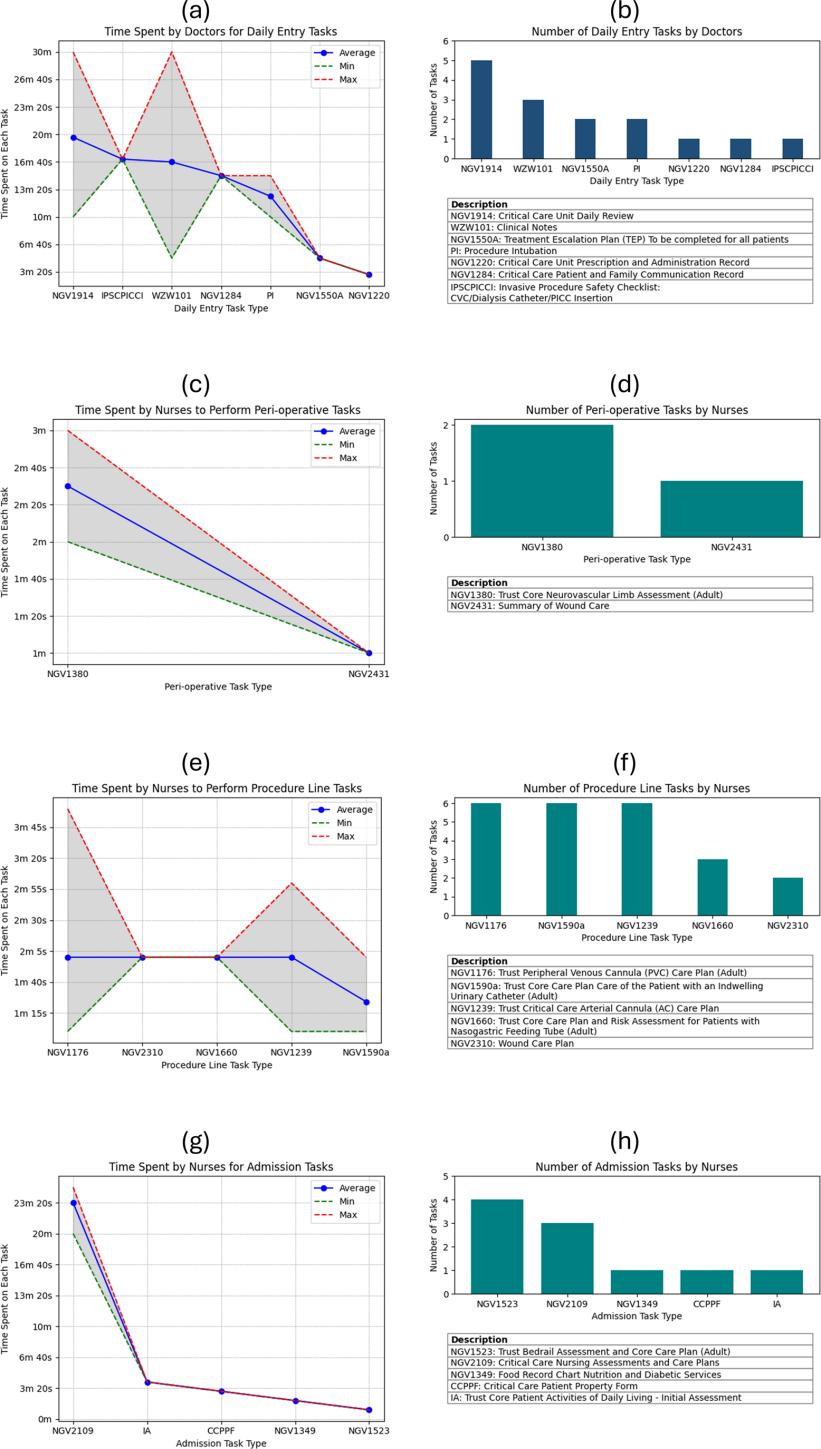
Domain perspective results. **a** The panel shows time spent in minutes by doctors to perform daily entry tasks. The line graph was created using data collected during the trial period. The discrete events associated with daily entry tasks by doctors were recorded in Supplementary Figs. 50–68 and summarized in Supplementary Table 1. The study sorted data in descending order, with the y-axis represents time spent on each task in minutes/seconds and the x-axis representing the task type. The red dotted line indicates the maximum time, the green dotted line indicates the minimum time, and the blue line represents the average time taken to perform each task during the data collection period. **b** The panel shows frequency of daily entry tasks. The bar graph was created using data collected during the trial period. The discrete events associated with daily entry tasks by doctors were recorded in Supplementary Figs. 50–68 and summarized in Supplementary Table 1. The study sorted data in descending order, with the y-axis representing the number of events and the x-axis representing the task type. The secondary table below the bar chart is a glossary table describing detailed names of each daily entry task type. **c** The panel shows time spent in minutes by nurses to perform peri-operative tasks. The line graph was created using data collected during the trial period. The discrete events associated with peri-operative tasks by nurses were recorded in Supplementary Figs. 1–6 and summarized in Supplementary Table 2. The study sorted data in descending order, with the y-axis representing the time spent on each task in minutes/seconds and the x-axis representing the task type. The red dotted line indicates the maximum time, the green dotted line indicates the minimum time, and the blue line represents the average time taken to perform each task during the data collection period. **d** The panel shows frequency of peri-operative tasks. The bar graph was created using data collected during the trial period. The discrete events associated with peri-operative tasks by nurses were recorded in Supplementary Figs. 1–6 and summarized in Supplementary Table 2. The study sorted data in descending order, with the y-axis representing the number of events and the x-axis representing the task type. The secondary table below the bar chart is a glossary table describing detailed names of each peri-operative task type. **e** The panel shows time spent in minutes by nurses to perform procedure line tasks. The line graph was created using data collected during the trial period. The discrete events associated with procedure line tasks by nurses were recorded in Supplementary Figs. 13–31 and summarized in Supplementary Table 4. The study sorted data in descending order, with the y-axis representing the time spent on task in minutes/seconds and the x-axis representing the task type. The red dotted line indicates the maximum time, the green dotted line indicates the minimum time, and the blue line represents the average time taken to perform each procedure line task during the data collection period. **f** The panel shows frequency of procedure line tasks. The bar graph was created using data collected during the trial period. The discrete events associated with procedure line tasks by nurses were recorded in Supplementary Figs. 13–31 and summarized in Supplementary Table 4. The study sorted data in descending order, with the y-axis representing the number of events and the x-axis representing the task type. The secondary table below the bar chart is a glossary table describing detailed names of each procedure line task type. **g** The panel shows time spent in minutes by nurses to perform admission tasks. The line graph was created using data collected during the trial period. The discrete events associated with admission tasks by nurses were recorded in Supplementary Figs. 32–40 and summarized in Supplementary Table 5. The study sorted data in descending order, with the y-axis representing the time spent on each task in minutes/seconds and the x-axis representing the task type. The red dotted line indicates the maximum time, the green dotted line indicates the minimum time, and the blue line represents the average time taken to perform each admission task during the data collection period. **h** The panel shows frequency of admission tasks. The bar graph was created using data collected during the trial period. The discrete events associated with admission tasks by nurses were recorded in Supplementary Figs. 32–40 and summarized in Supplementary Table 5. The study sorted data in descending order, with the y-axis representing the number of events and the x-axis representing the task type. The secondary table below the bar chart is a glossary table describing detailed names of each admission task type.

The experimental data was formulated from the Critical Care Unit (CCU) at Northampton General Hospital NHS Trust. This setup consisted of 14 staff members with 10 patients over 7 days. During this study, the framework was evaluated by using takt-time analysis with the data collected from doctors (*n* = 11), nurses (*n* = 3), and patients (*n* = 10). This study categorised forms (*n* = 86) out of all forms (*n* = 120) which is 72% of forms used in critical care treatments were taken into this study to gather data based on process groups (*n* = 7). The number of process groups allocated to doctors (*n* = 1) and nurses (*n* = 6). Also, out of 86 tasks, 22% recorded data during the data collection period. The average, minimum, maximum, and count functions applied for each process group are shown for doctors in Fig. 1a and b daily entries tasks; And for nurses in Fig. 1c and d peri-operative tasks, Fig. 1e and f procedure lines tasks, and Fig. 1g and h admission tasks. The medical diseases tasks (see Supplementary Table 4), death and dying tasks (see Supplementary Table 7), and daily entry tasks (see Supplementary Table 8) were not summarised into graphs due to no incidents were recorded by nurses during the data collection period.

First, the daily entries process group by doctors included 11 shifts. Out of 20 tasks, 35% were reported during the data collection period (see Supplementary Table 2). As shown in Fig. 1a, doctors spent more time with high deviation to perform each task. The “Critical Care Unit Daily Review (NGV1914)” task and “Clinical Notes (WZW101)” task recorded 53% and on average 18 minutes and 32 seconds to perform by doctors. The average time spent to perform daily entry tasks is 12 minutes and 41 seconds which is higher than nurses. Also as shown in Fig. 1b “Critical Care Unit Daily Review (NGV1914)” task recorded more frequently with 33% of all recorded data associated with daily entry tasks during the data collection period.

Second, the peri-operative process group by nurses included 2 shifts. Out of 6 tasks, 33% were reported during the data collection period (see Supplementary Table 3). As shown in Fig. 1c, nurses spent 1 minutes and 45 seconds on average to perform peri-operative tasks. Also as shown in Fig. 1d “Trust Core Neurovascular Limb Assessment (Adult) (NGV1380)” task recorded more frequently with 66% of all recorded data associated with peri-operative tasks during the data collection period.

Third, the procedure lines process group by nurses included data from 5 shifts. Out of 13 tasks, 38% were reported during the data collection period (see Supplementary Table 5). As shown in the Fig. 1e, nurses spent less time with more frequency in each task type. The “Trust Peripheral Venous Cannula (PVC) Care Plan (Adult) (NGV1176)” task, “Trust Core Care Plan Care of the Patient with an Indwelling Urinary Catheter (Adult) (NGV1590a)” task, and “Trust Critical Care Arterial Cannula (AC) Care Plan (NGV1239)” task recorded 78% and on average 2 minutes to perform by nurses. The nurses spent 1 minutes and 53 seconds on average to perform procedure line tasks. Also as shown in Fig. 1f “Trust Peripheral Venous Cannula (PVC) Care Plan (Adult) (NGV1176)” task, “Trust Core Care Plan Care of the Patient with an Indwelling Urinary Catheter (Adult) (NGV1590a)” task, and “Trust Critical Care Arterial Cannula (AC) Care Plan (NGV1239)” task recorded more frequently with 83% of all recorded data associated with procedure lines tasks during the data collection period.

Forth, the admission process group by nurses included data from 4 shifts. Out of 8 tasks, 75% were reported during the data collection period (see Supplementary Table 6). As shown in Fig. 1g, nurses spent 23 minutes and 20 seconds on “Critical Care Nursing Assessments and Care Plans (NGV2109)” task and 2 minutes and 30 seconds to complete other task types in the admission process group. Also as shown in Fig. 1h “Trust Bedrail Assessment and Core Care Plan (Adult) (NGV1523)” task recorded more frequently with 40% of all recorded data associated with admission tasks during the data collection period.

From domain perspective, data collection was integrated as an additional task within existing workflow. This has led staff members to spend less time to perform tasks such as the Trust Bedrail Assessment and Core Care Plan (Adult) (NGV1523 07/18) in the admission process group even though this task typically requires more time due to the busy nature of shift hours. In practice, these tasks often took more than one minute to complete. It was observed that staff members would log the start and end times for these tasks at their initiation rather than upon completion which did not reflect the actual time taken. Also, the doctors took 12 to 18 minutes to complete daily entry process group, with significant high or low deviations on certain task types. In contrast, nurses spent 2 to 4 minutes on peri-operative, procedure lines task types, and certain admission tasks with less variation. This difference is attributed to doctors primarily performing unstructured tasks, whereas nurses engage in more structured tasks.

### Technology perspective

The data was taken out of Azure analytics associated with Azure IoT Hub and Azure Digital Twin instances. The data collection period of this study was carried out for one week using barcode readers to simulate Internet of Things interfaces and online dashboard to simulate observation forms. The data consists of telemetry events, trigger events, routing latency, and number of digital twins. A telemetry event is a unit of interaction triggered by the edge devices. Each telemetry event holds a payload of information regarding the state of the physical system. In this study the payload consists of discrete time intervals of the starting and end timestamps of the activity and masked identification data about the patient, staff, and observation form. The trigger events communicate event data based on notification types associated with the internal state of the service. For example, trigger events can be a telemetry event associated with Azure IoT Hub service or digital twin update event associated with Azure Digital Twins service. The routing latency is the amount of time to consumed by the service to deliver the event to downstream application which is measured in milliseconds. A digital twin is an instance of a physical entity stored in a digital twin instance. From the technology perspective, this study aimed at consistent and real-time connectivity between physical and digital worlds, transferring the state of the staff, patients, observation forms to the physical digital twin layer, data processing and simulation of interactions and workflows in the conceptual digital twin layer, and dynamic management of digital twins.

**Fig. 2:**
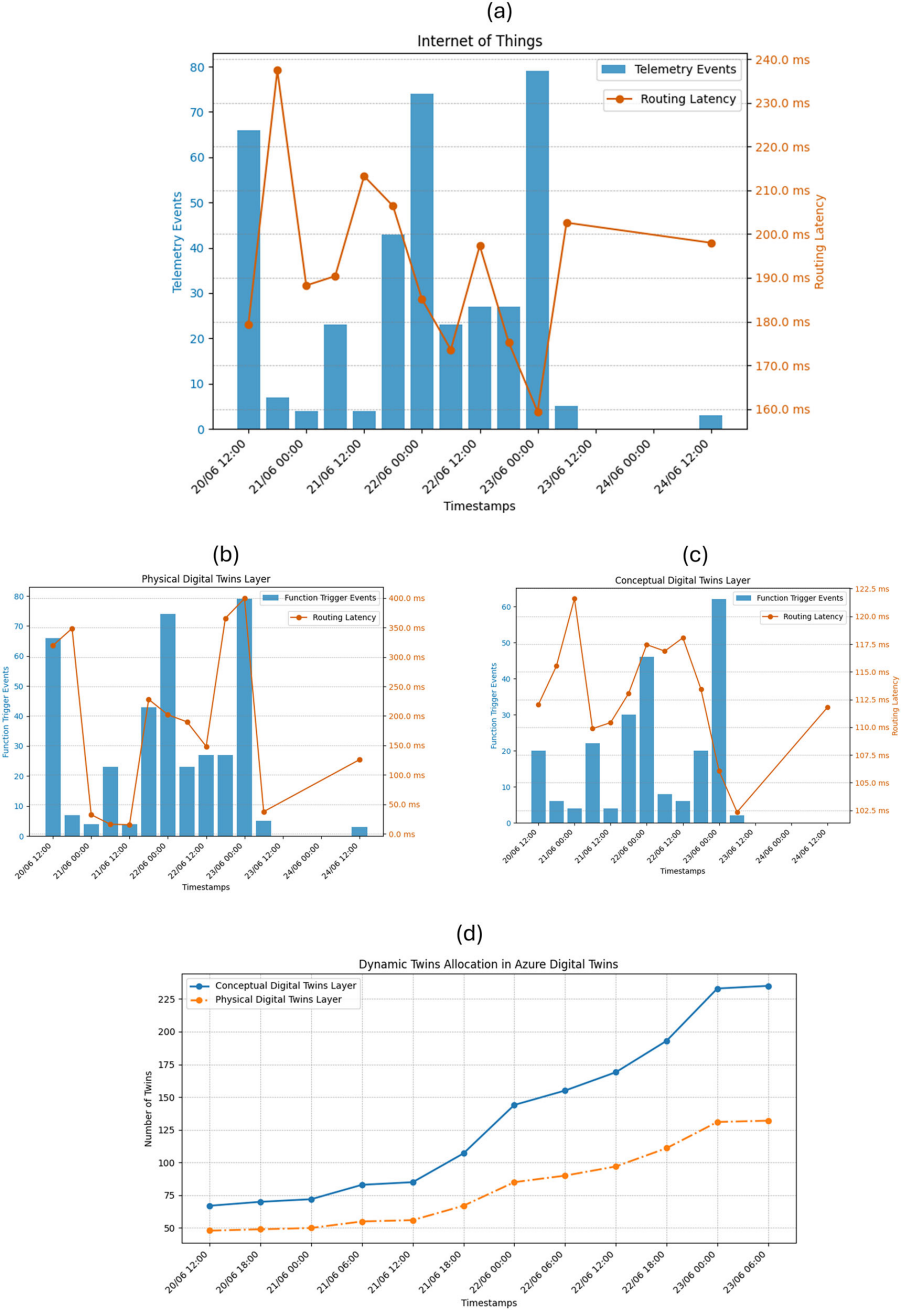
Technology perspective results. **a** The number of telemetry events and routing latency of the Azure Internet of Things service. The graph was created using data collected during the trial period. The Azure metrices related to number of telemetry events and routing latency were downloaded from Azure analytics and metrics service associated with Azure Internet of Things service. The data was taken from Supplementary Table 9: Internet of Things. Due to lack of activity the graph was created based on period between 20/06/2024 12:00 and 24/06/2024 12:00. The orange line shows average routing latency and blue bar chart shows total number of telemetry events of each time interval. **b** The number of telemetry events and routing latency of the Physical Digital Twin Layer. The graph was created using data collected during the trial period. The Azure metrices related to number of function trigger events and routing latency were downloaded from Azure analytics and metrics service associated with Azure Digital Twins service. The data was taken from Supplementary Table 10: Digital Twin Layers (Latency) and Supplementary Table 11: Digital Twin Layers (Triggers) associated with physical digital twin layer. Due to lack of activity the graph was created based on period between 20/06/2024 12:00 and 24/06/2024 12:00. The orange line shows average routing latency and blue bar chart shows total number of function events of each time interval. **c** The number of telemetry events and routing latency of the Conceptual Digital Twin Layer. The graph was created using data collected during the trial period. The Azure metrices related to number of function trigger events and routing latency were downloaded from Azure analytics and metrics service associated with Azure Digital Twins service. The data was taken from Supplementary Table 10: Digital Twin Layers (Latency) and Supplementary Table 11: Digital Twin Layers (Triggers) associated with conceptual digital twin layer. Due to lack of activity the graph was created based on period between 20/06/2024 12:00 and 24/06/2024 12:00. The orange line shows average routing latency and blue bar chart shows total number of function events of each time interval. **d** The number of digital twins allocated in physical and conceptual digital twin layers. The line graph was created using data collected during the trial period. The Azure metrices related to number of function trigger events and routing latency were downloaded from Azure analytics and metrics service associated with Azure Digital Twins service. The data was taken from Supplementary Table 12: Digital Twin Layers (Count) associated with both physical and conceptual digital twin layer. Due to lack of activity the graph was created based on period between 20/06/2024 12:00 and 23/06/2024 06:00. The orange line shows total number of physical twin instances created in the physical digital twin layer and blue line shows total number of conceptual twin instances created in conceptual digital twin layer during the trial period. This graph proves the dynamic digital twin allocation based on demand.

Figure 2a shows number of telemetry events and routing latency in the Azure IoT Hub instance for a period from 2024/06/20 to 2024/06/24 (see Supplementary Table 9). The data provides consistent and real-time communication between the physical and digital worlds without any failures. During the data collection period many staff members actively registered events during night shift which is time between 20:30 and 08:30. The latency is between 240 and 160 milliseconds, averaging at 200 milliseconds. The number of telemetry events corresponds to the latencies, but factors such as the size of the telemetry payload and the time between two telemetry events were also could cause higher latencies. The takeout point from the Azure IoT Hub data is to reduce the payload size and better networking configuration for immediate updates.

Figure 2b, c shows number of trigger events and latency data for (b) physical digital twin instance and (c) conceptual digital twin instance for the period from 2024/06/20 to 2024/06/24 (see Supplementary Tables 10–11). The data provides consistent and real-time transferring of the state between physical digital twin layer to conceptual digital twin layer. As shown in Fig. 5 and Fig. 6c, the number of elementary events is equal due to each trigger event by the Azure IoT Hub sends event data downstream 100% to the physical digital twin layer without any dead-letter events or failures. The latency in the physical digital twin layer is much higher than the Azure IoT Hub or in the conceptual digital twin layer due to processing, validating, and mapping the workflows of the unit in the conceptual digital twin layer. The takeout point from Fig. 2b is the framework requires faster execution time to reduce the latency. Figure 6c shows number of trigger events and latencies when managing the conceptual digital twin layer. The downstream data from physical digital twin layer, already determined and updated the organisational layout and workflows in the conceptual digital twin layer. Both digital twin instances showed variability in latency, with both instances recording higher average latencies, possibly suggesting external factors such as load during the data collection period. Additionally, there are inconsistencies in the conceptual twin data due to users using the online dashboard to record tasks without using IoT devices.

During the earlier iterations this study observed a latency of 10 seconds when querying digital twin instances. This issue persisted as staff members often recorded both start and end events with a brief period, even though tasks generally took more than 10 seconds to be completed for any given scenario. This resulted in duplication of activities and tasks digital twins in the conceptual digital twins during each event. To address this latency, study implemented API calls using Azure SDK to retrieve digital twins as a default rather than relying on query. This made querying less feasible when the digital twins required constant read and write operations.

Additionally, this study used dynamically generated instances of digital twins for a period from 2024/06/20 to 2024/06/24 (see Supplementary Table 12). The digital twins were created or deleted based on the physical overview of the critical care unit. Figure 2d shows dynamic resource allocation based on the demand. For example, if a newly registered staff member started an activity with a new barcode reader, a set of ports, session, staff, activity, and task digital twins would be generated in both physical and conceptual digital twin instances. In such cases, naming digital twins presented unique problems since the naming conventions and Azure Event Grid tend to execute each event executed nanoseconds apart. So, any standard time-based identification was incompatible and caused unpredictability due to duplication of digital twins. Therefore, Globally Unique Identifiers (GUIDs) were suffixed to digital twin identifier to eliminate duplication. Also, GUIDs were used to track each telemetry event from the beginning to the end of the system by attaching them as identifiers to each IoT telemetry event which helped to identify and track telemetry events from Event Grid.

To increase security, stability, maintainability, and lower maintenance costs, this study used Microsoft .NET framework as default environment to develop all the software required by the framework as it more stable releases and documentation. This approach reduced conflicts when projects interact with each other and increased maintainability of the code base. By using Azure SDK for dotnet for client and management services helped to eliminate many external dependencies which enhanced the security and stability of the code. This approach contributed to the implementation phase of this project with less time and improved quality and control in mind.

In addition, standard or basic features of the Azure platform led to unique challenges. Such as cold start, the latency of function apps, which would sleep after inactivity and in some cases took at least 90 seconds to start. This caused duplication and errors in the conceptual twins as the digital twins were either not created or did not have updated properties. Instead of implementing queues in the IoT Hub client, the subscription plans were upgraded to premium versions and Event Grid trigger functions were integrated into a single app project to reduce costs and increase availability. This significantly eliminated data duplication in the conceptual twins.

Moreover, the barcode readers used Bluetooth to communicate with the hub application. Which limited the number of patients that could be covered in the critical care unit. The barriers and interferences, such as the thick wall dividing the east and west wards and glass dividers restricted the signal strength of each barcode reader to a maximum of 5 meters. To record data, the online dashboard and manual forms were used as an alternative to cover more patients and to supplement the use of barcode readers. During the production phase nurses and support staff adopted this hybrid method due to limited access to computers and barcode readers. The other limitation of the barcode readers is their inability to process data within the device itself. The hub application managed this limitation by queuing the inputs. In future phases, the hub application will be used to act as an interface to data sources such as electronic health records, medical equipment, observation forms, or custom applications.

Also, Azure uses a global naming schema for identifying its resources. For example, if a resource can establish connectivity with the outside, its name is typically permanent and cannot be changed in the future. Each name also has constraints, such as alphabetical or numerical limits and character length restrictions. Using full names to identify each resource can be complex and may require recreating the entire resource with a different name. To address this, this study employed a four-part naming strategy and used the NHS Digital Data Repository to create a standardised, uniquely readable identification system for naming these resources to overcome the limitations. First, to identify the project, this study used the “uod-nhs” as University of Derby – NHS to distinguish other ongoing projects. Secondly, to identify the hospital and unit, formatted as “rns-rns01-78h,” which includes region, hospital, and department identifiers as prefix. Thirdly, two or three words to identify the project name. Last, initials of the Azure service name, such as “sqldb” for SQL Database, “wa” for Web App, and “egt-fa” for Event Grid Trigger Function Apps, were used. This approach streamlined the naming process, ensuring each resource name was unique among globally deployed resources, and helped scale each resource without causing conflicts.

To sum up the technology perspective, this study noticed that Azure cloud-maintained connectivity with devices throughout the data collection period. Additionally, the Azure IoT Hub executed operations with latency below 1 second. This study’s main feature, integrating multiple layers, performed well with latency of less than 500 milliseconds. Azure Entra’s application scope helped establish secure, symmetric-key-based applications for each endpoint to manage resources with its built-in HTTP client. The built-in features of the Microsoft Azure SDK helped reduce reliance on external dependencies and facilitated upgrading the source code to more recent stable releases without conflicts. The Azure Resource Manager SDK provided a programmable interface to allocate resources in Azure Cloud, enabling dynamic management of resources. Moreover, the naming strategy reduced clashes between globally deployed resources and streamlined the implementation process.

## Discussion

From a domain perspective, no data was intentionally collected about individual patients or specific staff in the first phase. The primary goal was to focus on tracking and quantifying activity while minimising any potential influence on data collection and performance. This approach ensured that the observed behaviours were as close to natural as possible. The Standard Operating Procedures (SOPs) reviewed during this phase were found to poorly reflect the actual work being performed. A more effective methodology would have involved interviewing various staff members to gain insights into their roles and daily activities. This would have provided a more accurate picture of the work environment and processes. The process mapping for each activity proved valuable in revealing discrepancies between how care was intended to be delivered versus how it was delivered. For example, the analysis of nursing documentation revealed that nurses were required to complete 15 separate documents totalling over 80 pages within the first 24 hours of a patient’s admission. Despite the volume, only three documents contained redundant information. Analysing and eliminating these redundancies could save approximately 25 minutes per shift, translating to a cumulative reduction of five hours of nursing time per shift, per day. Similarly, more than half of the documentation performed by physiotherapists was found to duplicate nursing reviews. Additionally, the review of pharmacists’ roles highlighted that a significant portion of their time was spent retrieving data already gathered by other systems, resulting in redundant tasks with limited value. Another key insight was that during periods of high demand, staff often delayed documentation until after tasks were completed. This suggests that to track activities in real-time data collection must extend beyond documentation to include metrics like changes in patient physiology, position, and interventions provided. As this study moved into fault point analysis in the next phase, it became evident that incident investigations disproportionately attributed errors to individuals, with limited attention given to systemic and process-related factors that contribute to harmful events. The lack of system and process thinking in service design was apparent, and when incidents occurred, the absence of comprehensive data and process maps made it difficult to trace back and identify the root causes. The proposed framework was envisioned to incorporate a comparative analysis of activity against an ideal and highlight discrepancies, helping with a better understanding of root causes around patient safety events.

The healthcare domain, physical entities were not limited to tangible physical objects (e.g., blood pressure devices); they could also include live observations, workflows, patients, clinicians, or clinical guidelines. Moreover, a single physical entity may be linked to multiple other physical entities, each with its own representative digital twin model. For example, in this study, the barcode devices were associated with different roles (nurses, consultants, etc.), each with its own digital twin model to track activities within the critical care unit workflows. This has created a many-to-many relationship between healthcare physical entities and digital twins, complicating the design and implementation of digital twin models in this domain. To address these complexities, a novel layered approach for digital twin design was proposed. The physical digital twin layer represented twins associated with tangible objects and their complementary components. For instance, the physical department, barcode scanners, and sessions. The conceptual digital twin layer represented twins of key domain entities, such as roles (nurses, consultants), workflows, observations, and tasks (e.g., observation forms), which were crucial to fulfil the business requirements. This multi-layered approach offered two primary advantages: first, it reduced the complexity of domain modelling; second, it allowed for a more comprehensive realisation of digital twin capabilities, maximising their benefits in healthcare applications.

The current phase of the digital twin framework was implemented as an additional task within the existing workflow. This approach limited both the quantity and type of data the study could collect due to the demanding environment of the critical care unit. Our focus in this phase was primarily on capturing the start and end times of tasks, rather than the granular details of the tasks themselves, due to the complexities of recording data while administering life-saving treatments. It became clear that collecting data for a digital twin framework as an extra task for staff was not feasible, especially as some staff members were initially hesitant to use it. Nevertheless, the framework’s design is flexible enough to integrate various data sources, and in subsequent phases, data from Electronic Medical Records and other data sources will be incorporated.

From a platform perspective, the existing layered digital twin framework is platform agnostic by design. But the existing framework was implemented using Azure Cloud to reduce time spent on implementing the framework due to its extensive documentation and the trustworthy environment it provides to secure interactions. The key limitation, however, is the lack of in-house capability to replicate Azure services. The open-source frameworks such as Eclipse Ditto and Hono offer alternatives for implementing digital twins on-premises^29^. However, implementing an open-source framework in a high-risk industry such as healthcare conflicts with its liability risks. Also, open-source alternatives such as Amazon Web Services (AWS) and Google Cloud Platform (GCP) were considered due to their performance in Apache benchmarks^30^. However, due to the limited time and scope of the current phase, this study chose Azure Cloud to implement the digital twin framework. The critical care unit adapts to patient demand and staff availability, causing fluctuations in the need for barcode readers. To reflect these changes, this study integrated the daemon application with an HTTP API, allowing dynamic management of devices in the cloud based on demand. The endpoint used for managing devices—enabling creation, reading of symmetric keys, and deletion operations in Azure IoT Hub via the Device Provisioning Service—was secured to be accessible only by the daemon application using Azure Entra Scope. Additionally, requiring a symmetric key in the HTTP request headers further enhanced security, enabling real-time adjustments to the digital twins based on demand.

From an interdisciplinary perspective, this study has designed the digital twin to be easily usable in other industries where human resources and complex rules are an essential part of the delivery of services. Another group that would benefit from this approach is where predictive judgement is required for complex data with limited information for people to make decisions. These two unique characteristics of our digital twin design mean that it can, on one hand, improve efficiency and be easily deployable in complex environments like government departments, such as taxation, and similarly be useful for entities like small businesses to help with the visualisation and validation of their day-to-day operations. Other examples would include the insurance industry, where predictive judgement is an essential part of calculations. This is achievable because this study structured the information around known models of service design and business management, with the intention that experts do not need to face a sharp learning curve to introduce the technology. The next phase of this study is to build interfaces to allow people with limited knowledge of system design and processes to easily build a twin without direct expert input. This study’s motivation remains healthcare, a field that changes how things are done significantly and regularly, yet such an approach would potentially allow models to be used in most industries where even people with limited knowledge of digital twinning can apply the technology to meet their needs.

## Methods

**Fig. 3:**
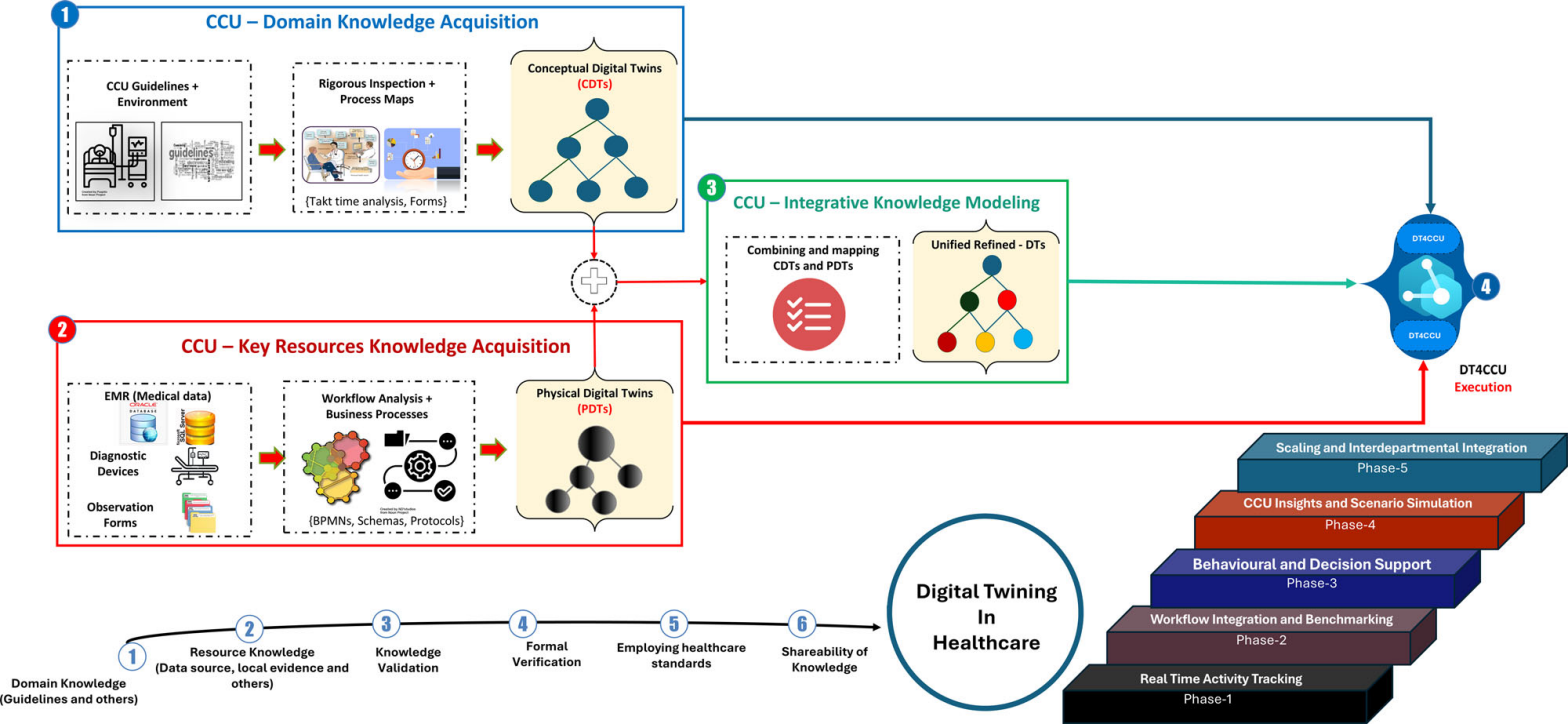
The four-step model of the framework and future work. **1**. Domain Knowledge Acquisition: The conceptual digital twins were created through a thorough inspection and process mapping of takt time analysis, critical care unit guidelines, and forms used to document the treatment process and workflows. **2**. Key Resources Knowledge Acquisition: The physical digital twins were developed by analysing workflows and business processes (e.g., BPMN, schemas, and protocols) using data sources from EMR systems, diagnostic devices, and observation forms. **3**. Integration of Physical and Conceptual Digital Twins: The physical and conceptual digital twins were combined into a unified framework that reflects all relationships and mappings between the components. **4**. Design Evaluation and Execution: The design is validated against artifacts in the target platform (Microsoft Azure in this case) to ensure robust implementation and execution environments. The future work of the framework is structured into the following phases. **1**. Phase 1 (Real-Time Activity Tracking): Can the proposed framework track activity live? **2**. Phase 2 (Workflow Integration and Benchmarking): Can it embed a data collection strategy into existing workflows? (with clinical and governance workflows as an example) and can we compare it against a standard? **3**. Phase 3 (Behavioural and Decision Support) : Can behavioural modification and decision support tools be introduced into practice? (with a focus on bias and noise around decision making). **4**. Phase 4 (CCU Insights and Scenario Simulation): Can the twin be used to implement an overall management strategy for a unit, by giving insights into system strengths and vulnerabilities and be used for scenario-based simulations? **5**. Phase 5 (Scaling and Interdepartmental Integration): Can the proposed framework scale for the integration of different departments of a hospital or Health system with their own digital twins. The figure contains official Azure architecture icons to communicate design and relationships between various Azure services used in this study. Microsoft permits the use of these icons in architectural diagrams. Source: https://learn.microsoft.com/en-us/azure/architecture/icons/.

At the current phase, real-time activity tracking employed a four-step process model systematically divided into four distinct phases. Each phase utilised the four-step process, as illustrated in Fig. 3. Throughout this process, the overall design aligned with the foundational criteria and requirements specific to the healthcare domain. For instance, domain knowledge was acquired through rigorous inspection of workflows, observation forms, protocols, guidelines, and consultations with healthcare professionals. The design underwent comprehensive validation through testing and baseline verification to ensure consistency. Additionally, the design incorporated criteria for compliance with healthcare standards (e.g., security and communication protocols like Health Level 7). Finally, the knowledge generated from the digital twin models can be shared across other organisations.

### Domain Knowledge Acquisition

**Fig. 4:**
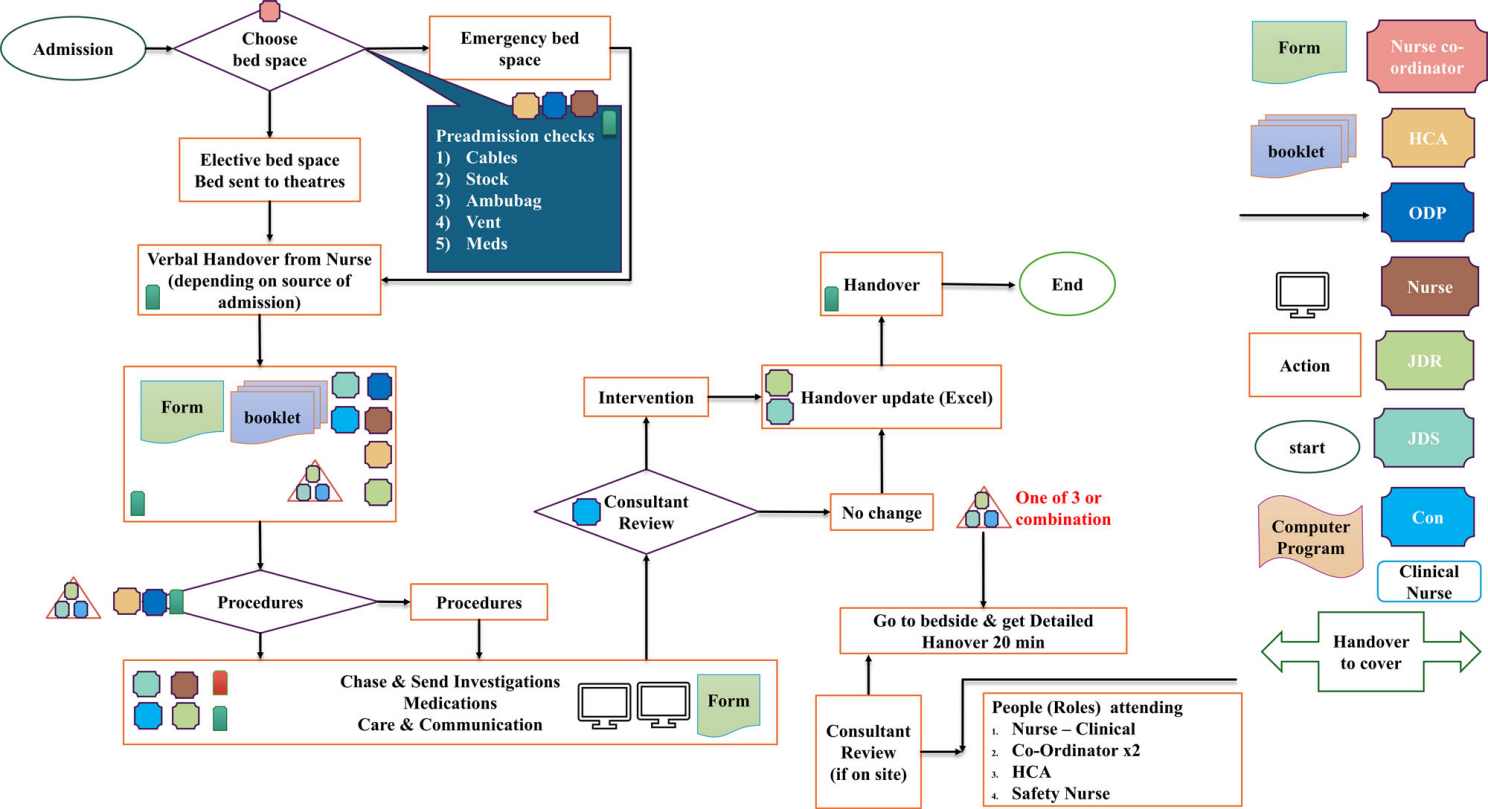
The process map of the patient admission. The process map is one of the many processes this study identified in the critical care unit. These were created for structured workflows to identify resource consumption and interactions with each resource. The process maps helped non-medical domain users to understand the workflow of tasks. The above process map involved admitting a patient to the critical care unit. This showed the type of bedspace required, preadmission checks, forms, procedures, and handover. This helped to identify the types of observation forms used to record patient data, staff roles interacting in each stage, and the workflow of the admission process which were used to categorise observation form groups.

Identifying process maps was a key part of creating the conceptual digital twin layer. During the design phase, this study used scanned observation forms (*n* = 116) to classify them according to the related processes carried out by nurses and doctors. These paper-based observation forms were empty and contained no patient-related or demographic data and were used to identify data points and the structure of the diagnosis and treatment processes. Due to the unstructured nature of tasks performed by doctors, this study sorted process maps (*n* = 1) using forms to record daily entries (*n* = 20). In contrast, the structured nature of tasks performed by nurses led to the creation of process maps (*n* = 6) using forms to document 1) daily entries (*n* = 22) 2) peri-operative tasks (*n* = 6) 3) medical diseases (*n* = 6) 4) procedure lines (*n* = 13) 5) admissions (*n* = 8) 6) death and dying processes (*n* = 10). This study had limited in time and scope, focusing on user roles (*n* = 2), with process maps created for doctors (*n* = 1) and nurses (*n* = 6). The Supplementary Table 1 shows classification of observation forms used in diagnosis and treatment tasks by doctors and nurses based on process groups. As shown in Fig. 4 the classification was then mapped into process maps to identify the rules, roles, processes, and standards of each workflow. Breaking down each task into a flowchart helped to communicate domain knowledge about tasks and activities in the critical care unit to technical experts. This study identified forms, tasks, subtasks, patients, staff, and units as conceptual digital twins within the digital twin framework.

### Key resources knowledge acquisition

This study observed non-patient medical data, diagnostic devices, and observation forms used in diagnosis and treatment processes to analyse the business and workflow of the critical care unit to build the physical digital twin layer. In this phase, alternatives such as web, mobile, and desktop apps were considered to interface physical twins with digital twins. Instead, this study employed a hybrid approach which utilised barcode readers as edge devices, paper sheets for recording manual entries, and a web-based interface to record discrete time events of tasks by the staff. The main reason for this approach was to provide alternative means for clinicians to interface with the digital twins without changing existing workflows. This study identified unit, hub, edge, and sessions as physical digital twins within the digital twin framework.

### Integration of physical and conceptual digital twins

This study identified the session digital twin to store information about the recorded discrete time event. The session twin was designed to store property values such as identifications of the patient, staff, observation form, start or end timestamps, and flag. If the flag is true, then the values in the property fields are matched with existing twins in the conceptual digital twin layer. For example, if a new staff member initiates a subtask on a task for an admitted patient, the session is used to create new staff, patient, task, and subtask twins. The task and subtask are used to record the start and end timestamps. Every time they interact with the patient, a new subtask twin is created under their task twin, recording their start and end timestamps. In the above example, if the staff member completes the subtask, they can set the flag to true and update the end timestamp property fields. If the staff member completes a task, the task twin will have the flag set to true, and the end timestamp property fields will be updated. When a staff member creates another subtask on the same task twin, a new task twin is created, and ongoing subtasks are displayed under the new task twin.

### Design, evaluation and execution

The Digital Twins Definition Language (DTDL) was used to describe twins. Each twin was encapsulated in a DTDL model, which is a JavaScript Object Notation (JSON) file that describes the properties and relationships. These models were then used to create digital twin instances in both physical and conceptual digital twin layers. The property attributes were used to store the states and changes of a particular digital twin using primary or complex data types. This study only used primary data types, as these did not store complex values such as patient treatment details. Each model could then be used to establish relationships with other models. Due to limitations in the Azure Digital Twins platform this study used DTDL v2 to describe these instances and used the Azure Software Development Kit which is a programmable interface to perform create, read, update, and delete operations for digital twin instances and their relationships.

**Fig. 5:**
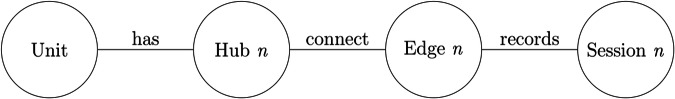
Physical Digital Twin Layer. The figure shows digital twin nodes and relationships in the physical digital twin layer. The figure describes conceptual entities such as unit, host, edge, and session twins. The unit twin is used to identify the organisation in the knowledge graph in the physical digital twin layer. This is denoted by the entity code of the critical care unit. This study did not use the abbreviations which distinguishes other wards such as the Critical Care Unit (CCU) or Coronary Care Unit (CCU) for staff. Additionally, the name length is limited to 1 KB and each name has to be unique. For this reason, the unit is named based on NHS guidelines as 78H rather than CCU or Critical Care Unit. Based on scalability, each unit twin is subdivided into hub twins which were used to identify the data origin application used to gather data. The hub twin is identified by Hub-{name of the host computer}. This study used a unique identifier for the name of the daemon application. The hub twin has special permissions and authorisations to access the data sources. Based on demand and distribution, the application stimulates the edge twins in physical digital twin layer. The edge twin is added to the physical digital twin layer to indicate which data source attached to the daemon application. The edge twins were named as CCU{device identification number}. This study used barcode readers to simulate the data sources. In this study, however, an edge is defined as an interface for an electronic health record system, medical device, or observation forms that can be used to feed data into the framework. The session twin is a temporary 30-second virtual session used to hold information from barcode readers. During the testing phase, study recorded discrete events with millisecond accuracy as S-{timestamp in milliseconds}. However, this approach is not sustainable in the real world, where events might occur just microseconds or nanoseconds apart. To create unique names for each digital twin, this study decided to suffix a GUID as S-{GUID}. This solution addressed the uniqueness problem and avoided duplication issues even the data is ingested just nanoseconds apart.

The study designed the physical digital twin layer and respective cohorts as physical twins in the framework based on methods identified for gathering data. Due to time and scope limitations only barcode readers were used as a data source in this study, referred to as edge. Figure 5, shows the digital twin nodes and relationships between physical twins as represented in physical digital twin layer.

This study used Azure services such as Azure IoT Hub service (to establish and maintain connectivity between edge devices and the Azure cloud), Event Grid service (a publish and subscribe message distribution service to create event-driven serverless applications), and Function App service (for custom low-code serverless apps to develop custom middleware or interfaces between services). Also due to scope limitations, this study used barcode readers as edge devices. The daemon application was implemented in the host computer to act as a hub to listen for state changes in the data sources and send telemetry events to the Azure cloud. When a staff member used the edge device, the daemon application sent telemetry events regarding the task to the Azure IoT Hub service. Then, Event Grid service triggered Function App service to create new sessions and update or replace values for existing valid sessions. The session twins were used to store each discrete event. These session twins helped to virtually store and queue relevant information and were set to expire if the properties within them were incomplete or expired. Each session twin stored and validated properties such as patient identifier, staff identifier, form identifier, start time, and end time. Once the session twin validated the property data and it was mapped with conceptual twins, the session twin was set to expire and a snapshot of this instance was stored in the database for validation. The unit, hub, and edge twins were created or updated based on the telemetry event to point to the data source of the discrete event for validation purposes.

**Fig. 6:**
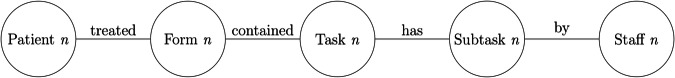
Conceptual digital twin layer. The figure shows digital twin nodes and relationships in the conceptual digital twin layer. The figure describes conceptual entities such as patient, form, task, subtask, and unit twins. The patient twin in the framework has an increment value suffixed as P-{increment number}, ensuring that each twin identification is unique in the conceptual digital twin layer. The patient identification value is derived from the session twin in physical digital twin layer. The form twin is named with incremental values such as F-{increment value}, which is an integer. Instead of using the full name of a form, such as “NGV1717 06/19 Individualised Care at the End of Life - Care Round Record Sheet (To be Used in Place of Enhanced Care Round Document)” this study shortened it to the form identification as F-50. This approach helped reduce errors and cleaned up the knowledge graph representation of the conceptual digital twins. The form identification value is derived from the session twin in physical digital twin layer. The task twin is an instance of the form twin. Further, subtask twin is used to record individual sessions within a task. The staff twin is used to identify staff members in the conceptual digital twin layer. These twins were identified using Globally Unique Identifiers and do not contain any demographic data to identify the personnel engaged in the trial. The unit twin is used to map each staff member to their workstation.

The conceptual digital twin layer defined conceptual twins, which were micro-ergonomic data such as rule, role, workflow, process, and standard twins associated with the critical care unit. The discrete event data originated from the physical digital twin layer and each snapshot was stored upon completion in a SQL database. The conceptual digital twin layer had interfaces with stakeholders, enabling interaction and generating reports through a web-based graphical user interface. As shown in Fig. 6, digital twin nodes and relationships between conceptual twins were represented in the conceptual digital twin layer.

The demand was created by patients when they were admitted to the unit for diagnosis and treatment. The staff were equipped with process maps to follow for each patient based on specific criteria. The staff used different types of observation forms to record these diagnosis and treatment procedures for the patient. Subsequently, the study was able to generate the framework for the conceptual digital twin layer by creating conceptual digital twins for these conceptual elements and mapping them based on the level of interaction using relationships.

Also, this study did not retrieve or store any patient-specific information or values from personal records. The form twin was added to the conceptual digital twin layer to understand the frequency of each observation form type. These form twins did not contain any patient-related data and were purely used to identify which form was used by the staff.

In the real-world setting, each observation form was used by several staff members over a particular period. For example, the “NGV1914 01/18 Critical Care Unit Daily Review” observation form was used by clinicians and nurses to record observations such as heart rate or blood pressure and treatments such as medications prescribed for a patient. This form was allocated every 24 hours and replaced with a new form at the end of the day. In the conceptual digital twin layer, this event was considered a task twin. If a patient stayed for 3 days, it means there were 3 task twins. Each task twin was attached to a patient and form twins recording the start and end times. This setup helped calculate bed acuity levels, costs per patient, the number of times a form was used, and the average task completion time. The conceptual digital twin layer only allowed one task twin for one form twin at any given instance, which helped to identify work duplication and track ongoing tasks. The task twin was mapped using the discrete event data stored in the session twin. Each task twin included a flag property which was used by staff to check if the task was completed or not. This resembled the process of changing observation forms in the physical setting. If there were no ongoing tasks, a new task twin would be created in the conceptual digital twin layer.

When a staff member begin treatment procedure, they first recorded the timestamp at the start of the procedure. They then recorded the second timestamp at the end of the procedure, which notified the conceptual digital twin layer that the subtask twin was completed. The conceptual digital twin layer enforced a rule that only one ongoing subtask is allowed for a specific task at a time by a staff member. Initially, a new subtask twin was created with both the start and end timestamps set to the same value. Once the subtask was completed, the end timestamp was updated, and the flag was set to true. This approach helped to map individual staff interactions with a given task, calculate the time a staff member spent on a subtask, assess whether a particular staff member was busy, track the timeline of interactions with a patient, and evaluate the costs and efficiency of the treatment process for each patient at a more granular level. The subtask twin did not contain any patient-related data and was purely used to record timestamps of the discrete event. The staff twin did not fetch or store any personal data such as names, ages, or other personal details. Due to scope and time limitations, this study did not store user roles either. Each staff twin was named using the format S-{increment value}. The staff twin would be created if it did not already exist, and it was then mapped to the relevant subtask twin based on the session twin.

Currently, the conceptual digital twin layer mapping was used to map staff who worked in the critical care unit. However, with scalability in mind, future staff from cross-departmental interactions—such as scanning X-rays or blood checking would be mapped based on available data to build a real-time, sophisticated knowledge graph representation of the hospital.

**Fig. 7:**
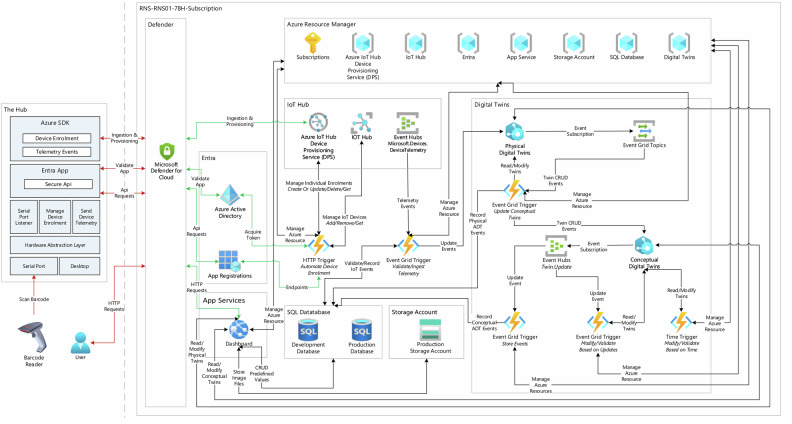
Azure cloud architecture. The daemon application establishes connectivity to the Azure cloud by dynamically generating symmetric keys for each edge device using application programming interface. This endpoint was protected by Microsoft Defender for Azure Cloud to identify malicious activities, and Microsoft Entra was used to secure accessibility to endpoints and scopes. The daemon application then retrieves symmetric keys to establish connectivity to the Azure IoT Hub Device Provisioning Service (DPS) dynamically based on the availability of edge devices. The cloud-based digital twins interfaced with users through edge devices and a web interface. The dynamic nature of allocating cloud resources required flexibility, study used Azure Resource Manager (Azure ARM) to allocate and generate security keys to establish connectivity between the services in real-time. By using function apps and event grids, the framework captured the event data of Azure IoT Hub and Azure Digital Twin instances and stored it in an Azure SQL database. The web interface provided users with accessibility to interact with digital twins. A set of temporary emails were created using Microsoft Entra to grant users access to the system. The figure contains official Azure architecture icons to communicate design and relationships between various Azure services used in this study. Microsoft permits the use of these icons in architectural diagrams. Source: https://learn.microsoft.com/en-us/azure/architecture/icons/.

**Fig. 8:**
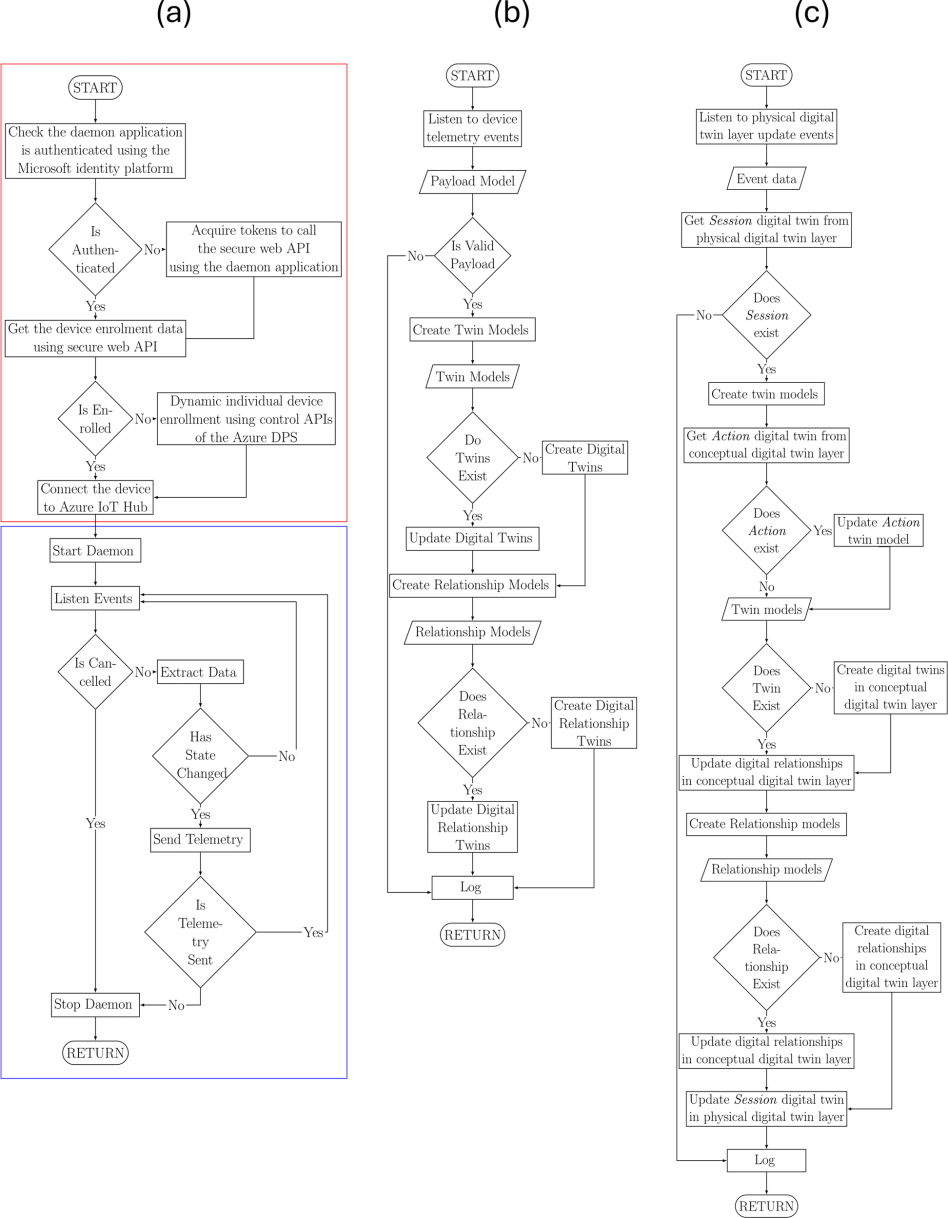
Flow diagrams of Internet of Things and Digital Twins. **a** Internet of Things: The Internet of Things consists of two modules, the cloud module and edge module. The cloud module, as highlighted in red, establishes a secure application interface using Azure Entra ID to interact with the REST API to enable dynamic device management in Azure IoT Hub and Azure IoT Hub Device Provisioning Service (DPS) services. The edge module, as highlighted in blue, creates separate threads to listen to data sources such as medical devices, electronic health records, or databases. The application continuously sends data to the Azure cloud until a cancellation token is received or retries in case of connection issues. **b** Physical twin flow: The function app listens to telemetry events in the Azure IoT Hub service. The flow extracts and verifies payload data and creates digital twins and relationships dynamically. **c** Conceptual twin flow: The function app listens to twin create and twin update events in the physical digital twin layer. The flow extracts session twins and creates digital twins and relationships dynamically. The thumbnails of observation forms were blurred for privacy reasons.

The software architecture was shown in Fig. 7, and the daemon application established interface to manage edge devices. Figure 8 shows flow diagrams of the daemon and event grid applications. When a staff member scanned values using an edge device, the daemon application created a telemetry event containing a JSON payload with information about the discrete event. Each telemetry event was then filtered by the Event Grid service to trigger functions to store this event data or ingest it into the physical digital twin instance based on their parameters. Then a series of function apps were designed to capture these digital twin update events in the physical digital twin instance using event grid triggers to modify the conceptual digital twin instance accordingly. The web-based dashboard provided an authenticated user interface for interacting with the conceptual digital twins, allowing users to generate reports such as identifying bed acuity, ongoing tasks and activities, costs by each form, costs by each patient, time taken by each form and staff member, and time spent on each patient. This study was limited in scope and did not build services such as notifying staff of ongoing activities or missing activities based on process maps. However, these potential expansions, such as time triggers, were identified and mentioned in the architecture for the future development.

In order not to disrupt the critical care unit workflows, this study deployed the framework on a separate system during the trial and evaluated it using a custom-built minicomputer to deploy the daemon application and connect the edge devices. The Supplementary Figs. 69–72 were output from the test stage and Supplementary Figs. 1–68 were output from the trial stage.

This study used barcode readers (*n* = 3), specifically Tera 51000 Laser 1D (*n* = 2) and Tera D5100 2D (*n* = 1) wireless barcode scanners as edge devices. Initially, the plan was to provide a scanner for each patient, but constraints limited the number due to the coverage limitations of the barcode readers. The main wall dividing the east ward and west ward posed a significant barrier to establishing connectivity between patients in farther sections with the daemon application. Additionally, the glass walls separating each bed caused signal disruptions between the daemon application and edge devices.

The consent forms were collected from the participant doctors (*n* = 15) and nurses (*n* = 5) at the Critical Care Unit of Northampton General Hospital NHS Trust. These consent forms were generic, not specific to referencing what type of information gathered or the type of task performed during this study. Each participant was identified by using custom numbers during the data collection period, which were only used by the staff to track the tasks performed for each patient. The collected samples did not include the demographic information of the participants due to privacy concerns. The study was evaluated using offline and online methods.

The offline method was deployed to overcome network issues caused by barriers between patients and the daemon application. In this method, staff members could either use edge devices or simply write on the paper the start and end times (see Supplementary Figs. 1–68). Later, data points written on the form were added to the digital twin framework using the online method. Figure 9 given below shows the process of data collection using the offline method.

**Fig. 9:**
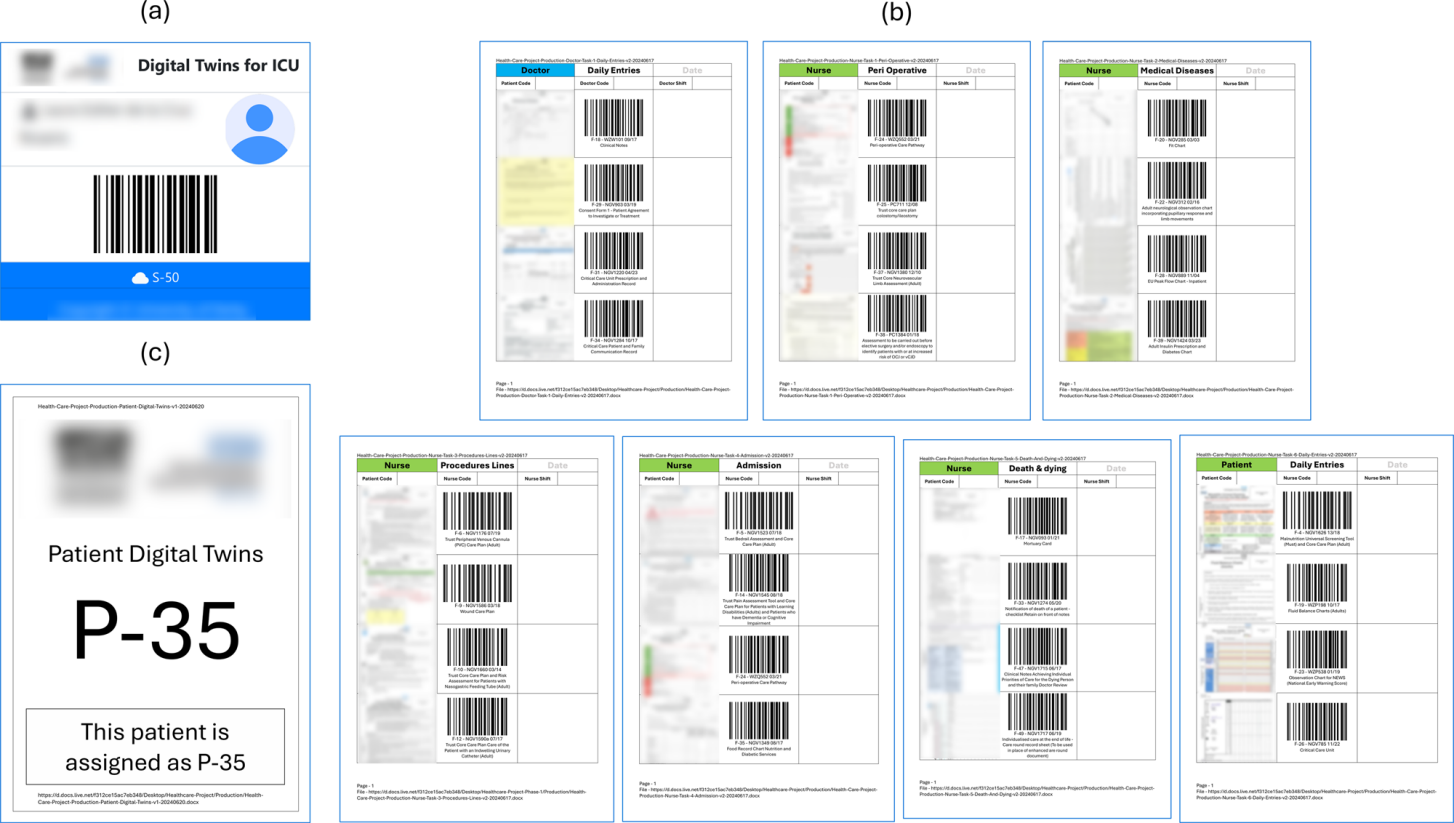
Data collection using offline method. This study included thumbnail-sized images of the front page of each observation form in the first column to help staff members easily identify the correct form. The second column contained a barcode with the form’s twin identification and descriptive name, allowing staff members to scan the barcode to automatically enter discrete events. The third column was designated for written comments or discrete time events. **a** A system-generated staff identification was created for each staff member, linked to a barcode. **b** A list of observation forms categorised by process groups was distributed to each staff member, allowing them to choose between using the online or offline method. The staff members could either record the start and end times of their subtask manually or use barcode readers. **c** Due to privacy concerns, this study did not use any real patient identification. Instead, each patient was assigned an incremental value, which was printed on a form. Each barcode reader was then assigned to an available patient based on connectivity, effectively mapping the barcode reader to the patient. This approach reduced the number of times a staff member had to record the time from three to two instances.

**Fig. 10:**
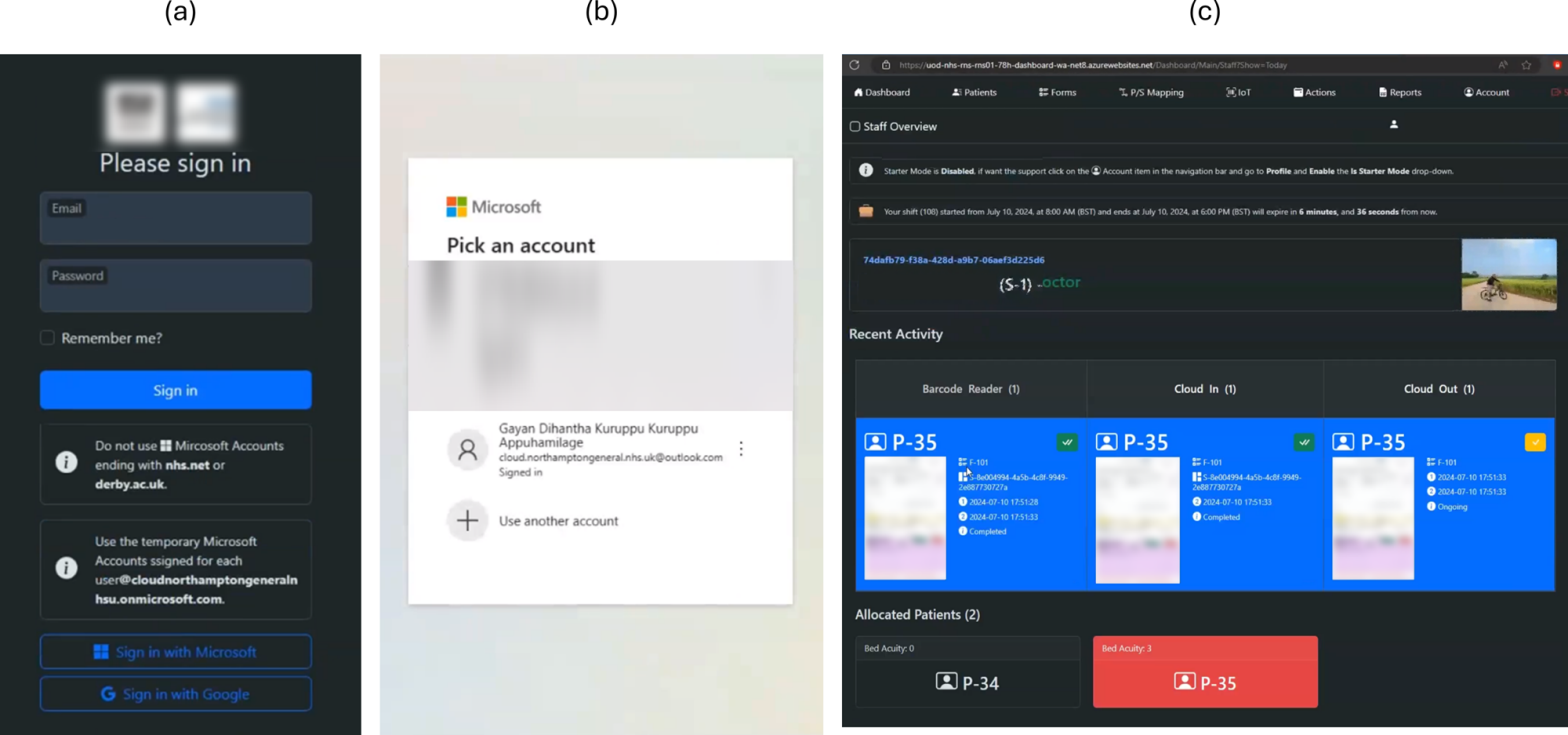
Data collection using the online method. **a** Study used Microsoft Entra to generate custom emails for logging into the system. An email was assigned with the staff member’s first name to make it easily readable when they logged into the web user interface. The web user interface was automatically enabled with Microsoft login, which was saved in their browser and authenticated using a password or the Microsoft Authenticator app, making it secure and familiar to use. The login screen displays additional login options such as custom email or password, default Microsoft authentication, and Google authentication. **b** The authentication screen appears before users accesses the dashboard. **c** The dashboard contains the user interface for interacting with the conceptual digital twin layer. The thumbnails of observation forms were blurred for privacy reasons.

Temporary emails were provided to each staff member to access the system through the web interface. Each barcode was assigned to a patient to allow staff to gather start and end times efficiently. This system helped track patients when they were moved to other beds based on the severity of their condition. The online method was primarily limited to those who already had access to computers. The online interface featured three user roles: managers, administrators, and staff. Figure 10 given below shows the web-based dashboard designed for staff.

Moreover, based on data collected in digital twins, unit managers could generate reports such as average time and costs related to each patient, bed acuity levels at a given time, and the costs generated by forms. Administrators could manage staff member access, create new users, oversee overall resource usage, allocate edge devices to patients, and manage resources. The staff interface allowed users to create, read, and update digital twins, allocate resources, monitor bed acuity levels, track ongoing tasks and subtasks for respective staff members, mark tasks or subtasks as complete, and create new subtasks based on forms.

## Dataset

The dataset was extracted using Digital Twins Query Language from Digital Twins Instances. Each event did not contain any data related to patients or staff, but rather workflows at the Critical Care Unit. This data included start and end timestamps of subtasks, observation form types, and digital twins’ names for staff and patients. This study cleaned the data in the digital twin instances into generalised records, showing the number of minutes spent on each task, categorised by process groups. The dataset was publicly accessible and was attached as a supplementary file for review purposes.

## Supplementary information

npj-Digital-Medicine-Supplementary-Material-v2-20250403

## Data Availability

The datasets generated during this study are attached to the Supplementary Information file.

## Code availability

The underlying repositories used in this study are not publicly available for proprietary reasons. We will make some parts of the framework’s source code available to the public in the next phases.
