# Supplementary material for: A health digital twin framework for discrete event simulation based optimised critical care workflows
Source: NPJ Digit Med. 2025 Jun 19;8:376. doi: 10.1038/s41746-025-01738-4 (PMC12179303; doi:10.1038/s41746-025-01738-4)
Supplement: Supplementary file 1 — npj-Digital-Medicine-Supplementary-Material-v2-20250403 [file 41746_2025_1738_MOESM1_ESM.pdf]

Supplementary Material

A health digital twin framework for discrete event simulation based optimised critical care workflows

Gayan Dihantha Kuruppu Kuruppu Appuhamilage (g.appuhamilage@derby.ac.uk) [1, *], Maqbool Hussain (m.hussain@derby.ac.uk) [1, *], Mohsin Zaman (mohsin.zaman@nhs.net) [2, *], Wajahat Ali Khan (w.khan@derby.ac.uk) [1]

[1] School of Computing, College of Science and Engineering, University of Derby, Kedleston Road, Derby, DE22 1GB, United Kingdom
[2] Northampton General Hospital NHS Trust, Cliftonville, Northampton NN1 5BD, United Kingdom
[*]Authors contributed equally and are recognized as co-lead authors.

Corresponding Author: Maqbool Hussain (m.hussain@derby.ac.uk)

Supplementary Table 1: Classification of Observation Forms used in Diagnosis and Treatment Tasks into Process Groups

Role	Process Map	Tasks
Doctor	Daily Entries	1) WZW101 09/17 Clinical Notes 2) NGV903 03/19 Consent Form 1 - Patient Agreement to Investigate or Treatment 3) NGV1220 04/23 Critical Care Unit Prescription and Administration Record 4) NGV1284 10/17 Critical Care Patient and Family Communication Record 5) NGV1550A 09/23 Treatment Escalation Plan (TEP) To be completed for all patients 6) NGV1914 01/18 Critical Care Unit Daily Review 7) NGV2031 11/20 Do not Attempt Cardiopulmonary Resuscitation 8) NGV2035 04/18 Critical Care Microbiology Report 9) NGV2064 07/22 Critical Care Unit Admission 10) NGV2343 06/21 Major Haemorrhage Protocol Blood Administration Record Dial 2222 11) Medical Admission Data<br>12) Critical Care Microbiology Results Record 13) Invasive Procedure Safety Checklist: Tracheostomy 14) Invasive Procedure Safety Checklist: CVC/Dialysis Catheter/PICC Insertion 15) Invasive Procedure Safety Checklist: Arterial Line 16) Procedure Checklist: Pruning 17) Mental Capacity Assessment 18) Patient Demographics 19) Parental Nutrition Prescription 20) Medical Discharge Data
Nurse	Daily Entries	1) FNGV1626 13/18 Malnutrition Universal Screening Tool (Must) and Core Care Plan (Adult) 2) WZP198 10/17 Fluid Balance Charts (Adults) 3) WZP538 01/19 Observation Chart for NEWS (National Early Warning Score) 4) NGV785 11/22 Critical Care Unit 5) NGV836 12/21 Adult Prescription and Administration Record 6) NGV1220 04/23 Critical Care Unit Prescription and Administration Record 7) NGV1284 10/17 Critical Care Patient and Family Communication Record 8) NGV1497 12/17 Patient Orientation Checklist - Nursing Staff to Complete 9) NGV1516 10/15 This is my Hospital Passport For people with learning disabilities coming to hospital 10) NGV1580 05/18 Adult Inpatient Admission Information Essential Assessments Activities of Daily Living Initial ADL Assessment 11) NGV1742 03/17 Critical Care Pressure Ulcer Prevention Assessment and Core Care Plan (Adult) 12) NGV1835 06/19 Nurse Handover/Transfer Safety Checklist for Receiving Ward 13) NGV1881 03/16 Duty of Candour Member of Staff carrying out Duty of Candour 14) NGV1914 01/18 Critical Care Unit Daily Review 15) NGV2431 08/21 Summary of Wound Care 16) NGV2441 04/22 Critical Care Nursing Transfer Form 17) NGV2444 03/23 Critical Care Eye Care Plan 18) NGV2485 01/22 Bowel Monitoring Care Plan 19) NGV2690 10/23 Critical Care Transfer Safety Checklist for Receiving Ward 20) Critical Care Water low Risk Assessment Score 21) NGV541 01/14 Trust Falls Assessment and Core Care Plan (Adult) 22) NGV1358 10/17 Trust Initial Pressure Ulcer Assessment (Adult)
	Peri-Operative	1) WZQ552 03/21 Peri-operative Care Pathway 2) PC711 12/08 Trust core care plan colostomy/ileostomy 3) NGV1380 12/10 Trust Core Neurovascular Limb Assessment (Adult)

		4) PC1384 01/18 Assessment to be carried out before elective surgery and/or endoscopy to identify patients with or at increased risk of OCJ or vCJD 5) NGV2431 08/21 Summary of Wound Care 6) F-105 Maintenance Check List Faecal Collection System
	Medical Diseases	1) NGV285 03/03 Fit Chart 2) NGV312 02/16 Adult neurological observation chart incorporating pupillary response and limb movements 3) NGV889 11/04 EU Peak Flow Chart – Inpatient 4) NGV1424 03/23 Adult Insulin Prescription and Diabetes 5) NGV1598 05/18 Diabetes sugar monitoring chart for patients not on insulin 6) Glasgow Modified Alcohol Withdrawal Scale (GMAWS)
	Procedure Lines	1) NGV1176 07/19 Trust Peripheral Venous Cannula (PVC) Care Plan (Adult) 2) NGV1586 03/18 Wound Care Plan 3) NGV1660 03/14 Trust Core Care Plan and Risk Assessment for Patients with Nasogastric Feeding Tube (Adult) 4) NGV1590a 07/17 Trust Core Care Plan Care of the Patient with an Indwelling Urinary Catheter (Adult) 5) NGV090 08/22 Adult Acute Pain Service Patient Controlled Analgesia 6) NGV1239 05/18 Trust Critical Care Arterial Cannula (AC) Care Plan 7) NGV1644 02/16 Trust Core Care Plan: Tracheostomy (Adult) 8) NGV1771 09/20 Adult Transfusion Prescription and Administration Record 9) NGV1798 10/20 Diabetic Ketoacidosis (DKA) Management for Adults 10) NGV1854 05/18 Intravenous Heparin Chart 11) Critical Care Therapies Treatment Record 12) Critical Care Continuous Renal Replacement Therapy Prescription Form & Chart for Citrate 13) MRI Patient Screening Questionnaire and Consent Form
	Admissions	1) NGV1523 07/18 Trust Bedrail Assessment and Core Care Plan (Adult) 2) NGV1545 08/18 Trust Pain Assessment Tool and Core Care Plan for Patients with Learning Disabilities (Adults) and Patients who have Dementia or Cognitive Impairment 3) WZQ552 03/21 Peri-operative Care Pathway 4) NGV1349 08/17 Food Record Chart Nutrition and Diabetic Services 5) NGV2109 07/20 Critical Care Nursing Assessments and Care Plans 6) Critical Care Patient Property Form 7) Parental Nutrition: Initial Review Form 8) Trust Core Patient Activities of Daily Living - Initial Assessment
	Death and dying	1) NGV093 01/21 Mortuary Card 2) NGV1274 05/20 Notification of death of a patient - checklist Retain on front of notes 3) NGV1715 06/17 Clinical Notes Achieving Individual Priorities of Care for the Dying Person and their family Doctor Review 4) NGV1717 06/19 Individualised care at the end of life - Care round record sheet (To be used in place of enhanced are round document) 5) NGV1718 03/18 Clinical Notes Individualised Care for the Dying Person and their family (Continued) 6) NGV1720 08/17 Individualised Plan of Care for the Dying Person and their Family Multidisciplinary Communication Sheet 7) NGV2245 04/20 Death Verification Report 8) Tissue Donation Referral Form – Email 9) H. M. Coroner - Referral Form 10) End of life care checklist

Supplementary Table 1: The classification of observation forms used in diagnosis and treatment tasks used by doctor and nurse staff roles into process groups

This study sorted unstructured tasks related to doctors into process maps (n=1) using forms to record daily entries (n=20) and structured tasks related to nurses process maps (n=6) using forms to document 1) daily entries (n=22) 2) peri-operative tasks (n=6) 3) medical diseases (n=6) 4) procedure lines (n=13) 5) admissions (n=8) 6) death and dying processes (n=10).

Supplementary Table 2: Doctor’s Process Group 1 – Daily Entries

Shift	F-18 (Clinical Notes)	F-29 (Consent Form 1 - Patient Agreement to Investigate or Treatment)	F-31 (Critical Care Unit Prescription and Administration Record)	F-34 (Critical Care Patient and Family Communication Record)	F-42 (Treatment Escalation Plan (TEP) To be completed for all patients)	F-58 (Critical Care Unit Daily Review)	F-59 (Do not Attempt Cardiopulmonary Resuscitation)	F-60 (Critical Care Microbiology Report)	F-62 (Critical Care Unit Admission)	F-65 (Major Haemorrhage Protocol Blood Administration Record Dial 2222)	F-75 (Medical Admission Data)	F-81 (Critical Care Microbiology Results Record)	F-90 (Invasive Procedure Safety Checklist: Tracheostomy)	F-91 (Invasive Procedure Safety Checklist: CVC/Dialysis Catheter/PICC Insertion)	F-92 (Invasive Procedure Safety Checklist: Arterial Line)	F-93 (Procedure Checklist: Proning)	F-98 (Mental Capacity Assessment)	F-99 (Patient Demographics)	F-102 (Parental Nutrition Prescription)	F-103 (Medical Discharge Data)	F108 (Procedure Checklist: Un-Pronin)	F-109 Procedure Intubation
1	0	0	0	0	5	0	0	0	0	0	0	0	0	0	0	0	0	0	0	0	0	0
2	0	0	0	0	0	20	0	0	0	0	0	0	0	0	0	0	0	0	0	0	0	0
3	0	0	0	0	0	15	0	0	0	0	0	0	0	0	0	0	0	0	0	0	0	0
4	0	0	0	0	5	0	0	0	0	0	0	0	0	0	0	0	0	0	0	0	0	0
5	0	0	0	15	0	23	0	0	0	0	0	0	0	0	0	0	0	0	0	0	0	0
6	0	0	0	0	0	10	0	0	0	0	0	0	0	0	0	0	0	0	0	0	0	15
7	15	0	3	0	0	0	0	0	0	0	0	0	0	0	0	0	0	0	0	0	0	0
8	0	0	0	0	0	0	0	0	0	0	0	0	0	17	0	0	0	0	0	0	0	0
9	30	0	0	0	0	0	0	0	0	0	0	0	0	0	0	0	0	0	0	0	0	10
10	5	0	0	0	0	0	0	0	0	0	0	0	0	0	0	0	0	0	0	0	0	0
11	0	0	0	0	0	30	0	0	0	0	0	0	0	0	0	0	0	0	0	0	0	0
12	0	0	0	0	0	0	0	0	0	0	0	0	0	0	0	0	0	0	0	0	0	0
13	0	0	0	0	0	0	0	0	0	0	0	0	0	0	0	0	0	0	0	0	0	0
14	0	0	0	0	0	0	0	0	0	0	0	0	0	0	0	0	0	0	0	0	0	0
15	0	0	0	0	0	0	0	0	0	0	0	0	0	0	0	0	0	0	0	0	0	0
**Avg**	**16.66**	**0**	**3**	**15**	**5**	**19.66**	**0**	**0**	**0**	**0**	**0**	**0**	**0**	**17**	**0**	**0**	**0**	**0**	**0**	**0**	**0**	**12.5**
**Min**	**5**	**0**	**3**	**15**	**5**	**10**	**0**	**0**	**0**	**0**	**0**	**0**	**0**	**17**	**0**	**0**	**0**	**0**	**0**	**0**	**0**	**10**
**Max**	**30**	**0**	**3**	**15**	**5**	**30**	**0**	**0**	**0**	**0**	**0**	**0**	**0**	**17**	**0**	**0**	**0**	**0**	**0**	**0**	**0**	**15**
**Count**	**3**	**0**	**1**	**1**	**2**	**5**	**0**	**0**	**0**	**0**	**0**	**0**	**0**	**1**	**0**	**0**	**0**	**0**	**0**	**0**	**0**	**2**

Supplementary Table 2: The takt time analysis for the daily entries process group performed by doctors during each shift

The data is summarised based on Supplementary Figures 50 – 68 which were recorded by doctors during data collection period. The data shows discrete events of each daily entry task and time is mentioned in minutes and seconds. During the data cleanup phase, the study used value “0” to indicate no recorded values. The column header shows a combination of form twin and the name of each observation form used by doctors at critical care unit. The Avg, Min, Max, and Count rows below the table provides a summary of each daily entry task type.

Supplementary Table 3: Nurse's Process Group 1 – Peri Operative

Shift	F-24 (Peri-operative Care Pathway)	F-25 (Trust core care plan colostomy / ileostomy)	F-37 (Trust Core Neurovascular Limb Assessment (Adult))	F-38 (Assessment to be carried out before elective surgery and/or endoscopy to identify patients with or at increased risk of OCJ or vCJD)	F-66 (Summary of Wound Care)	F-105 (Maintenance Check List Faecal Collection System)
1	0	0	3	0	0	0
2	0	0	2	0	1	0
3	0	0	0	0	0	0
4	0	0	0	0	0	0
5	0	0	0	0	0	0
6	0	0	0	0	0	0
7	0	0	0	0	0	0
8	0	0	0	0	0	0
9	0	0	0	0	0	0
10	0	0	0	0	0	0
11	0	0	0	0	0	0
12	0	0	0	0	0	0
13	0	0	0	0	0	0
14	0	0	0	0	0	0
15	0	0	0	0	0	0
**Avg**	**0**	**0**	**2.5**	**0**	**1**	**0**
**Min**	**0**	**0**	**2**	**0**	**1**	**0**
**Max**	**0**	**0**	**3**	**0**	**1**	**0**
**Count**	**0**	**0**	**2**	**0**	**1**	**0**

Supplementary Table 3: The takt time analysis for the peri-operative process group performed by nurses during each shift

The data is summarised based on Supplementary Figures 1 – 6 which were recorded by nurses during data collection period. The data shows discrete events of each peri-operative task and time is mentioned in minutes and seconds. During the data cleanup phase, the study used value "0" to indicate no recorded values. The column header shows a combination of form twin and the name of each observation form used by nurses at critical care unit. The Avg, Min, Max, and Count rows below the table provides a summary of each peri-operative task type.

Supplementary Table 4: Nurse's Process Group 2 – Medical Diseases

Shift	F-20 (Fit Chart)	F-22 (Adult neurological observation chart incorporating pupillary response and limb movements)	F-28 (EU Peak Flow Chart - Inpatient)	F-39 (Adult Insulin Prescription and Diabetes Chart)	F-45 (Diabetes sugar monitoring chart for patients not on insulin)	F-100 (Glasgow Modified Alcohol Withdrawal Scale (GMAWS))
1	0	0	0	0	0	0
2	0	0	0	0	0	0
3	0	0	0	0	0	0
4	0	0	0	0	0	0
5	0	0	0	0	0	0
6	0	0	0	0	0	0
7	0	0	0	0	0	0
8	0	0	0	0	0	0
9	0	0	0	0	0	0
10	0	0	0	0	0	0
11	0	0	0	0	0	0
12	0	0	0	0	0	0
13	0	0	0	0	0	0
14	0	0	0	0	0	0
15	0	0	0	0	0	0
**Avg**	**0**	**0**	**0**	**0**	**0**	**0**
**Min**	**0**	**0**	**0**	**0**	**0**	**0**
**Max**	**0**	**0**	**0**	**0**	**0**	**0**
**Count**	**0**	**0**	**0**	**0**	**0**	**0**

Supplementary Table 4: The takt time analysis for the medical diseases process group performed by nurses during each shift

The data is summarised based on Supplementary Figures 7 – 12 which were recorded by nurses during data collection period. The data shows discrete events of each medical diseases task and time is mentioned in minutes and seconds. During the data cleanup phase, the study used value "0" to indicate no recorded values. The column header shows a combination of form twin and the name of each observation form used by nurses at critical care unit. The Avg, Min, Max, and Count rows below the table provides a summary of each medical diseases task type. The data was not recorded for medical diseases tasks during the data collection period.

Supplementary Table 5: Nurse's Process Group 3 – Procedure Lines

Shift	F-6 (Trust Peripheral Venous Cannula (PVC) Care Plan (Adult))	F-9 (Wound Care Plan)	F-10 (Trust Core Care Plan and Risk Assessment for Patients with Nasogastric Feeding Tube (Adult))	F-12 (Trust Core Care Plan Care of the Patient with an Indwelling Urinary Catheter (Adult))	F-16 (Adult Acute Pain Service Patient Controlled Analgesia)	F-32 (Trust Critical Care Arterial Cannula (AC) Care Plan)	F-46 (Trust Core Care Plan: Tracheostomy (Adult))	F-53 (Adult Transfusion Prescription and Administration Record)	F-54 (Diabetic Ketoacidosis (DKA) Management for Adults)	F-56 (Intravenous Heparin Chart)	F-72 (Critical Care Therapies Treatment Record)	F-84 (Critical Care Continuous Renal Replacement Therapy Prescription Form & Chart for Citrate)	F-97 (MRI Patient Screening Questionnaire and Consent Form)
1	4	0	0	2	0	3	0	0	0	0	0	0	0
2	1	0	2	1	0	1	0	0	0	0	0	0	0
3	3	0	0	2	0	2	0	0	0	0	0	0	0
4	1	2	2	1	0	3	0	0	0	0	0	0	0
5	1	0	0	1	0	1	0	0	0	0	0	0	0
6	0	0	0	0	0	0	0	0	0	0	0	0	0
7	0	0	0	0	0	0	0	0	0	0	0	0	0
8	0	0	0	0	0	0	0	0	0	0	0	0	0
9	0	0	0	0	0	0	0	0	0	0	0	0	0
10	0	0	0	0	0	0	0	0	0	0	0	0	0
11	0	0	0	0	0	0	0	0	0	0	0	0	0
12	0	0	0	0	0	0	0	0	0	0	0	0	0
13	0	0	0	0	0	0	0	0	0	0	0	0	0
14	0	0	0	0	0	0	0	0	0	0	0	0	0
15	0	0	0	0	0	0	0	0	0	0	0	0	0
**Avg**	**2**	**2**	**2**	**1.4**	**0**	**2**	**0**	**0**	**0**	**0**	**0**	**0**	**0**
**Min**	**1**	**2**	**2**	**1**	**0**	**1**	**0**	**0**	**0**	**0**	**0**	**0**	**0**
**Max**	**4**	**2**	**2**	**2**	**0**	**3**	**0**	**0**	**0**	**0**	**0**	**0**	**0**
**Count**	**5**	**1**	**2**	**5**	**0**	**5**	**0**	**0**	**0**	**0**	**0**	**0**	**0**

Supplementary Table 5: The takt time analysis for the procedure lines process group performed by nurses during each shift

The data is summarised based on Supplementary Figures 13 – 30 which were recorded by nurses during data collection period. The data shows discrete events of each procedure lines task and time is mentioned in minutes and seconds. During the data cleanup phase, the study used value "0" to indicate no recorded values. The column header shows a combination of form twin and the name of each observation form used by nurses at critical care unit. The Avg, Min, Max, and Count rows below the table provides a summary of each procedure lines task type.

Supplementary Table 6: Nurse's Process Group 4 – Admission

Shift	F-5 (Trust Bedrail Assessment and Core Care Plan (Adult))	F-14 (Trust Pain Assessment Tool and Core Care Plan for Patients with Learning Disabilities (Adults) and Patients who have Dementia or Cognitive Impairment)	F-24 (Peri-operative Care Pathway)	F-35 (Food Record Chart Nutrition and Diabetic Services)	F-63 (Critical Care Nursing Assessments and Care Plans)	F-77 (Critical Care Patient Property Form)	F-101 (Parental Nutrition: Initial Review Form)	F-113 (Trust Core Patient Activities of Daily Living - Initial Assessment)
1	1	0	0	0	20	3	0	4
2	1	0	0	0	25	0	0	0
3	1	0	0	2	25	0	0	0
4	1	0	0	0	60[1]	0	0	0
5	0	0	0	0	0	0	0	0
6	0	0	0	0	0	0	0	0
7	0	0	0	0	0	0	0	0
8	0	0	0	0	0	0	0	0
9	0	0	0	0	0	0	0	0
10	0	0	0	0	0	0	0	0
11	0	0	0	0	0	0	0	0
12	0	0	0	0	0	0	0	0
13	0	0	0	0	0	0	0	0
14	0	0	0	0	0	0	0	0
15	0	0	0	0	0	0	0	0
**Avg**	**1**	**0**	**0**	**2**	**23.33**	**3**	**0**	**4**
**Min**	**1**	**0**	**0**	**2**	**20**	**3**	**0**	**4**
**Max**	**1**	**0**	**0**	**2**	**25**	**3**	**0**	**4**
**Count**	**4**	**0**	**0**	**1**	**3**	**1**	**0**	**1**

Supplementary Table 6: The takt time analysis for the admission process group performed by nurses during each shift

The data is summarised based on Supplementary Figures 31 – 40 which were recorded by nurses during data collection period. The data shows discrete events of each admission task and time is mentioned in minutes and seconds. During the data cleanup phase, the study used value "0" to indicate no recorded values. The column header shows a combination of form twin and the name of each observation form used by nurses at critical care unit. The Avg, Min, Max, and Count rows below the table provides a summary of each admission task type.

[1] *Staff member reported the total number of total times spent on F-63 (Critical Care Nursing Assessments and Care Plans) including interruptions during that period.*

Supplementary Table 7: Nurse's Process Group 5 – Death and Dying

Shift	F-17 (Mortuary Card)	F-33 (Notification of death of a patient - checklist Retain on front of notes)	F-47 (Clinical Notes Achieving Individual Priorities of Care for the Dying Person and their family Doctor Review)	F-49 (Individualised care at the end of life - Care round record sheet (To be used in place of enhanced are round document))	F-50 (Clinical Notes Individualised Care for the Dying Person and their family (Continued))	F-51 (Individualised Plan of Care for the Dying Person and their Family Multidisciplinary Communication Sheet)	F-64 (Death Verification Report)	F-71 (Tissue Donation Referral Form - Email)	F-83 (H. M. Coroner - Referral Form)	F-85 (End of life care checklist)
1	0	0	0	0	0	0	0	0	0	0
2	0	0	0	0	0	0	0	0	0	0
3	0	0	0	0	0	0	0	0	0	0
4	0	0	0	0	0	0	0	0	0	0
5	0	0	0	0	0	0	0	0	0	0
6	0	0	0	0	0	0	0	0	0	0
7	0	0	0	0	0	0	0	0	0	0
8	0	0	0	0	0	0	0	0	0	0
9	0	0	0	0	0	0	0	0	0	0
10	0	0	0	0	0	0	0	0	0	0
11	0	0	0	0	0	0	0	0	0	0
12	0	0	0	0	0	0	0	0	0	0
13	0	0	0	0	0	0	0	0	0	0
14	0	0	0	0	0	0	0	0	0	0
15	0	0	0	0	0	0	0	0	0	0
**Avg**	**0**	**0**	**0**	**0**	**0**	**0**	**0**	**0**	**0**	**0**
**Min**	**0**	**0**	**0**	**0**	**0**	**0**	**0**	**0**	**0**	**0**
**Max**	**0**	**0**	**0**	**0**	**0**	**0**	**0**	**0**	**0**	**0**
**Count**	**0**	**0**	**0**	**0**	**0**	**0**	**0**	**0**	**0**	**0**

Supplementary Table 7: The takt time analysis for the death and dying process group performed by nurses during each shift

The data is summarised based on Supplementary Figures 41 – 49 which were recorded by nurses during data collection period. The data shows discrete events of each death and dying task and time is mentioned in minutes and seconds. During the data cleanup phase, the study used value "0" to indicate no recorded values. The column header shows a combination of form twin and the name of each observation form used by nurses at critical care unit. The Avg, Min, Max, and Count rows below the table provides a summary of each death and dying task type. The data was not recorded for death and dying tasks during the data collection period.

Supplementary Table 8: Nurse's Process Group 6 – Daily Entries

Shift	F-4 (Malnutrition Universal Screening Tool (Must) and Core Care Plan (Adult))	F-19 (Fluid Balance Charts (Adults))	F-23 (Observation Chart for NEWS (National Early Warning Score))	F-26 (Critical Care Unit)	F-27 (Adult Prescription and Administration Record)	F-31(Critical Care Unit Prescription and Administration Record)	F-34(Critical Care Patient and Family Communication Record)	F-40 (Patient Orientation Checklist - Nursing Staff to Complete)	F-41(This is my Hospital Passport For people with learning disabilities coming to hospital)	F-44 (Adult Inpatient Admission Information Essential Assessments Activities of Daily Living Initial ADL Assessment)	F-52 (Critical Care Pressure Ulcer Prevention Assessment and Core Care Plan	F-55 (Nurse Handover/Transfer Safety Checklist for Receiving Ward)	F-57 (Duty of Candour Member of Staff carrying out Duty of Candour)	F-58 (Critical Care Unit Daily Review)	F-66 (Summary of Wound Care)	F-67 (Critical Care Nursing Transfer Form)	F-68 (Critical Care Eye Care Plan)	F-69 (Bowel Monitoring Care Plan)	F-70 (Critical Care Transfer Safety Checklist for receiving Ward)	F-94 (Critical Care Water Low Risk Assessment Score)	F-111 (Trust Falls Assessment and Core Care Plan Adult))	F-112 (Trust Initial Pressure Ulcer Assessment (Adult))
1	0	0	0	0	0	0	0	0	0	0	0	0	0	0	0	0	0	0	0	0	0	0
2	0	0	0	0	0	0	0	0	0	0	0	0	0	0	0	0	0	0	0	0	0	0
3	0	0	0	0	0	0	0	0	0	0	0	0	0	0	0	0	0	0	0	0	0	0
4	0	0	0	0	0	0	0	0	0	0	0	0	0	0	0	0	0	0	0	0	0	0
5	0	0	0	0	0	0	0	0	0	0	0	0	0	0	0	0	0	0	0	0	0	0
6	0	0	0	0	0	0	0	0	0	0	0	0	0	0	0	0	0	0	0	0	0	0
7	0	0	0	0	0	0	0	0	0	0	0	0	0	0	0	0	0	0	0	0	0	0
8	0	0	0	0	0	0	0	0	0	0	0	0	0	0	0	0	0	0	0	0	0	0
9	0	0	0	0	0	0	0	0	0	0	0	0	0	0	0	0	0	0	0	0	0	0
10	0	0	0	0	0	0	0	0	0	0	0	0	0	0	0	0	0	0	0	0	0	0
11	0	0	0	0	0	0	0	0	0	0	0	0	0	0	0	0	0	0	0	0	0	0
12	0	0	0	0	0	0	0	0	0	0	0	0	0	0	0	0	0	0	0	0	0	0
13	0	0	0	0	0	0	0	0	0	0	0	0	0	0	0	0	0	0	0	0	0	0
14	0	0	0	0	0	0	0	0	0	0	0	0	0	0	0	0	0	0	0	0	0	0
15	0	0	0	0	0	0	0	0	0	0	0	0	0	0	0	0	0	0	0	0	0	0
**Avg**	**0**	**0**	**0**	**0**	**0**	**0**	**0**	**0**	**0**	**0**	**0**	**0**	**0**	**0**	**0**	**0**	**0**	**0**	**0**	**0**	**0**	**0**
**Min**	**0**	**0**	**0**	**0**	**0**	**0**	**0**	**0**	**0**	**0**	**0**	**0**	**0**	**0**	**0**	**0**	**0**	**0**	**0**	**0**	**0**	**0**
**Max**	**0**	**0**	**0**	**0**	**0**	**0**	**0**	**0**	**0**	**0**	**0**	**0**	**0**	**0**	**0**	**0**	**0**	**0**	**0**	**0**	**0**	**0**
**Count**	**0**	**0**	**0**	**0**	**0**	**0**	**0**	**0**	**0**	**0**	**0**	**0**	**0**	**0**	**0**	**0**	**0**	**0**	**0**	**0**	**0**	**0**

Supplementary Table 8: The takt time analysis for the daily entry process group performed by nurses during each shift

The data was not recorded for daily entry tasks during the data collection period. The data shows discrete events of each daily entry task and time is mentioned in minutes and seconds. During the data cleanup phase, the study used value "0" to indicate no recorded values. The column header shows a combination of form twin and the name of each observation form used by nurses at critical care unit. The Avg, Min, Max, and Count rows below the table provides a summary of each daily entry task type.

Supplementary Table 9: Internet of Things

Timestamp	Telemetry messages sent (Sum)	Event Grid deliveries (Sum)	Routing: message latency for messages/events (Avg)	Event Grid latency (Avg)
20/06/2024 12:00	66	66	149.12	179.39
20/06/2024 18:00	7	7	208.42	237.42
21/06/2024 00:00	4	4	188	188.25
21/06/2024 06:00	23	23	155.78	190.39
21/06/2024 12:00	4	4	166.50	213.25
21/06/2024 18:00	43	43	164.37	206.41
22/06/2024 00:00	74	74	150.09	185.14
22/06/2024 06:00	23	23	166.26	173.56
22/06/2024 12:00	27	27	181.14	197.33
22/06/2024 18:00	27	27	147.03	175.29
23/06/2024 00:00	79	79	148.46	159.37
23/06/2024 06:00	5	5	208.8	202.60
24/06/2024 12:00	3	3	203	198
25/06/2024 12:00	5	5	234	183.80
26/06/2024 18:00	6	6	186.66	173.83
27/06/2024 06:00	3	3	181.66	234
27/06/2024 18:00	4	4	162	181.50

Supplementary Table 9: The Routing and Event Grid latency of the Azure IoT Hub service was analysed using data extracted from the Azure cloud Analytics service

The "Timestamp" column displays time intervals of 6 hours from 2024/06/20 to 2024/06/27. The "Telemetry messages sent (Sum)" column describes total number of device toc loud messages sent to Azure IoT Hub service. The "Event Grid deliveries (Sum)" column describes total number of Azure IoT Hub events published to Event Grid. The "Routing: message latency for messages/events (Avg)" column describes the average latency in milliseconds taken to ingress event to Azure IoT Hub to built-in endpoint. The "Event Grid latency (Avg)" column describes the average latency from when Azure IoT Hub generate the event to publish to Event Grid. The data was not completely recorded for 23/06/2024, 24/06/2024, 25/06/2024, 26/06/2024, and 27/06/2024 due to lack of activity.

Supplementary Table 10: Digital Twin Layers (Latency)

Timestamp	nhs-rns-rns01-78h-phsyical-twins-adt		nhs-rns-rns01-78h-conceptual-twins-adt	
	API Requests Latency (Avg)	Routing Latency (Avg)	API Requests Latency (Avg)	Routing Latency (Avg)
20/06/24 12:00	12.50	319.57	113.61	112.03
20/06/24 18:00	36.07	348.56	31.25	115.52
21/06/24 00:00	13.66	32.32	32.11	121.59
21/06/24 06:00	15.24	16.06	29.00	109.88
21/06/24 12:00	64.59	15.40	23.86	110.42
21/06/24 18:00	29.52	228.14	23.66	113.06
22/06/24 00:00	141.49	201.99	36.14	117.45
22/06/24 06:00	29.04	189.96	30.04	116.87
22/06/24 12:00	35.24	148.26	239.33	118.07
22/06/24 18:00	26.04	365.75	301.09	113.45
23/06/24 00:00	23.55	399.40	223.71	106.08
23/06/24 06:00	23.19	37.49	338.21	102.35
26/06/24 18:00	208.50	125.79	39.29	151.79
27/06/24 18:00	184	159.55	35.08	221.36

Supplementary Table 10: The API Request (milliseconds) and Routing latency (milliseconds) of physical and conceptual digital twin instances

The "Timestamp" column displays time intervals of 6 hours from 2024/06/20 to 2024/06/27. The columns "nhs-rns-rns01-78h-phsyical-twins-adt" shows metrics for physical digital twin layer and "nhs-rns-rns01-78h-conceptual-twins-adt" shows metrics for conceptual digital twin layer. The "API Requests Latency (AVG)" column describes the average response time in milliseconds for API requests. The response time refers total time Azure Digital Twins has taken to process read, write, delete, and query operations from request to response success/fail result. The "Routing Latency (AVG)" column describes average routing latency in milliseconds for routing. The routing latency describes time elapsed from event get routed to azure Digital Twins and posted to event endpoint. The data was not completely recorded for 23/06/2024, 24/06/2024, 25/06/2024, 26/06/2024, and 27/06/2024 due to lack of activity.

Supplementary Table 11: Digital Twin Layers (Triggers)

c	Function Count (Sum)			
	IoT-Hub To SQL-DB	IoT-Hub To Physical-Twins-ADT	Physical-Twins-ADT To Conceptual-Twins-ADT	Conceptual-Twins-ADT To SQL-DB
20/06/2024 12:00	66	66	66	20
20/06/2024 18:00	7	7	7	6
21/06/2024 00:00	4	4	4	4
21/06/2024 06:00	23	23	23	22
21/06/2024 12:00	4	4	4	4
21/06/2024 18:00	43	43	43	30
22/06/2024 00:00	74	74	74	46
22/06/2024 06:00	23	23	23	8
22/06/2024 12:00	27	27	27	6
22/06/2024 18:00	27	27	27	20
23/06/2024 00:00	79	79	79	62
23/06/2024 06:00	5	5	5	2
24/06/2024 12:00	3	3	3	0
25/06/2024 12:00	5	5	5	0
26/06/2024 18:00	6	6	5	4
27/06/2024 06:00	3	3	3	0
27/06/2024 18:00	4	4	4	7

Supplementary Table 11: The function trigger counts of Internet of Things and digital twin instances during the data collection period

The "Timestamp" column displays time intervals of 6 hours from 2024/06/20 to 2024/06/27. The "IoT-Hub to SQL-DB" column represents the total number of events used to store each telemetry event in the SQL database. The "IoT-Hub to Physical-Twins-ADT" column indicates the total number of ingestion events to the physical digital twin layer. The "Physical-Twins-ADT to Conceptual-Twins-ADT" column shows the total number of ingestion events to the conceptual digital twin layer. The "Conceptual-Twins-ADT to SQL-DB" column represents the total number of events used to store digital twin update events in the SQL database. The data was not completely recorded for 23/06/2024, 24/06/2024, 25/06/2024, 26/06/2024, and 27/06/2024 due to lack of activity.

Supplementary Table 12: Digital Twin Layers (Count)

Timestamp	nhs-rns-rns01-78h-physical-twins-adt	nhs-rns-rns01-78h-conceptual-twins-adt
	Twin Count (Sum)	Twin Count (Sum)
20/06/2024 12:00	67	48
20/06/2024 18:00	70	49
21/06/2024 00:00	72	50
21/06/2024 06:00	83	55
21/06/2024 12:00	85	56
21/06/2024 18:00	107	67
22/06/2024 00:00	144	85
22/06/2024 06:00	155	90
22/06/2024 12:00	169	97
22/06/2024 18:00	193	111
23/06/2024 00:00	233	131
23/06/2024 06:00	235	132
26/06/2024 18:00	242	135
27/06/2024 18:00	245	136

Supplementary Table 12: The total number of digital twins allocated during the data collection period

The "Timestamp" column displays time intervals of 6 hours from 2024/06/20 to 2024/06/27. The "IoT-Hub to SQL-DB" column represents the total number of events used to store each telemetry event in the SQL database. The "nhs-rns-rns01-78h-physical-twins-adt" column indicates the total twin count in the physical digital twin layer. The "nhs-rns-rns01-78h-conceptual-twins-adt" column indicates the total twin count in the conceptual digital twin layer. The data was not completely recorded for 23/06/2024, 24/06/2024, 25/06/2024, 26/06/2024, and 27/06/2024 due to lack of activity.

Supplementary Figure 1: Result Page 1

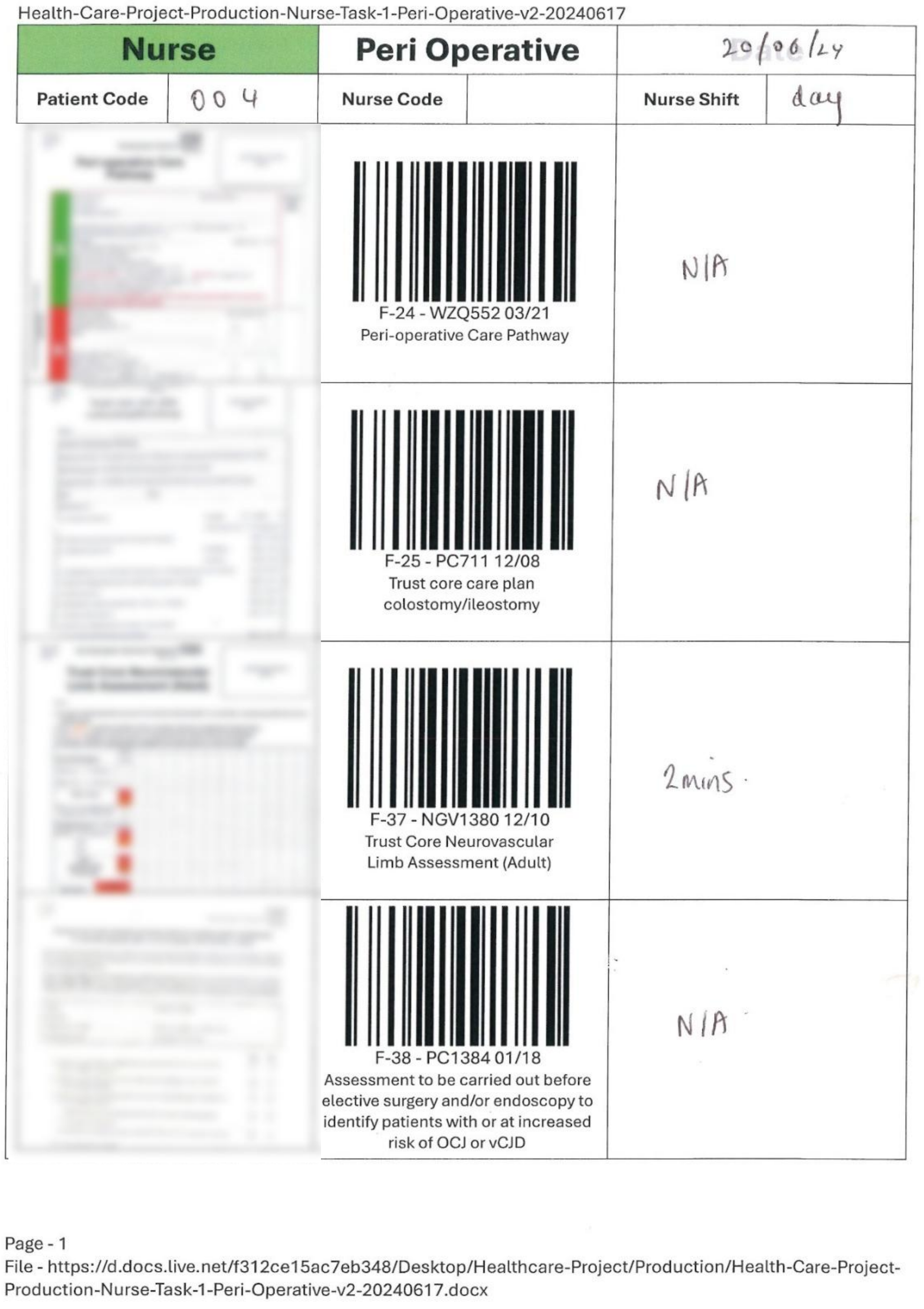

Health-Care-Project-Production-Nurse-Task-1-Peri-Operative-v2-20240617

Nurse		Peri Operative		Date 20/06/24	
Patient Code	004	Nurse Code		Nurse Shift	day

	F-24 - WZQ552 03/21<br>Peri-operative Care Pathway	N/A
	F-25 - PC711 12/08<br>Trust core care plan colostomy/ileostomy	N/A
	F-37 - NGV1380 12/10<br>Trust Core Neurovascular Limb Assessment (Adult)	2mins.
	F-38 - PC1384 01/18<br>Assessment to be carried out before elective surgery and/or endoscopy to identify patients with or at increased risk of OCJ or vCJD	N/A

Page - 1

File - https://d.docs.live.net/f312ce15ac7eb348/Desktop/Healthcare-Project/Production/Health-Care-Project-Production-Nurse-Task-1-Peri-Operative-v2-20240617.docx

Supplementary Figure 1: Result page – 1 for peri-operative discrete event.

This peri-operative discrete event was recorded using offline method by nursing staff. The demographic data was excluded in during the data collection.

The thumbnails of observation forms used in this figure were blurred for privacy reasons.

Supplementary Figure 2: Result Page 2

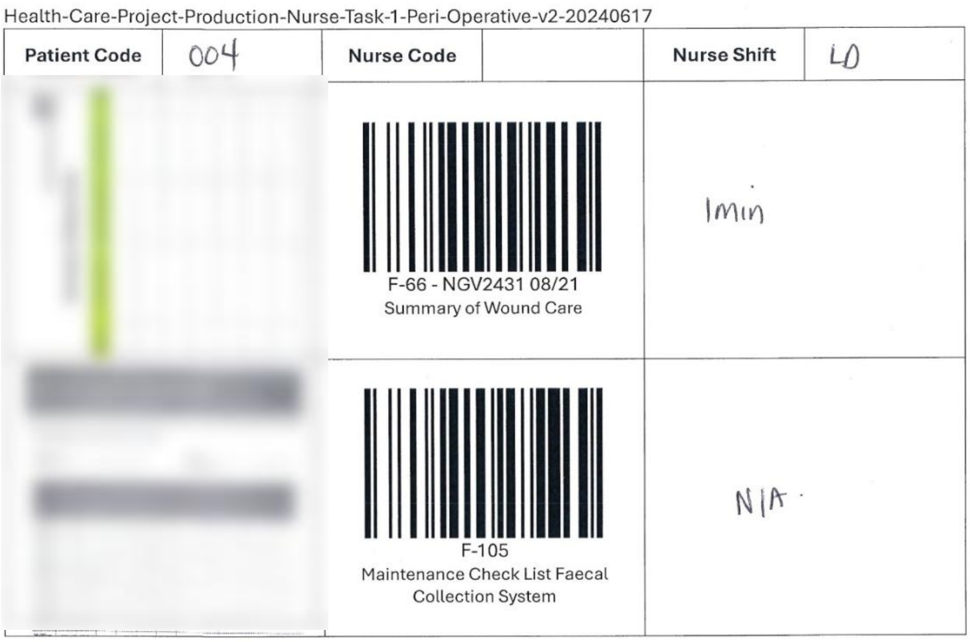

Health-Care-Project-Production-Nurse-Task-1-Peri-Operative-v2-20240617

Patient Code	004	Nurse Code		Nurse Shift	LD
		F-66 - NGV2431 08/21<br>Summary of Wound Care		1min	
		F-105<br>Maintenance Check List Faecal Collection System		N/A	

Page - 2
File - https://d.docs.live.net/f312ce15ac7eb348/Desktop/Healthcare-Project/Production/Health-Care-Project-Production-Nurse-Task-1-Peri-Operative-v2-20240617.docx

Supplementary Figure 2: Result page – 2 for peri-operative discrete event.

This peri-operative discrete event was recorded using offline method by nursing staff. The demographic data was excluded in during the data collection.

The thumbnails of observation forms used in this figure were blurred for privacy reasons.

Supplementary Figure 3: Result Page 3

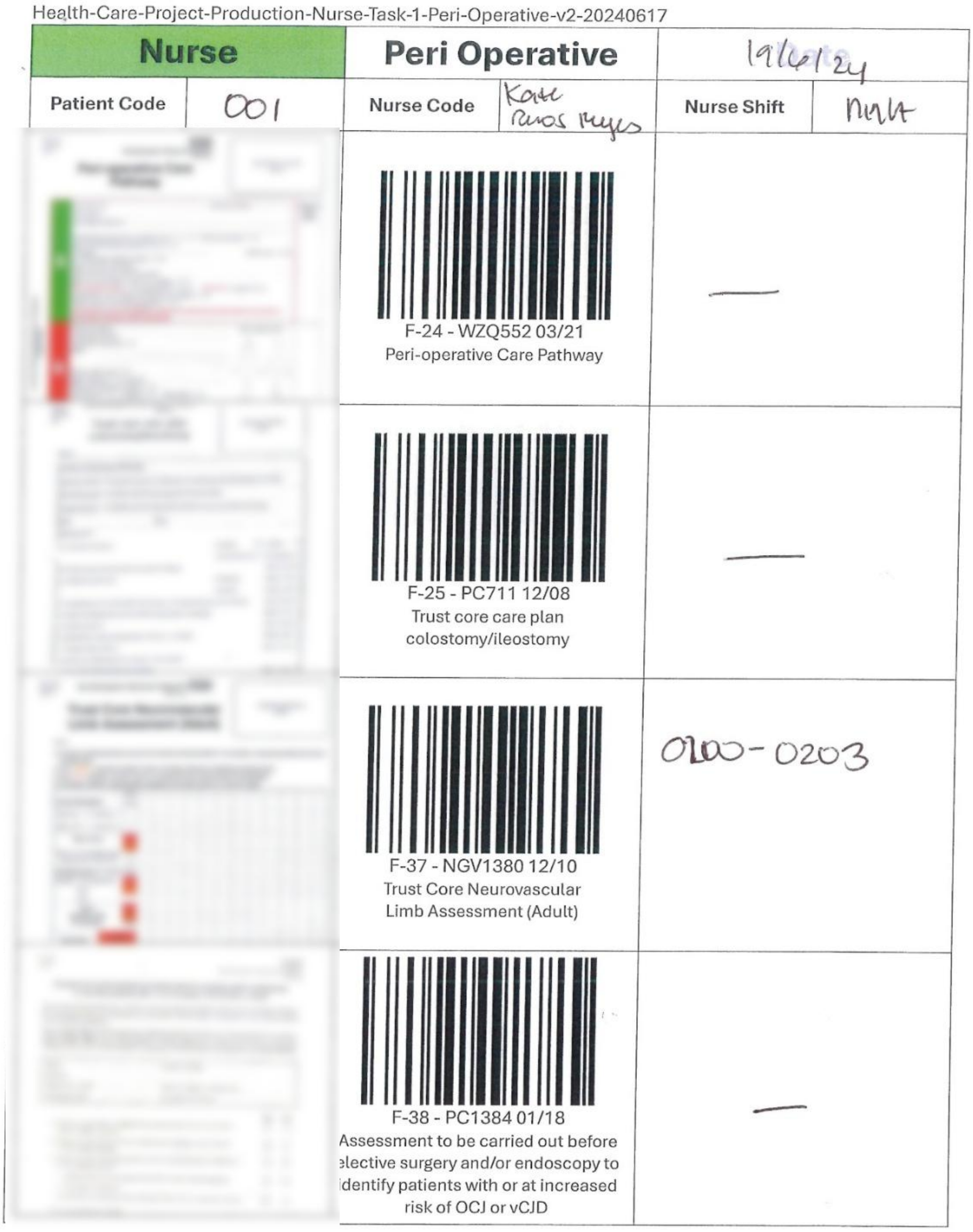

Health-Care-Project-Production-Nurse-Task-1-Peri-Operative-v2-20240617

Nurse		Peri Operative		19/6/24	
Patient Code	001	Nurse Code	[illegible]	Nurse Shift	night
[illegible]		F-24 - WZQ552 03/21 Peri-operative Care Pathway		—	
[illegible]		F-25 - PC711 12/08 Trust core care plan colostomy/ileostomy		—	
[illegible]		F-37 - NGV1380 12/10 Trust Core Neurovascular Limb Assessment (Adult)		0200-0203	
[illegible]		F-38 - PC1384 01/18 Assessment to be carried out before elective surgery and/or endoscopy to identify patients with or at increased risk of OCJ or vCJD		—	

Page - 1
File - https://d.docs.live.net/f312ce15ac7eb348/Desktop/Healthcare-Project/Production/Health-Care-Project-Production-Nurse-Task-1-Peri-Operative-v2-20240617.docx

Supplementary Figure 3: Result page – 3 for peri-operative discrete event.

This peri-operative discrete event was recorded using offline method by nursing staff. The demographic data was excluded in during the data collection.

The thumbnails of observation forms used in this figure were blurred for privacy reasons.

Supplementary Figure 4: Result Page 4

Health-Care-Project-Production-Nurse-Task-1-Peri-Operative-v2-20240617

Patient Code		Nurse Code		Nurse Shift	
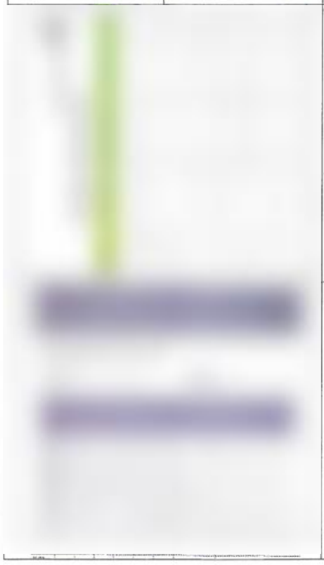		F-66 - NGV2431 08/21<br>Summary of Wound Care		—	
		F-105<br>Maintenance Check List Faecal Collection System		—	

Page - 2
File - https://d.docs.live.net/f312ce15ac7eb348/Desktop/Healthcare-Project/Production/Health-Care-Project-Production-Nurse-Task-1-Peri-Operative-v2-20240617.docx

Supplementary Figure 4: Result page – 4 for peri-operative discrete event.

No peri-operative discrete events were recorded using offline method by nursing staff. The demographic data was excluded in during the data collection.

The thumbnails of observation forms used in this figure were blurred for privacy reasons.

Supplementary Figure 5: Result Page 5

Health-Care-Project-Production-Nurse-Task-1-Peri-Operative-v2-20240617

Nurse		Peri Operative		20/6/24	
Patient Code		Nurse Code		Nurse Shift	LD
		F-24 - WZQ552 03/21<br>Peri-operative Care Pathway		N/A	
		F-25 - PC711 12/08<br>Trust core care plan colostomy/ileostomy		N/A	
		F-37 - NGV1380 12/10<br>Trust Core Neurovascular Limb Assessment (Adult)		N/A	
		F-38 - PC1384 01/18<br>Assessment to be carried out before elective surgery and/or endoscopy to identify patients with or at increased risk of OCJ or vCJD		N/A	

Page - 1
File - https://d.docs.live.net/f312ce15ac7eb348/Desktop/Healthcare-Project/Production/Health-Care-Project-Production-Nurse-Task-1-Peri-Operative-v2-20240617.docx

Supplementary Figure 5: Result page – 5 for peri-operative discrete event.

No peri-operative discrete events were recorded using offline method by nursing staff. The demographic data was excluded in during the data collection.

The thumbnails of observation forms used in this figure were blurred for privacy reasons.

Supplementary Figure 6: Result Page 6

Health-Care-Project-Production-Nurse-Task-1-Peri-Operative-v2-20240617

Patient Code		Nurse Code		Nurse Shift	
		F-66 - NGV2431 08/21<br>Summary of Wound Care		N/A	
		F-105<br>Maintenance Check List Faecal Collection System		N/A	

Page - 2
File - https://d.docs.live.net/f312ce15ac7eb348/Desktop/Healthcare-Project/Production/Health-Care-Project-Production-Nurse-Task-1-Peri-Operative-v2-20240617.docx

Supplementary Figure 6: Result page – 6 for peri-operative discrete event.

No peri-operative discrete events were recorded using offline method by nursing staff. The demographic data was excluded in during the data collection.

The thumbnails of observation forms used in this figure were blurred for privacy reasons.

Supplementary Figure 7: Result Page 7

Health-Care-Project-Production-Nurse-Task-2-Medical-Diseases-v2-20240617

Nurse		Medical Diseases	20/6/24	
Patient Code		Nurse Code	Nurse Shift	LD
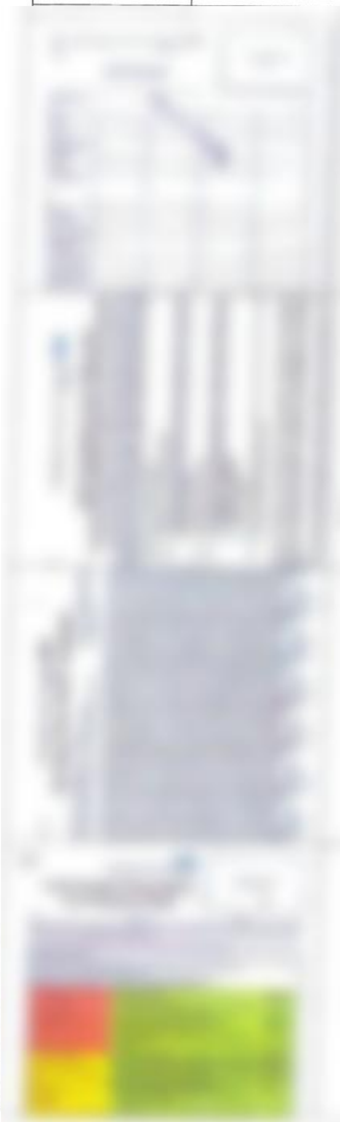		F-20 - NGV285 03/03 Fit Chart	N/A	
		F-22 - NGV312 02/16 Adult neurological observation chart incorporating pupillary response and limb movements	N/A	
		F-28 - NGV889 11/04 EU Peak Flow Chart - Inpatient	N/A	
		F-39 - NGV1424 03/23 Adult Insulin Prescription and Diabetes Chart	N/A	

Page - 1
File - https://d.docs.live.net/f312ce15ac7eb348/Desktop/Healthcare-Project/Production/Health-Care-Project-Production-Nurse-Task-2-Medical-Diseases-v2-20240617.docx

Supplementary Figure 7: Result page – 7 for medical diseases discrete event.

No medical disease discrete events were recorded using offline method by nursing staff. The demographic data was excluded in during the data collection.

The thumbnails of observation forms used in this figure were blurred for privacy reasons.

Supplementary Figure 9: Result Page 8

Health-Care-Project-Production-Nurse-Task-2-Medical-Diseases-v2-20240617

Patient Code		Nurse Code		Nurse Shift	20/6/24
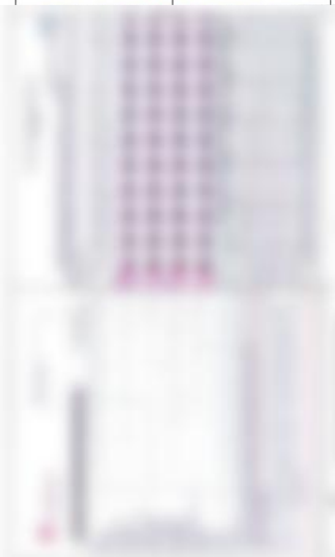		F-45 - NGV1598 05/18<br>Diabetes sugar monitoring chart for patients not on insulin		N/A	
		F-100<br>Glasgow Modified Alcohol Withdrawal Scale (GMAWS)		N/A	

Page - 2
File - https://d.docs.live.net/f312ce15ac7eb348/Desktop/Healthcare-Project/Production/Health-Care-Project-Production-Nurse-Task-2-Medical-Diseases-v2-20240617.docx

Supplementary Figure 7: Result page – 7 for medical diseases discrete event.

No medical disease discrete events were recorded using offline method by nursing staff. The demographic data was excluded in during the data collection.

The thumbnails of observation forms used in this figure were blurred for privacy reasons.

Supplementary Figure 9: Result Page 9

Health-Care-Project-Production-Nurse-Task-2-Medical-Diseases-v2-20240617

Nurse		Medical Diseases		20/6/24 [illegible]te	
Patient Code	004	Nurse Code		Nurse Shift	LD.

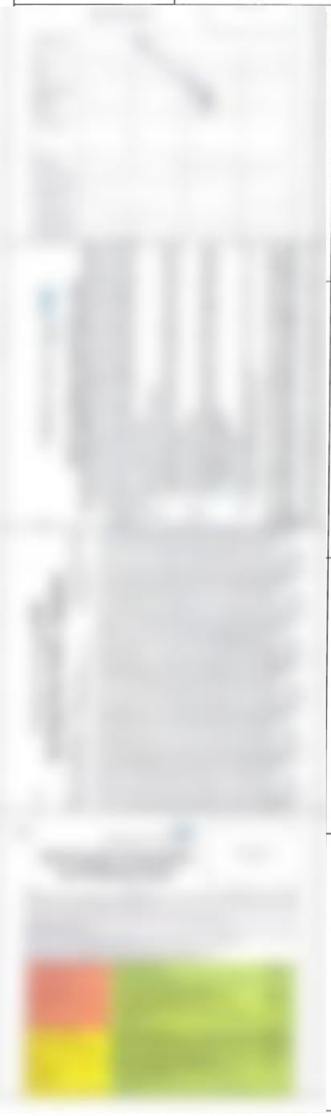

F-20 - NGV285 03/03<br>Fit Chart	N/A
F-22 - NGV312 02/16<br>Adult neurological observation chart incorporating pupillary response and limb movements	N/A.
F-28 - NGV889 11/04<br>EU Peak Flow Chart - Inpatient	N/A
F-39 - NGV1424 03/23<br>Adult Insulin Prescription and Diabetes Chart	N/A

Page - 1
File - https://d.docs.live.net/f312ce15ac7eb348/Desktop/Healthcare-Project/Production/Health-Care-Project-Production-Nurse-Task-2-Medical-Diseases-v2-20240617.docx

Supplementary Figure 7: Result page – 7 for medical diseases discrete event.

No medical disease discrete events were recorded using offline method by nursing staff. The demographic data was excluded in during the data collection.

The thumbnails of observation forms used in this figure were blurred for privacy reasons.

Supplementary Figure 10: Result Page 10

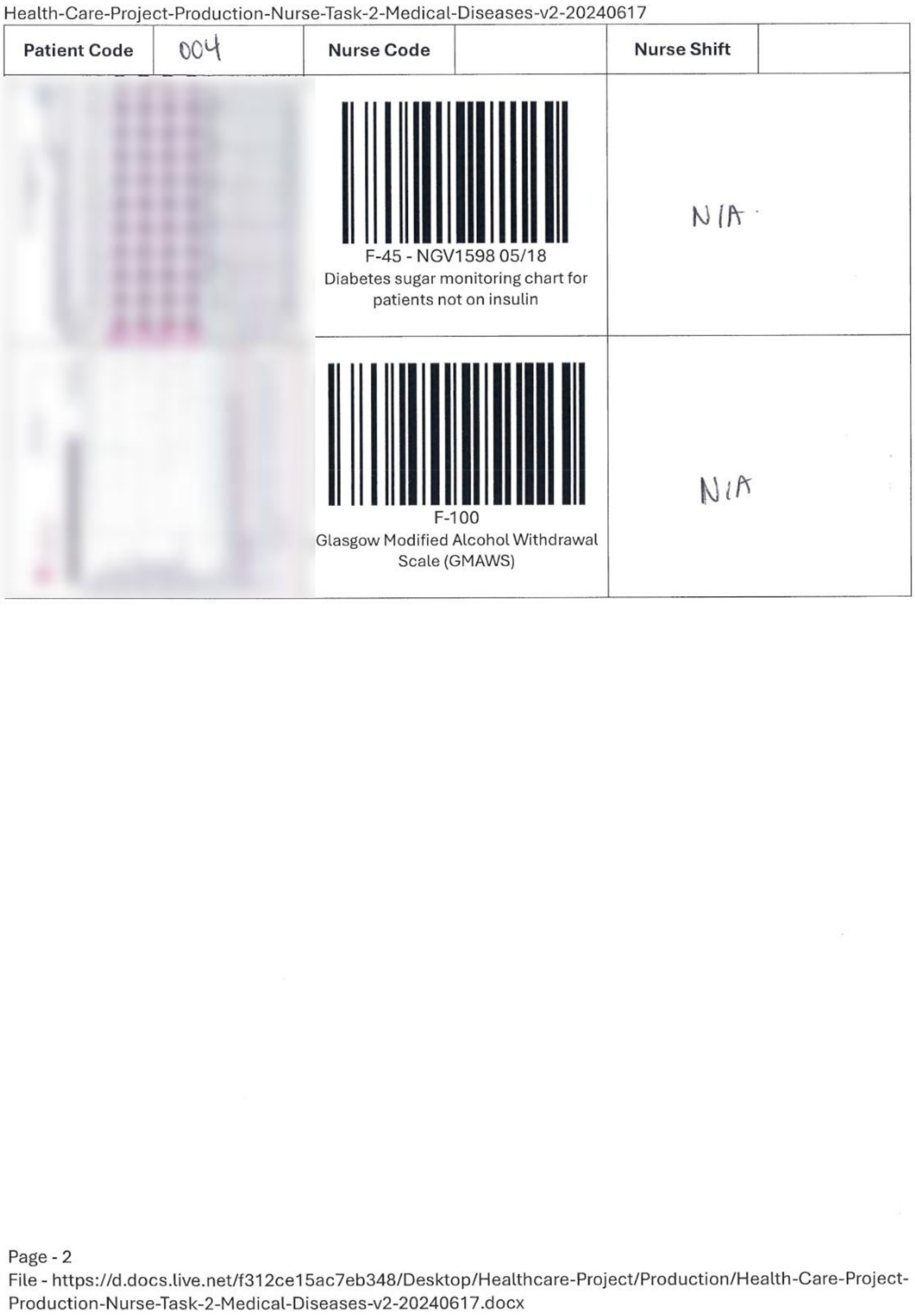

Health-Care-Project-Production-Nurse-Task-2-Medical-Diseases-v2-20240617

Patient Code	004	Nurse Code		Nurse Shift	
		F-45 - NGV1598 05/18<br>Diabetes sugar monitoring chart for patients not on insulin		N/A	
		F-100<br>Glasgow Modified Alcohol Withdrawal Scale (GMAWS)		N/A	

Page - 2
File - https://d.docs.live.net/f312ce15ac7eb348/Desktop/Healthcare-Project/Production/Health-Care-Project-Production-Nurse-Task-2-Medical-Diseases-v2-20240617.docx

Supplementary Figure 7: Result page – 7 for medical diseases discrete event.

No medical disease discrete events were recorded using offline method by nursing staff. The demographic data was excluded in during the data collection.

The thumbnails of observation forms used in this figure were blurred for privacy reasons.

Supplementary Figure 11: Result Page 11

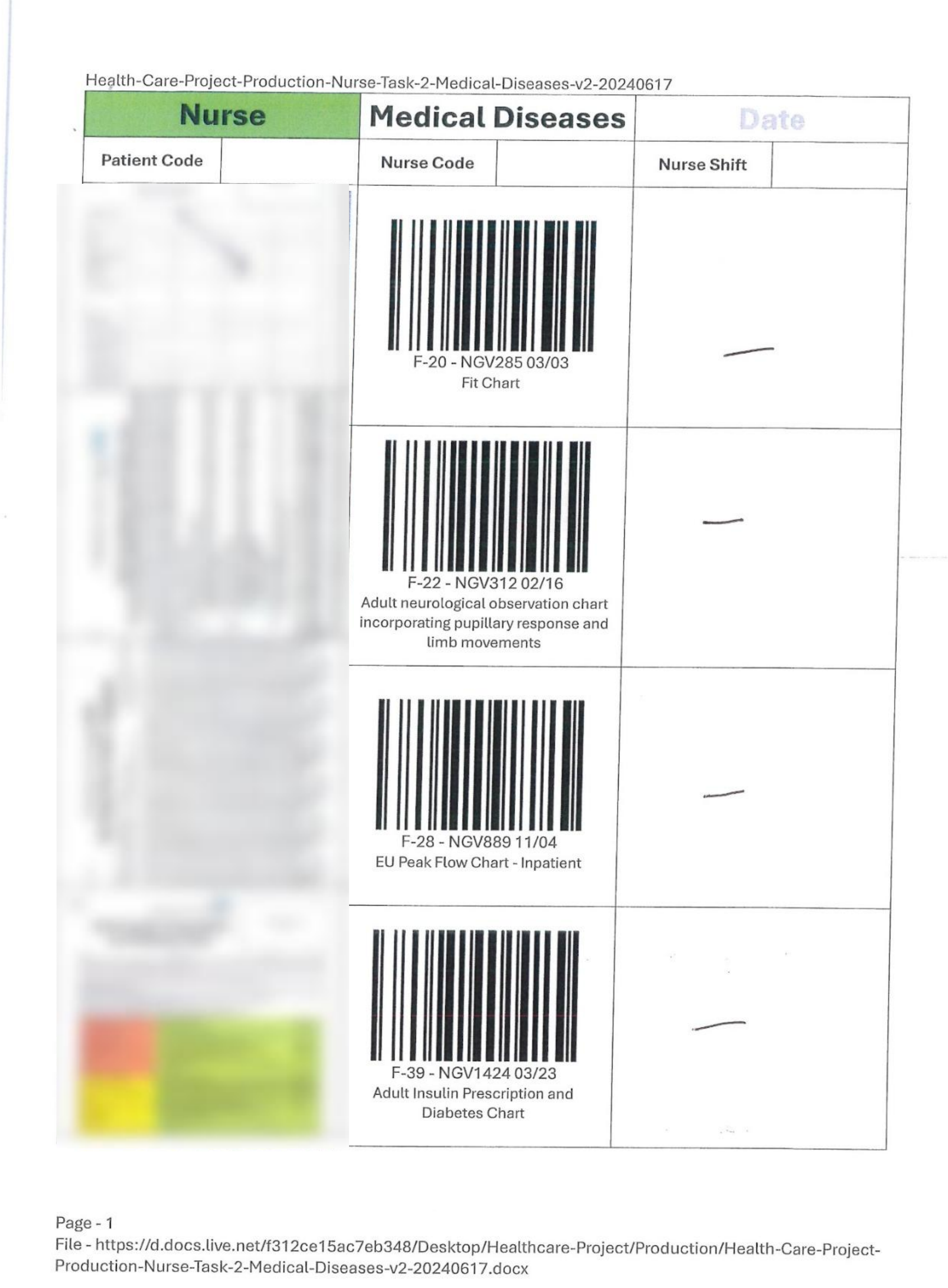

Health-Care-Project-Production-Nurse-Task-2-Medical-Diseases-v2-20240617

Nurse		Medical Diseases		Date	
Patient Code		Nurse Code		Nurse Shift	

	F-20 - NGV285 03/03<br>Fit Chart	—
	F-22 - NGV312 02/16<br>Adult neurological observation chart incorporating pupillary response and limb movements	—
	F-28 - NGV889 11/04<br>EU Peak Flow Chart - Inpatient	—
	F-39 - NGV1424 03/23<br>Adult Insulin Prescription and Diabetes Chart	—

Page - 1
File - https://d.docs.live.net/f312ce15ac7eb348/Desktop/Healthcare-Project/Production/Health-Care-Project-Production-Nurse-Task-2-Medical-Diseases-v2-20240617.docx

Supplementary Figure 7: Result page – 7 for medical diseases discrete event.

No medical disease discrete events were recorded using offline method by nursing staff. The demographic data was excluded in during the data collection.

The thumbnails of observation forms used in this figure were blurred for privacy reasons.

Supplementary Figure 12: Result Page 12

Health-Care-Project-Production-Nurse-Task-2-Medical-Diseases-v2-20240617

Patient Code		Nurse Code		Nurse Shift	
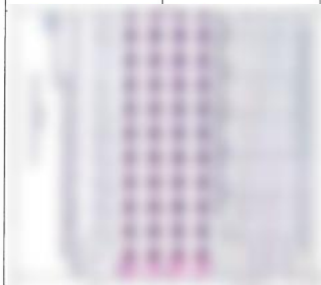		F-45 - NGV1598 05/18<br>Diabetes sugar monitoring chart for patients not on insulin		—	
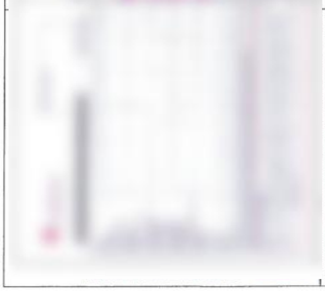		F-100<br>Glasgow Modified Alcohol Withdrawal Scale (GMAWS)		—	

Page - 2
File - https://d.docs.live.net/f312ce15ac7eb348/Desktop/Healthcare-Project/Production/Health-Care-Project-Production-Nurse-Task-2-Medical-Diseases-v2-20240617.docx

Supplementary Figure 7: Result page – 7 for medical diseases discrete event.

No medical disease discrete events were recorded using offline method by nursing staff. The demographic data was excluded in during the data collection.

The thumbnails of observation forms used in this figure were blurred for privacy reasons.

Supplementary Figure 13: Result Page 13

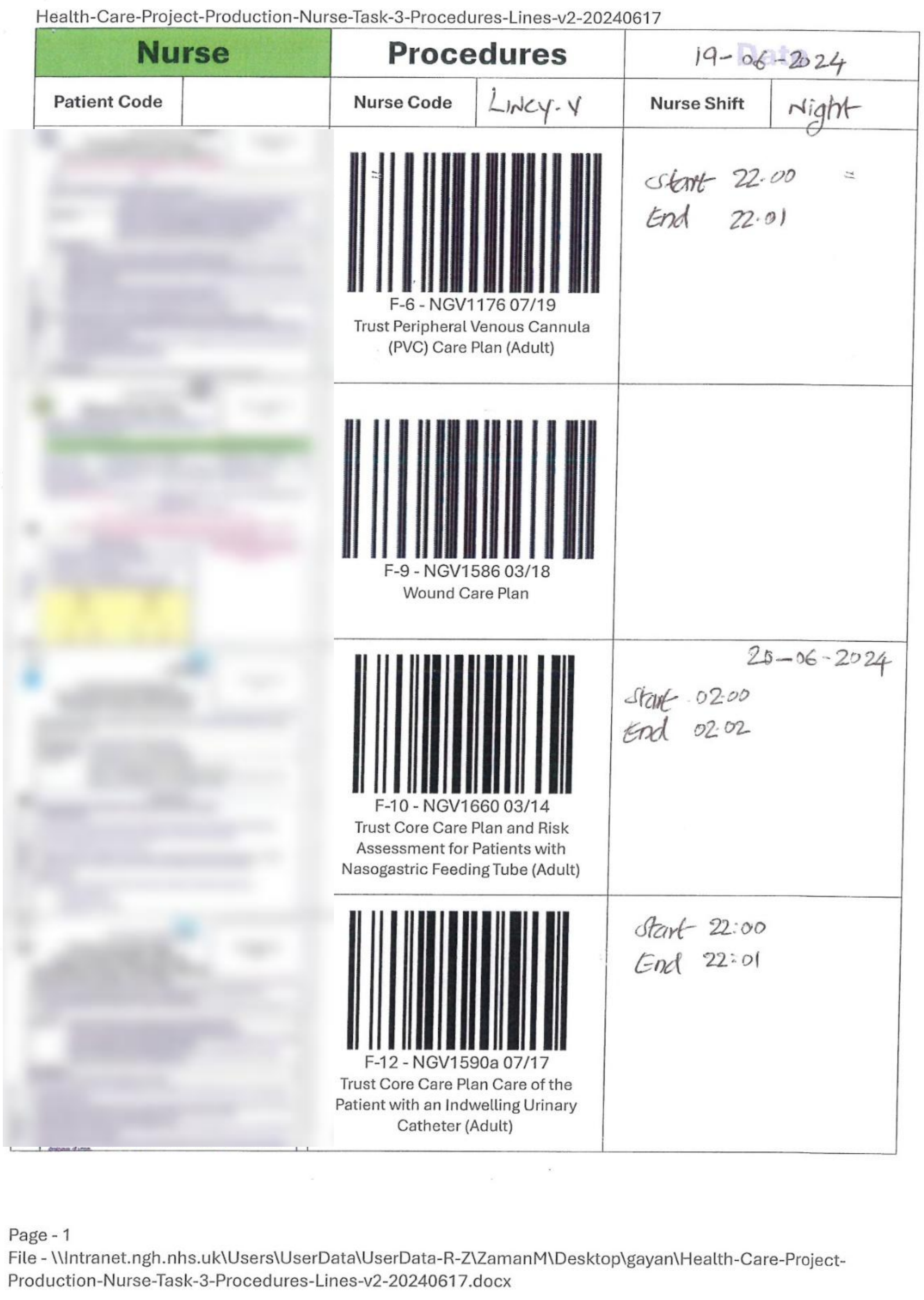

Health-Care-Project-Production-Nurse-Task-3-Procedures-Lines-v2-20240617

Nurse		Procedures		Date 19-06-2024	
Patient Code		Nurse Code	Lincy. V	Nurse Shift	Night
		F-6 - NGV1176 07/19<br>Trust Peripheral Venous Cannula (PVC) Care Plan (Adult)		Start 22.00<br>End 22.01	
		F-9 - NGV1586 03/18<br>Wound Care Plan			
		F-10 - NGV1660 03/14<br>Trust Core Care Plan and Risk Assessment for Patients with Nasogastric Feeding Tube (Adult)		20-06-2024<br>Start 02:00<br>End 02:02	
		F-12 - NGV1590a 07/17<br>Trust Core Care Plan Care of the Patient with an Indwelling Urinary Catheter (Adult)		Start 22:00<br>End 22:01	

Page - 1
File - \\Intranet.ngh.nhs.uk\Users\UserData\UserData-R-Z\ZamanM\Desktop\gayan\Health-Care-Project-Production-Nurse-Task-3-Procedures-Lines-v2-20240617.docx

Supplementary Figure 7: Result page – 7 for procedure-lines discrete event.

These procedure-lines discrete event were recorded using offline method by nursing staff. The demographic data was excluded in during the data collection.

The thumbnails of observation forms used in this figure were blurred for privacy reasons.

Supplementary Figure 14: Result Page 14

Health-Care-Project-Production-Nurse-Task-3-Procedures-Lines-v2-20240617

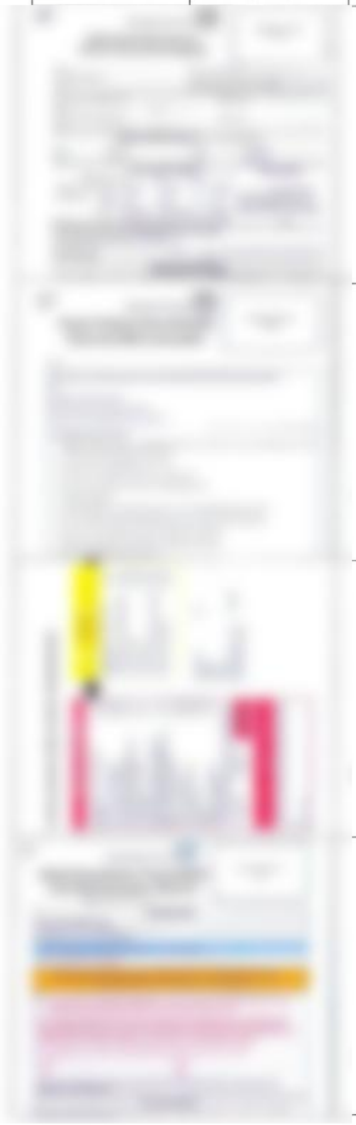

Patient Code	002	Nurse Code	Linq.V	Nurse Shift	Night
		F-16 - NGV090 08/22 Adult Acute Pain Service Patient Controlled Analgesia			
		F-32 - NGV1239 05/18 Trust Critical Care Arterial Cannula (AC) Care Plan		Start : 22:30 End : 22:31	
		F-46 - NGV1644 02/16 Trust Core Care Plan: Tracheostomy (Adult)			
		F-53 - NGV1771 09/20 Adult Transfusion Prescription and Administration Record			

Page - 2
File - \\Intranet.ngh.nhs.uk\Users\UserData\UserData-R-Z\ZamanM\Desktop\gayan\Health-Care-Project-Production-Nurse-Task-3-Procedures-Lines-v2-20240617.docx

Supplementary Figure 7: Result page – 7 for procedure-lines discrete event.

These procedure-lines discrete event were recorded using offline method by nursing staff. The demographic data was excluded in during the data collection.

The thumbnails of observation forms used in this figure were blurred for privacy reasons.

Supplementary Figure 15: Result Page 15

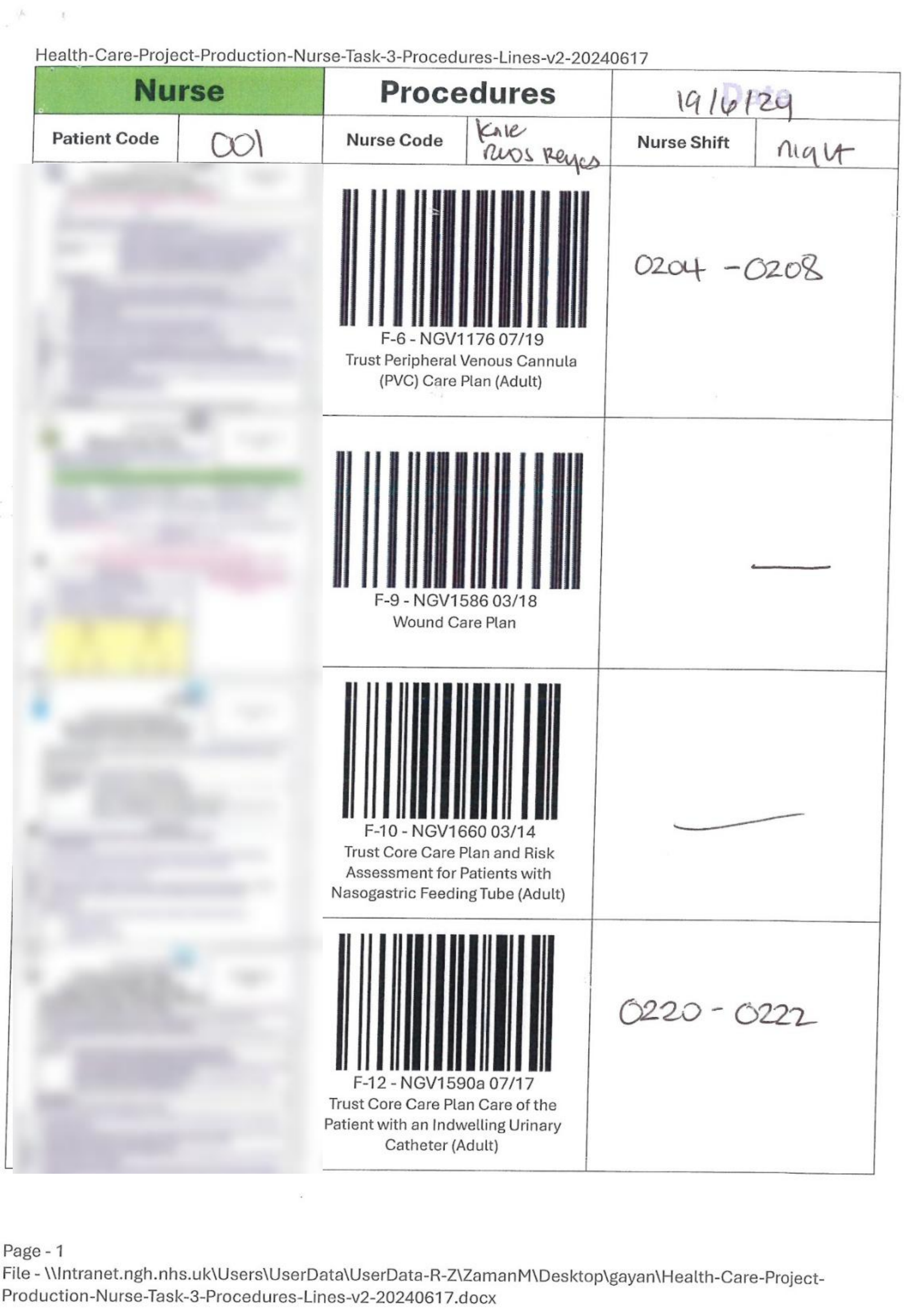

Health-Care-Project-Production-Nurse-Task-3-Procedures-Lines-v2-20240617

Nurse		Procedures		19/6/24	
Patient Code	001	Nurse Code	Kale Ruos Reyes	Nurse Shift	night
		F-6 - NGV1176 07/19 Trust Peripheral Venous Cannula (PVC) Care Plan (Adult)		0204 - 0208	
		F-9 - NGV1586 03/18 Wound Care Plan		—	
		F-10 - NGV1660 03/14 Trust Core Care Plan and Risk Assessment for Patients with Nasogastric Feeding Tube (Adult)		—	
		F-12 - NGV1590a 07/17 Trust Core Care Plan Care of the Patient with an Indwelling Urinary Catheter (Adult)		0220 - 0222	

Page - 1
File - \\Intranet.ngh.nhs.uk\Users\UserData\UserData-R-Z\ZamanM\Desktop\gayan\Health-Care-Project-Production-Nurse-Task-3-Procedures-Lines-v2-20240617.docx

Supplementary Figure 7: Result page – 7 for procedure-lines discrete event.

These procedure-lines discrete events were recorded using offline method by nursing staff. The demographic data was excluded in during the data collection.

The thumbnails of observation forms used in this figure were blurred for privacy reasons.

Supplementary Figure 16: Result Page 16

Health-Care-Project-Production-Nurse-Task-3-Procedures-Lines-v2-20240617

Patient Code		Nurse Code		Nurse Shift	
		F-16 - NGV090 08/22<br>Adult Acute Pain Service Patient Controlled Analgesia		—	
		F-32 - NGV1239 05/18<br>Trust Critical Care Arterial Cannula (AC) Care Plan		0200-0203	
		F-46 - NGV1644 02/16<br>Trust Core Care Plan: Tracheostomy (Adult)		—	
		F-53 - NGV1771 09/20<br>Adult Transfusion Prescription and Administration Record		—	

Page - 2
File - \\Intranet.ngh.nhs.uk\Users\UserData\UserData-R-Z\ZamanM\Desktop\gayan\Health-Care-Project-Production-Nurse-Task-3-Procedures-Lines-v2-20240617.docx

Supplementary Figure 7: Result page – 7 for procedure-lines discrete event.

This procedure-lines discrete event was recorded using offline method by nursing staff. The demographic data was excluded in during the data collection.

The thumbnails of observation forms used in this figure were blurred for privacy reasons.

Supplementary Figure 17: Result Page 17

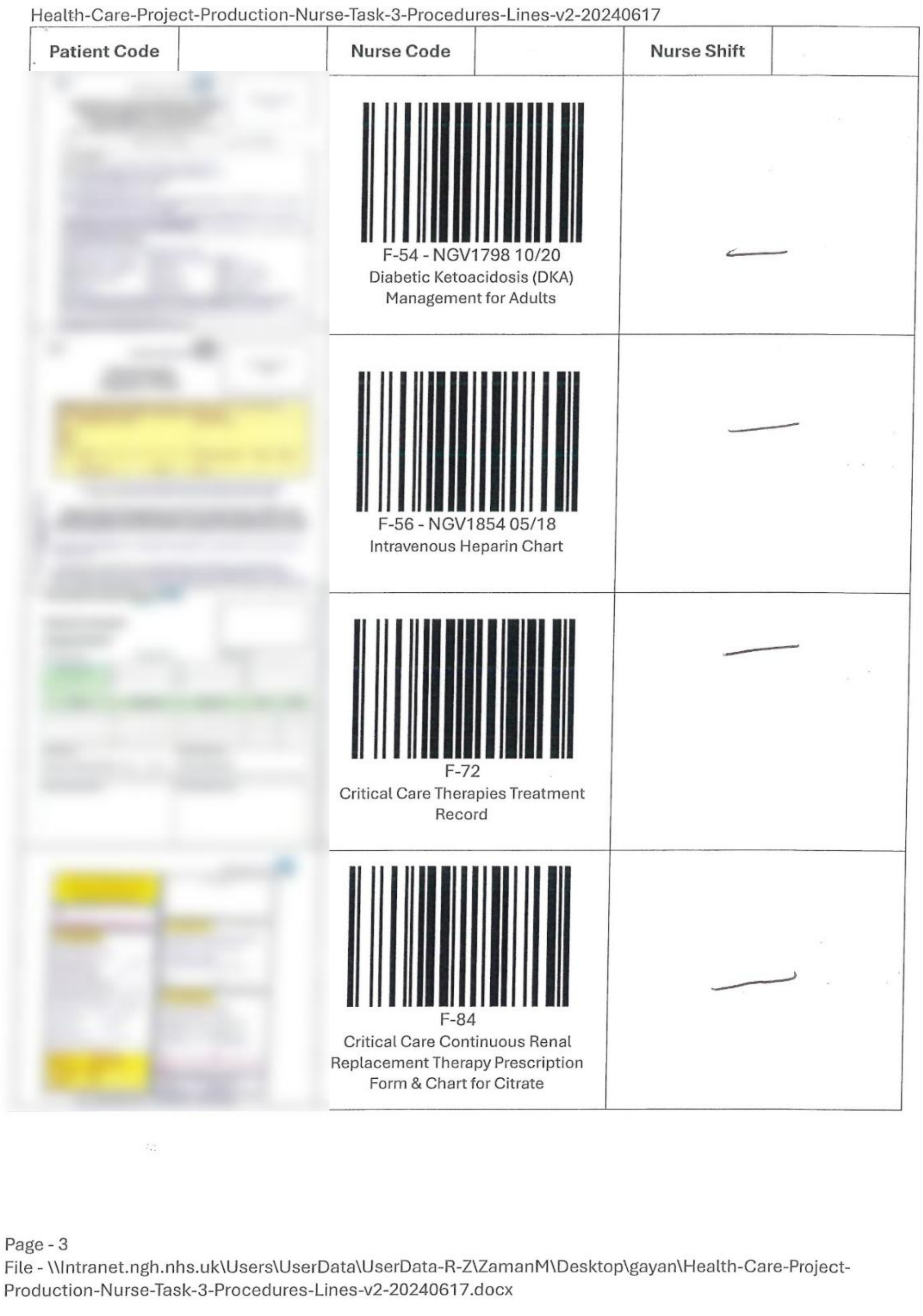

Health-Care-Project-Production-Nurse-Task-3-Procedures-Lines-v2-20240617

Patient Code		Nurse Code		Nurse Shift	
		F-54 - NGV1798 10/20<br>Diabetic Ketoacidosis (DKA) Management for Adults		—	
		F-56 - NGV1854 05/18<br>Intravenous Heparin Chart		—	
		F-72<br>Critical Care Therapies Treatment Record		—	
		F-84<br>Critical Care Continuous Renal Replacement Therapy Prescription Form & Chart for Citrate		—	

Page - 3
File - \\Intranet.ngh.nhs.uk\Users\UserData\UserData-R-Z\ZamanM\Desktop\gayan\Health-Care-Project-Production-Nurse-Task-3-Procedures-Lines-v2-20240617.docx

Supplementary Figure 17: Result page – 17 for procedure-lines discrete event.

No procedure-lines discrete events were recorded using offline method by nursing staff. The demographic data was excluded in during the data collection.

The thumbnails of observation forms used in this figure were blurred for privacy reasons.

Supplementary Figure 18: Result Page 18

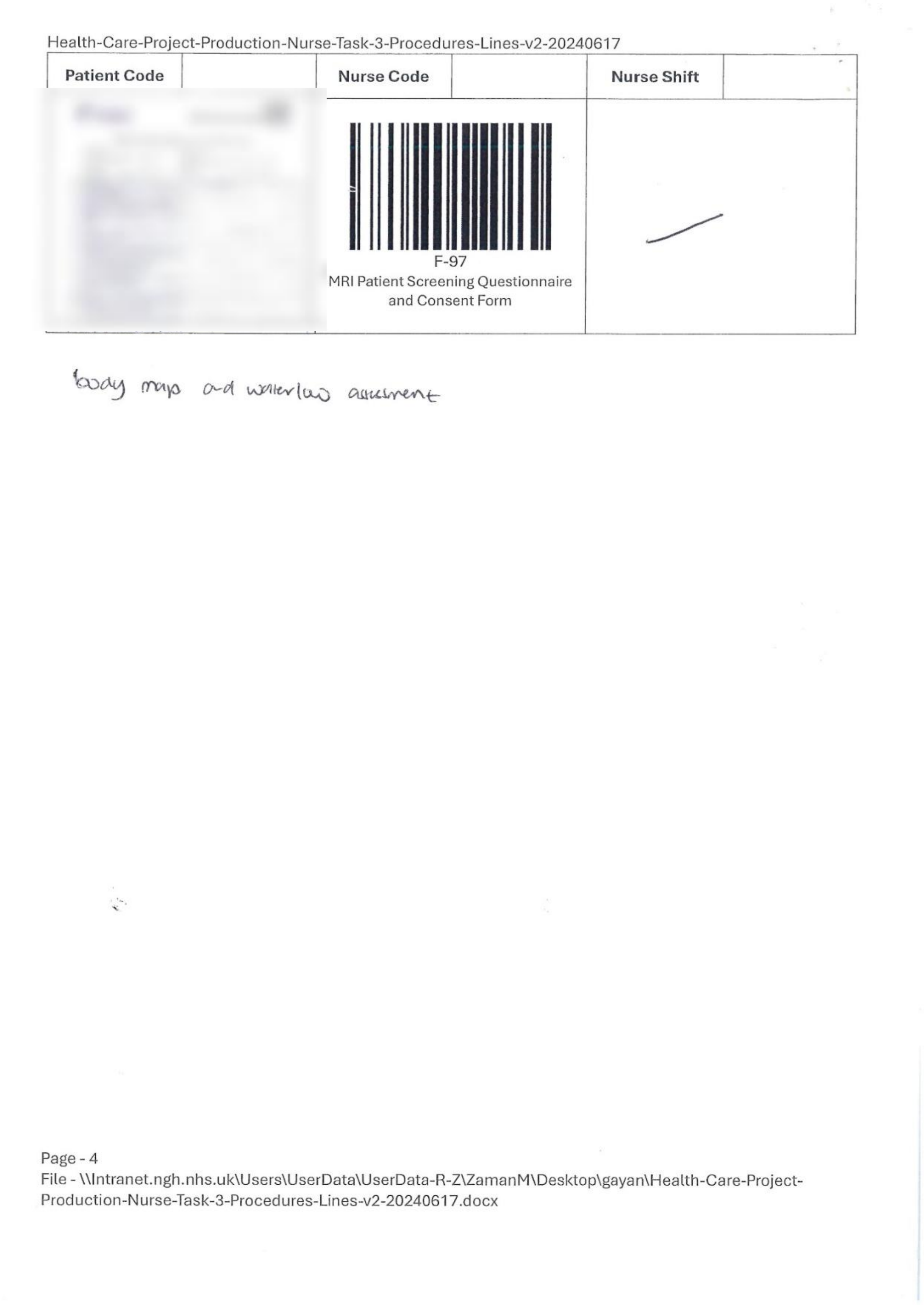

Health-Care-Project-Production-Nurse-Task-3-Procedures-Lines-v2-20240617

Patient Code		Nurse Code		Nurse Shift	
		F-97<br>MRI Patient Screening Questionnaire and Consent Form			

body maps and waterlow assessment

Page - 4
File - \\Intranet.ngh.nhs.uk\Users\UserData\UserData-R-Z\ZamanM\Desktop\gayan\Health-Care-Project-Production-Nurse-Task-3-Procedures-Lines-v2-20240617.docx

Supplementary Figure 18: Result page – 18 for procedure-lines discrete event.

No procedure-lines discrete event was recorded using offline method by nursing staff. The demographic data was excluded in during the data collection.

The thumbnails of observation forms used in this figure were blurred for privacy reasons.

Supplementary Figure 19: Result Page 19

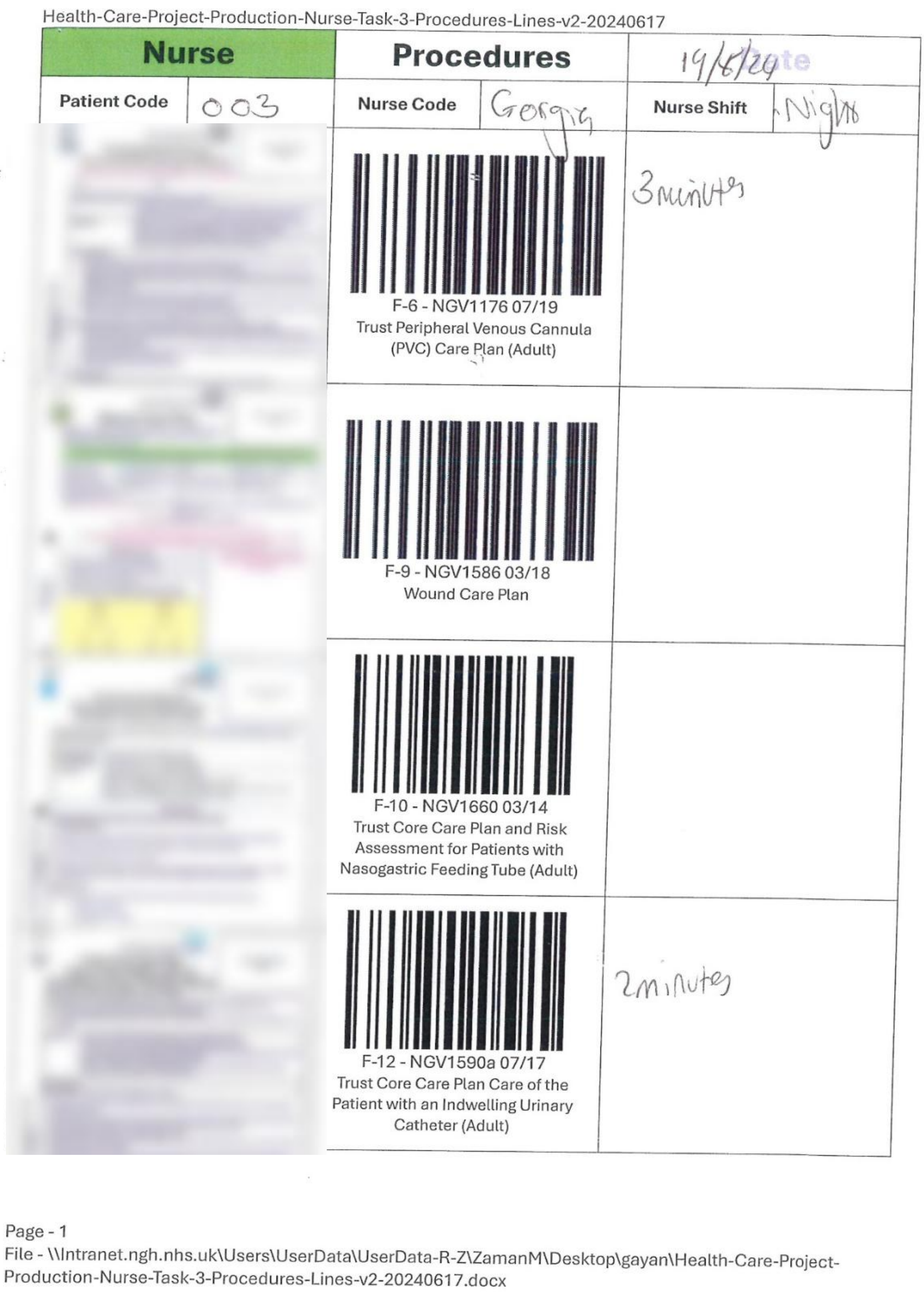

Health-Care-Project-Production-Nurse-Task-3-Procedures-Lines-v2-20240617

Nurse		Procedures		19/6/24 Date	
Patient Code	003	Nurse Code	Georgia	Nurse Shift	Night
		F-6 - NGV1176 07/19<br>Trust Peripheral Venous Cannula (PVC) Care Plan (Adult)		3 minutes	
		F-9 - NGV1586 03/18<br>Wound Care Plan			
		F-10 - NGV1660 03/14<br>Trust Core Care Plan and Risk Assessment for Patients with Nasogastric Feeding Tube (Adult)			
		F-12 - NGV1590a 07/17<br>Trust Core Care Plan Care of the Patient with an Indwelling Urinary Catheter (Adult)		2 minutes	

Page - 1
File - \\Intranet.ngh.nhs.uk\Users\UserData\UserData-R-Z\ZamanM\Desktop\gayan\Health-Care-Project-Production-Nurse-Task-3-Procedures-Lines-v2-20240617.docx

Supplementary Figure 19: Result page – 19 for procedure-lines discrete event.

These procedure-lines discrete event were recorded using offline method by nursing staff. The demographic data was excluded in during the data collection.

The thumbnails of observation forms used in this figure were blurred for privacy reasons.

Supplementary Figure 20: Result Page 20

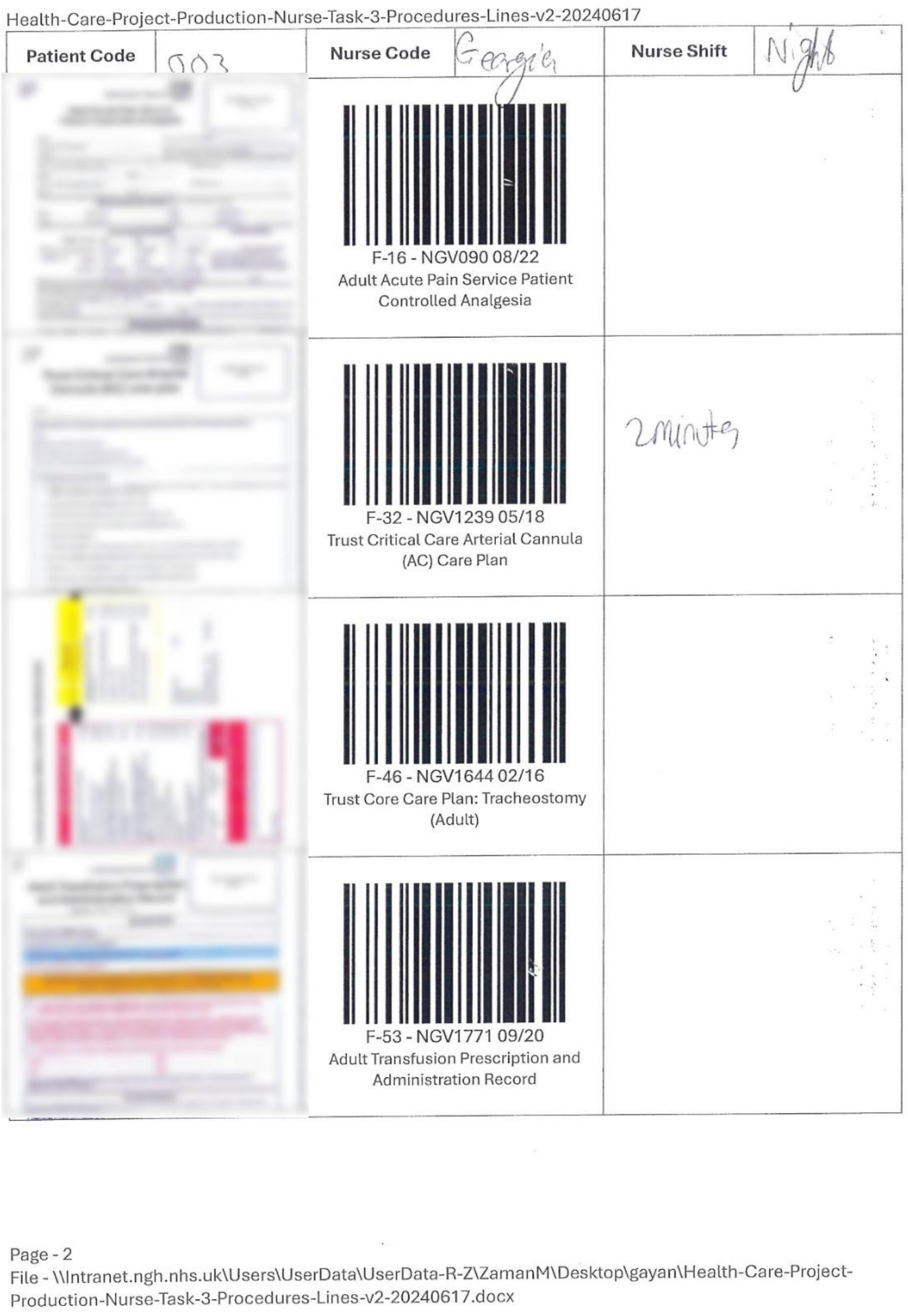

Health-Care-Project-Production-Nurse-Task-3-Procedures-Lines-v2-20240617

Patient Code	003	Nurse Code	Georgie	Nurse Shift	Night
		F-16 - NGV090 08/22 Adult Acute Pain Service Patient Controlled Analgesia			
		F-32 - NGV1239 05/18 Trust Critical Care Arterial Cannula (AC) Care Plan		2minutes	
		F-46 - NGV1644 02/16 Trust Core Care Plan: Tracheostomy (Adult)			
		F-53 - NGV1771 09/20 Adult Transfusion Prescription and Administration Record			

Page - 2

File - \\Intranet.ngh.nhs.uk\Users\UserData\UserData-R-Z\ZamanM\Desktop\gayan\Health-Care-Project-Production-Nurse-Task-3-Procedures-Lines-v2-20240617.docx

Supplementary Figure 20: Result page – 20 for procedure-lines discrete event.

This procedure-lines discrete event was recorded using offline method by nursing staff. The demographic data was excluded in during the data collection.

The thumbnails of observation forms used in this figure were blurred for privacy reasons.

Supplementary Figure 21: Result Page 21

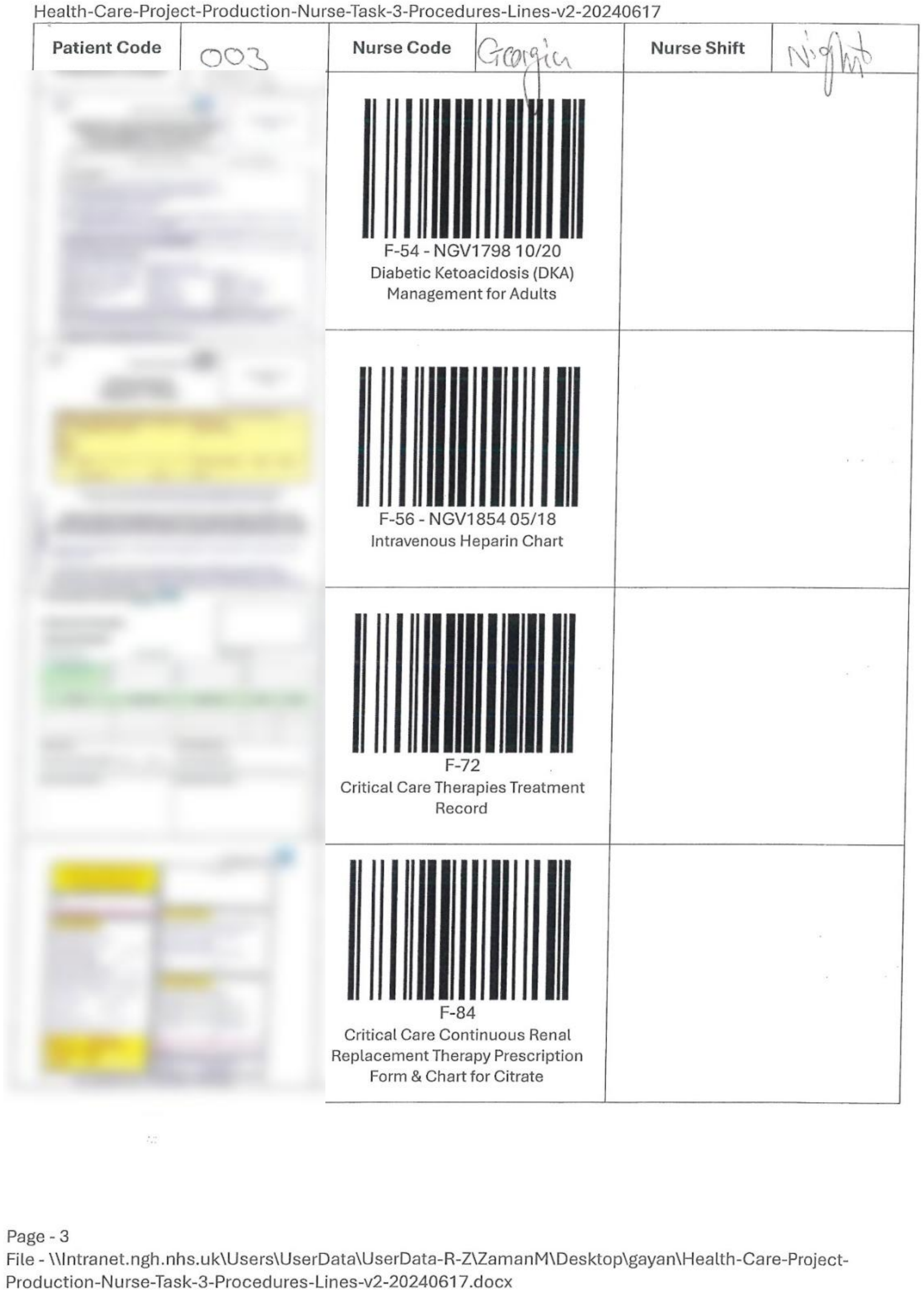

Health-Care-Project-Production-Nurse-Task-3-Procedures-Lines-v2-20240617

Patient Code	003	Nurse Code	Georgia	Nurse Shift	Night
		F-54 - NGV1798 10/20<br>Diabetic Ketoacidosis (DKA) Management for Adults			
		F-56 - NGV1854 05/18<br>Intravenous Heparin Chart			
		F-72<br>Critical Care Therapies Treatment Record			
		F-84<br>Critical Care Continuous Renal Replacement Therapy Prescription Form & Chart for Citrate			

Page - 3
File - \\Intranet.ngh.nhs.uk\Users\UserData\UserData-R-Z\ZamanM\Desktop\gayan\Health-Care-Project-Production-Nurse-Task-3-Procedures-Lines-v2-20240617.docx

Supplementary Figure 21: Result page – 21 for procedure-lines discrete event.

No procedure-lines discrete events were recorded using offline method by nursing staff. The demographic data was excluded in during the data collection.

The thumbnails of observation forms used in this figure were blurred for privacy reasons.

Supplementary Figure 22: Result Page 22

Health-Care-Project-Production-Nurse-Task-3-Procedures-Lines-v2-20240617

Patient Code		Nurse Code		Nurse Shift	
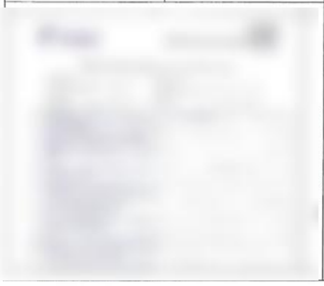		F-97 MRI Patient Screening Questionnaire and Consent Form			

Page - 4
File - \\Intranet.ngh.nhs.uk\Users\UserData\UserData-R-Z\ZamanM\Desktop\gayan\Health-Care-Project-Production-Nurse-Task-3-Procedures-Lines-v2-20240617.docx

Supplementary Figure 22: Result page – 22 for procedure-lines discrete event.

No procedure-lines discrete event was recorded using offline method by nursing staff. The demographic data was excluded in during the data collection.

The thumbnails of observation forms used in this figure were blurred for privacy reasons.

Supplementary Figure 23: Result Page 23

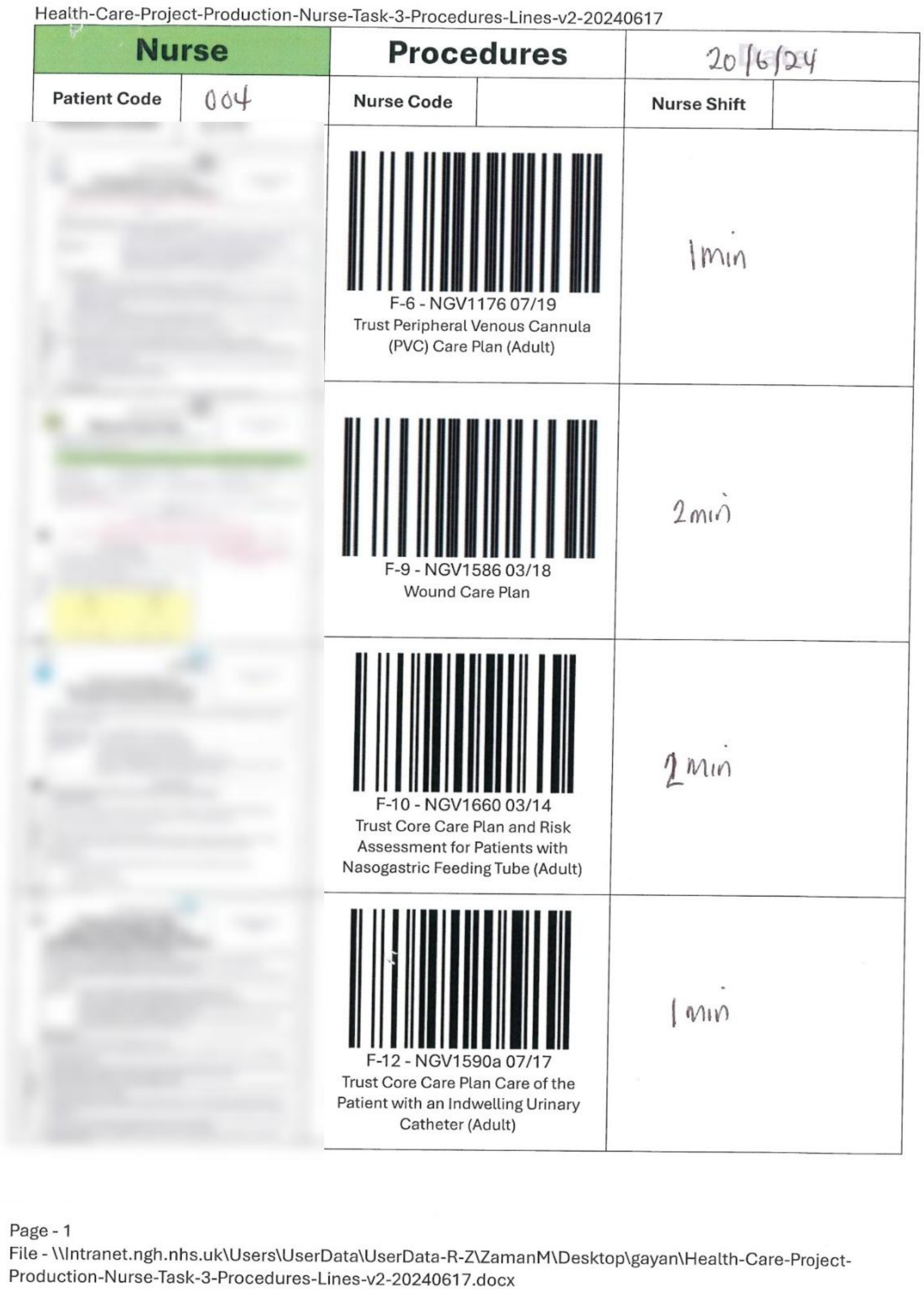

Health-Care-Project-Production-Nurse-Task-3-Procedures-Lines-v2-20240617

Nurse		Procedures		20/6/24	
Patient Code	004	Nurse Code		Nurse Shift	
		F-6 - NGV1176 07/19<br>Trust Peripheral Venous Cannula (PVC) Care Plan (Adult)		1min	
		F-9 - NGV1586 03/18<br>Wound Care Plan		2min	
		F-10 - NGV1660 03/14<br>Trust Core Care Plan and Risk Assessment for Patients with Nasogastric Feeding Tube (Adult)		2min	
		F-12 - NGV1590a 07/17<br>Trust Core Care Plan Care of the Patient with an Indwelling Urinary Catheter (Adult)		1min	

Page - 1
File - \\Intranet.ngh.nhs.uk\Users\UserData\UserData-R-Z\ZamanM\Desktop\gayan\Health-Care-Project-Production-Nurse-Task-3-Procedures-Lines-v2-20240617.docx

Supplementary Figure 23: Result page – 23 for procedure-lines discrete event.

These procedure-lines discrete events were recorded using offline method by nursing staff. The demographic data was excluded in during the data collection.

The thumbnails of observation forms used in this figure were blurred for privacy reasons.

Supplementary Figure 24: Result Page 24

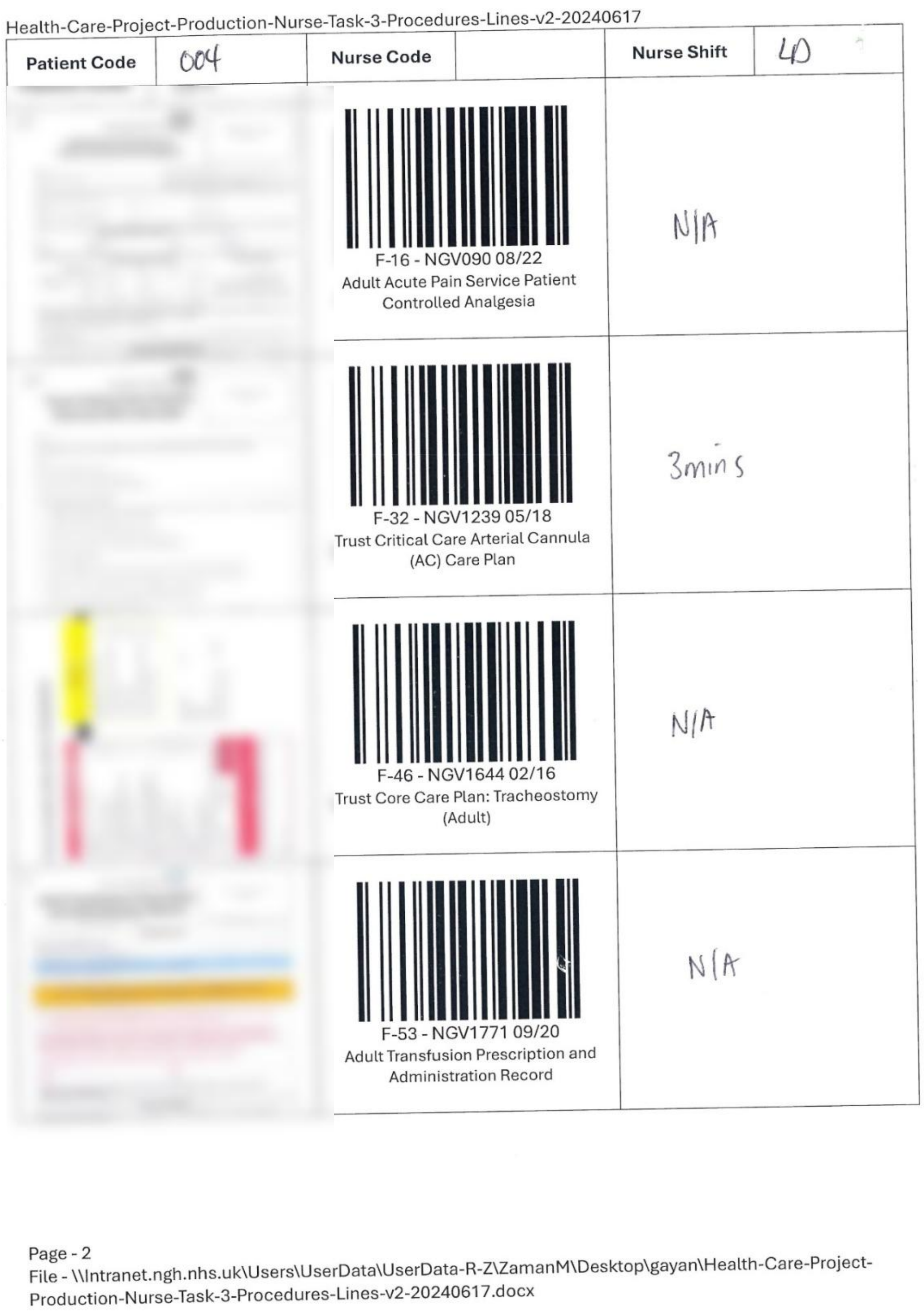

Health-Care-Project-Production-Nurse-Task-3-Procedures-Lines-v2-20240617

Patient Code	004	Nurse Code		Nurse Shift	LD

	F-16 - NGV090 08/22<br>Adult Acute Pain Service Patient Controlled Analgesia	N/A
	F-32 - NGV1239 05/18<br>Trust Critical Care Arterial Cannula (AC) Care Plan	3mins
	F-46 - NGV1644 02/16<br>Trust Core Care Plan: Tracheostomy (Adult)	N/A
	F-53 - NGV1771 09/20<br>Adult Transfusion Prescription and Administration Record	N/A

Page - 2
File - \\Intranet.ngh.nhs.uk\Users\UserData\UserData-R-Z\ZamanM\Desktop\gayan\Health-Care-Project-Production-Nurse-Task-3-Procedures-Lines-v2-20240617.docx

Supplementary Figure 24: Result page – 24 for procedure-lines discrete event.

This procedure-lines discrete event was recorded using offline method by nursing staff. The demographic data was excluded in during the data collection.

The thumbnails of observation forms used in this figure were blurred for privacy reasons.

Supplementary Figure 25: Result Page 25

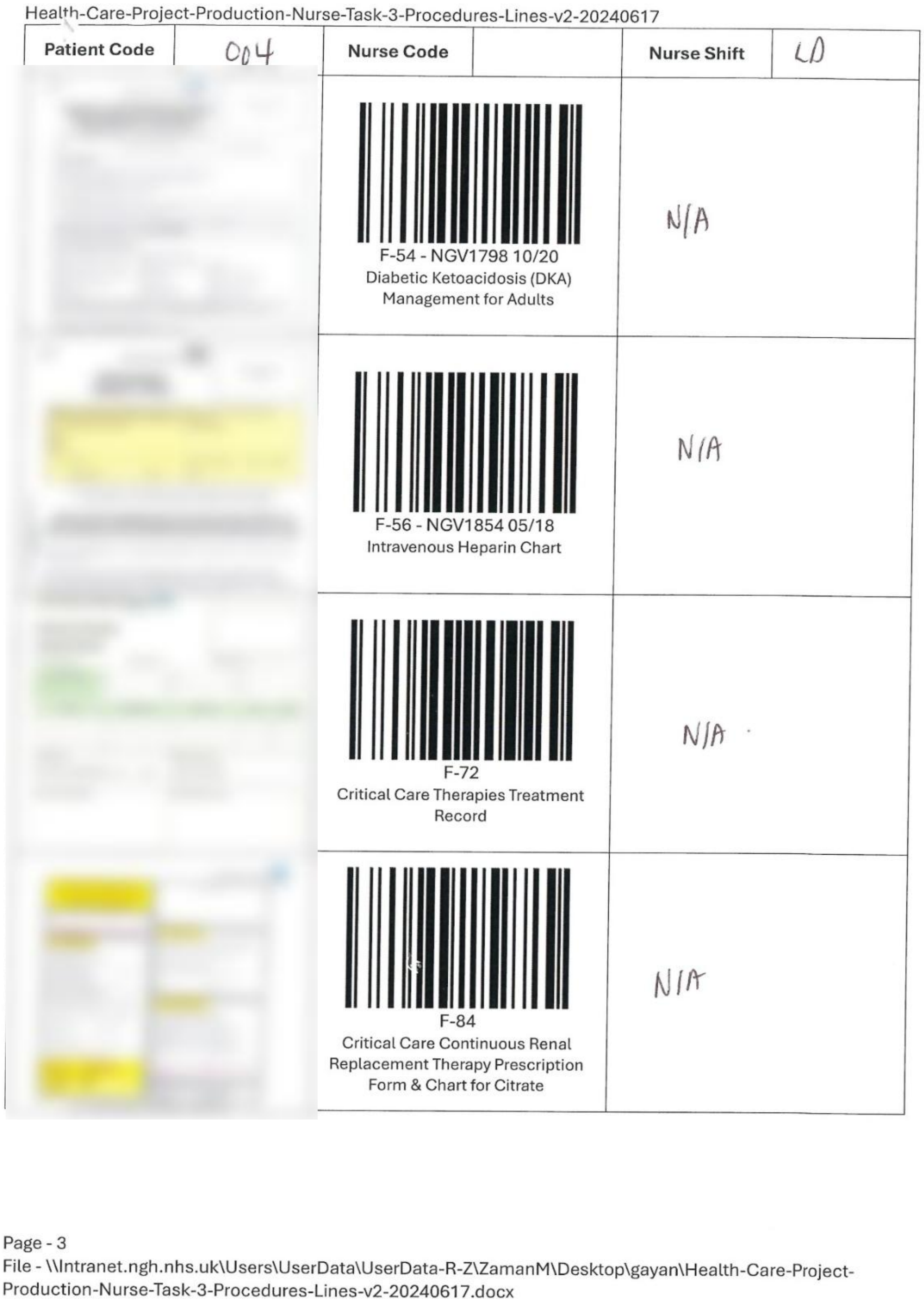

Health-Care-Project-Production-Nurse-Task-3-Procedures-Lines-v2-20240617

Patient Code	004	Nurse Code		Nurse Shift	LD
		F-54 - NGV1798 10/20<br>Diabetic Ketoacidosis (DKA) Management for Adults		N/A	
		F-56 - NGV1854 05/18<br>Intravenous Heparin Chart		N/A	
		F-72<br>Critical Care Therapies Treatment Record		N/A	
		F-84<br>Critical Care Continuous Renal Replacement Therapy Prescription Form & Chart for Citrate		N/A	

Page - 3

File - \\Intranet.ngh.nhs.uk\Users\UserData\UserData-R-Z\ZamanM\Desktop\gayan\Health-Care-Project-Production-Nurse-Task-3-Procedures-Lines-v2-20240617.docx

Supplementary Figure 25: Result page – 25 for procedure-lines discrete event.

No procedure-lines discrete events were recorded using offline method by nursing staff. The demographic data was excluded in during the data collection.

The thumbnails of observation forms used in this figure were blurred for privacy reasons.

Supplementary Figure 26: Result Page 26

Health-Care-Project-Production-Nurse-Task-3-Procedures-Lines-v2-20240617

Patient Code		Nurse Code		Nurse Shift	
		F-97<br>MRI Patient Screening Questionnaire and Consent Form		N/A	

Page - 4
File - \\Intranet.ngh.nhs.uk\Users\UserData\UserData-R-Z\ZamanM\Desktop\gayan\Health-Care-Project-Production-Nurse-Task-3-Procedures-Lines-v2-20240617.docx

Supplementary Figure 26: Result page – 26 for procedure-lines discrete event.

No procedure-lines discrete event was recorded using offline method by nursing staff. The demographic data was excluded in during the data collection.

The thumbnails of observation forms used in this figure were blurred for privacy reasons.

Supplementary Figure 27: Result Page 27

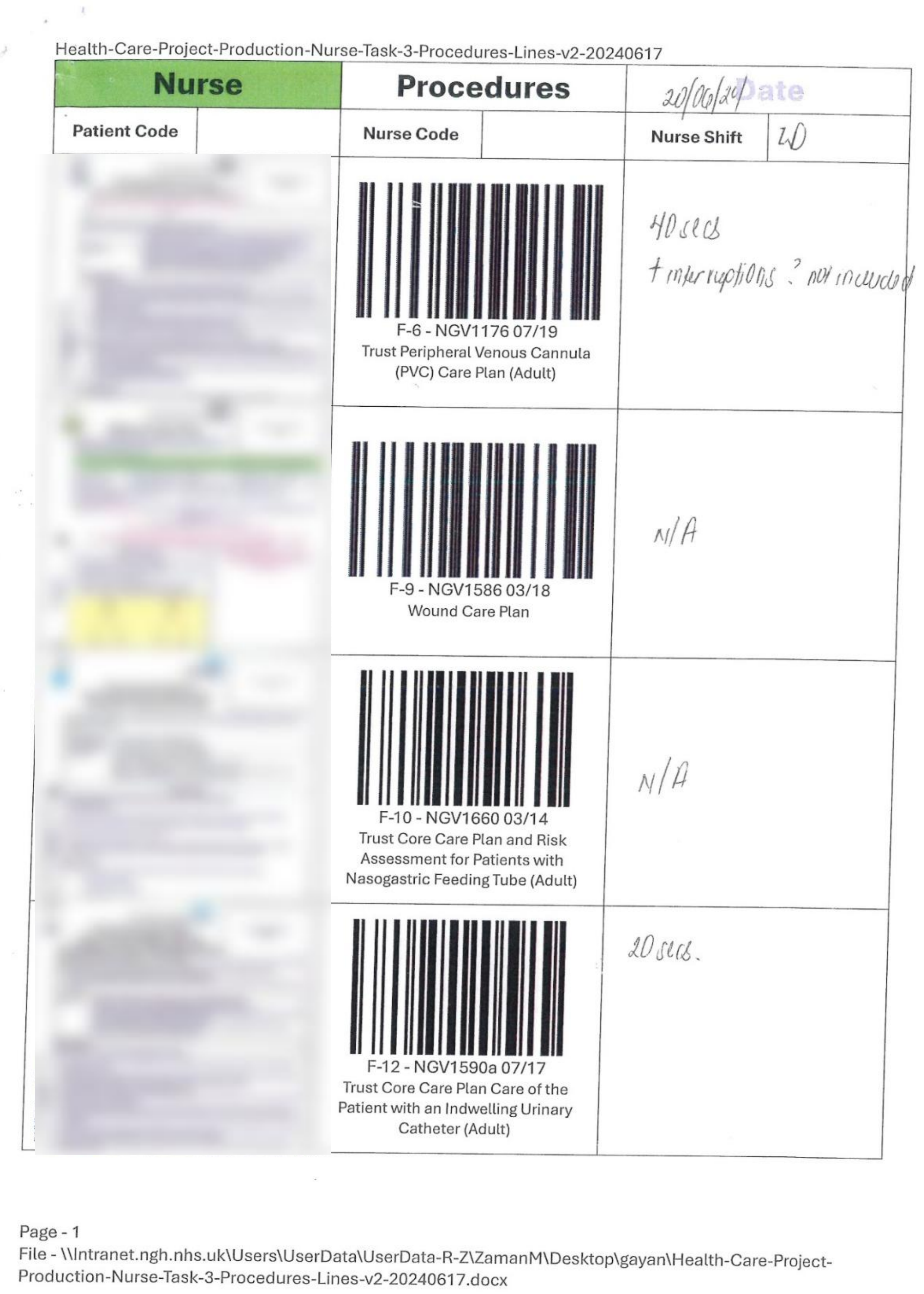

Health-Care-Project-Production-Nurse-Task-3-Procedures-Lines-v2-20240617

Nurse		Procedures		20/06/24 Date	
Patient Code		Nurse Code		Nurse Shift	LD
		F-6 - NGV1176 07/19<br>Trust Peripheral Venous Cannula (PVC) Care Plan (Adult)		40 secs<br>+ interruptions ? not included	
		F-9 - NGV1586 03/18<br>Wound Care Plan		N/A	
		F-10 - NGV1660 03/14<br>Trust Core Care Plan and Risk Assessment for Patients with Nasogastric Feeding Tube (Adult)		N/A	
		F-12 - NGV1590a 07/17<br>Trust Core Care Plan Care of the Patient with an Indwelling Urinary Catheter (Adult)		20 secs.	

Page - 1
File - \\Intranet.ngh.nhs.uk\Users\UserData\UserData-R-Z\ZamanM\Desktop\gayan\Health-Care-Project-Production-Nurse-Task-3-Procedures-Lines-v2-20240617.docx

Supplementary Figure 27: Result page – 27 for procedure-lines discrete event.

These procedure-lines discrete events were recorded using offline method by nursing staff. The demographic data was excluded in during the data collection.

The thumbnails of observation forms used in this figure were blurred for privacy reasons.

Supplementary Figure 28: Result Page 28

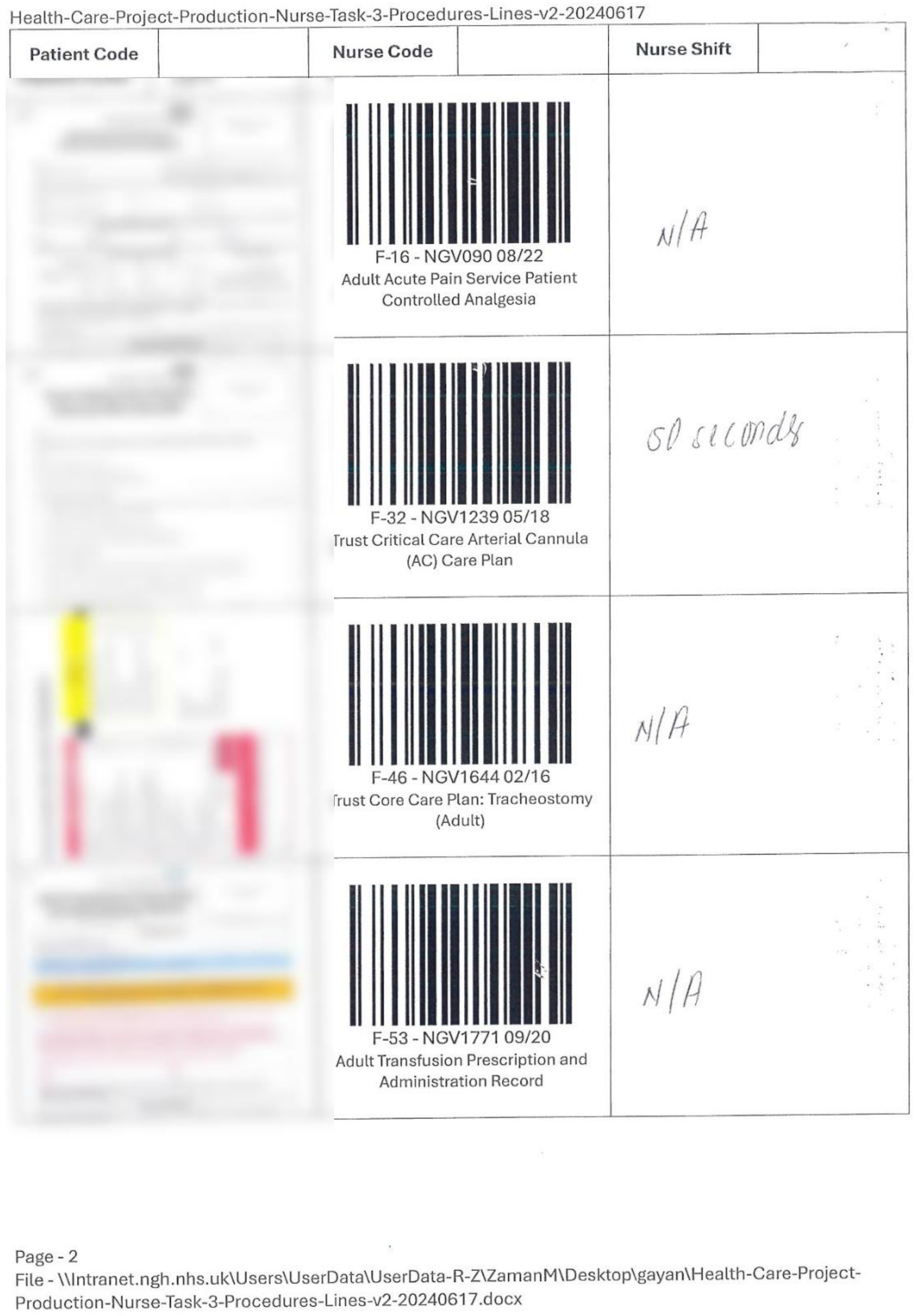

Health-Care-Project-Production-Nurse-Task-3-Procedures-Lines-v2-20240617

Patient Code		Nurse Code		Nurse Shift	
		F-16 - NGV090 08/22<br>Adult Acute Pain Service Patient Controlled Analgesia		N/A	
		F-32 - NGV1239 05/18<br>Trust Critical Care Arterial Cannula (AC) Care Plan		50 seconds	
		F-46 - NGV1644 02/16<br>Trust Core Care Plan: Tracheostomy (Adult)		N/A	
		F-53 - NGV1771 09/20<br>Adult Transfusion Prescription and Administration Record		N/A	

Page - 2
File - \\Intranet.ngh.nhs.uk\Users\UserData\UserData-R-Z\ZamanM\Desktop\gayan\Health-Care-Project-Production-Nurse-Task-3-Procedures-Lines-v2-20240617.docx

Supplementary Figure 28: Result page – 28 for procedure-lines discrete event.

This procedure-lines discrete event was recorded using offline method by nursing staff. The demographic data was excluded in during the data collection.

The thumbnails of observation forms used in this figure were blurred for privacy reasons.

Supplementary Figure 29: Result Page 29

Health-Care-Project-Production-Nurse-Task-3-Procedures-Lines-v2-20240617

Patient Code		Nurse Code		Nurse Shift	
[redacted]		F-54 - NGV1798 10/20<br>Diabetic Ketoacidosis (DKA) Management for Adults		N/A	
[redacted]		F-56 - NGV1854 05/18<br>Intravenous Heparin Chart		N/A	
[redacted]		F-72<br>Critical Care Therapies Treatment Record		N/A	
[redacted]		F-84<br>Critical Care Continuous Renal Replacement Therapy Prescription Form & Chart for Citrate		N/A	

Page - 3
File - \\Intranet.ngh.nhs.uk\Users\UserData\UserData-R-Z\ZamanM\Desktop\gayan\Health-Care-Project-Production-Nurse-Task-3-Procedures-Lines-v2-20240617.docx

Supplementary Figure 29: Result page – 29 for procedure-lines discrete event.

No procedure-lines discrete events were recorded using offline method by nursing staff. The demographic data was excluded in during the data collection.

Supplementary Figure 30: Result Page 30

Health-Care-Project-Production-Nurse-Task-3-Procedures-Lines-v2-20240617

Patient Code		Nurse Code		Nurse Shift	
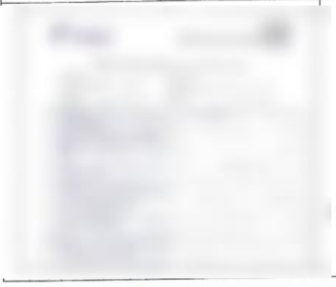		F-97<br>MRI Patient Screening Questionnaire and Consent Form			

Page - 4
File - \\Intranet.ngh.nhs.uk\Users\UserData\UserData-R-Z\ZamanM\Desktop\gayan\Health-Care-Project-Production-Nurse-Task-3-Procedures-Lines-v2-20240617.docx

Supplementary Figure 30: Result page – 30 for procedure-lines discrete event.

No procedure-lines discrete event was recorded using offline method by nursing staff. The demographic data was excluded in during the data collection.

The thumbnails of observation forms used in this figure were blurred for privacy reasons.

Supplementary Figure 31: Result Page 31

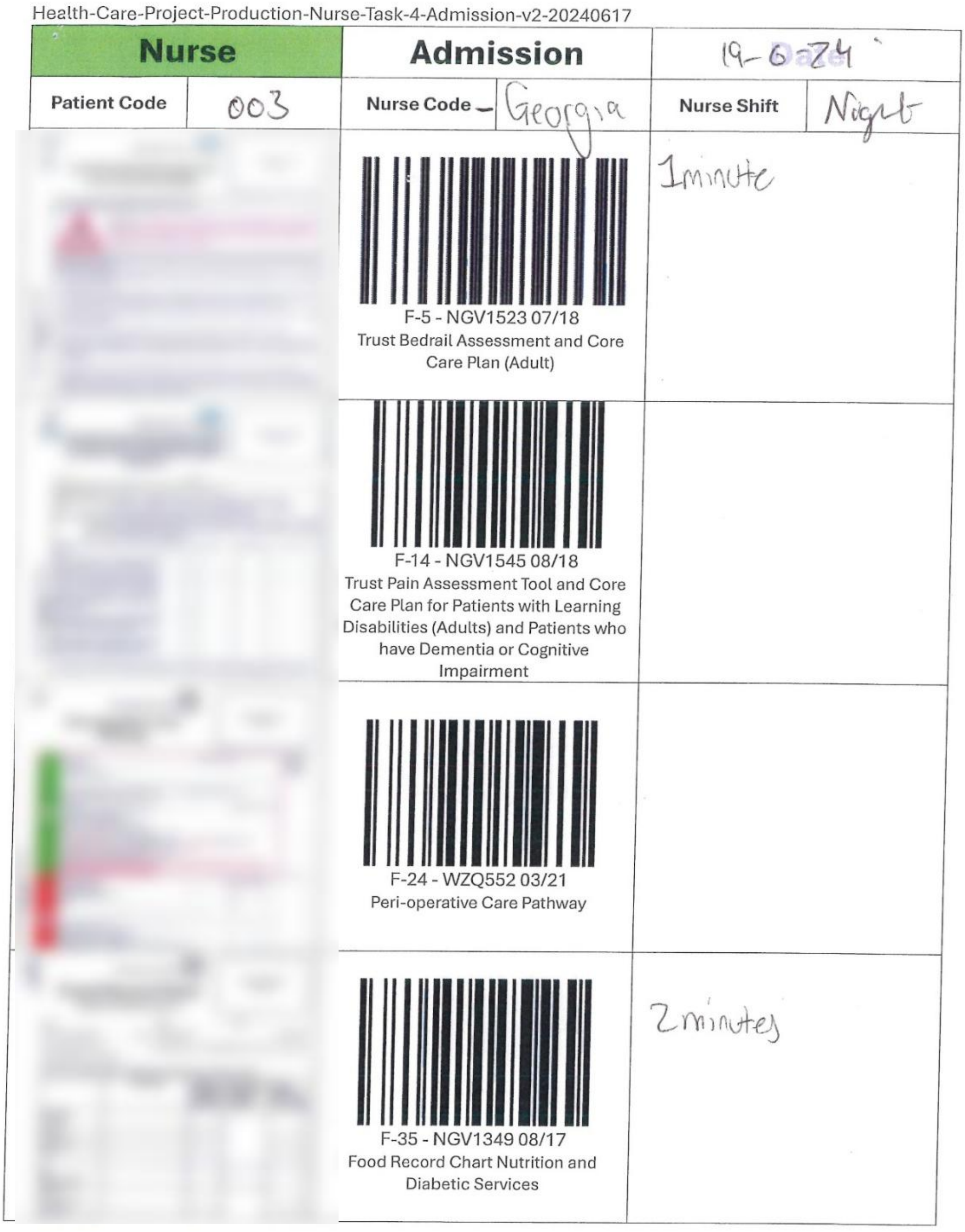

Health-Care-Project-Production-Nurse-Task-4-Admission-v2-20240617

Nurse		Admission		19-6-24	
Patient Code	003	Nurse Code –	Georgia	Nurse Shift	Night
		F-5 - NGV1523 07/18<br>Trust Bedrail Assessment and Core Care Plan (Adult)		1minute	
		F-14 - NGV1545 08/18<br>Trust Pain Assessment Tool and Core Care Plan for Patients with Learning Disabilities (Adults) and Patients who have Dementia or Cognitive Impairment			
		F-24 - WZQ552 03/21<br>Peri-operative Care Pathway			
		F-35 - NGV1349 08/17<br>Food Record Chart Nutrition and Diabetic Services		2minutes	

Page - 1
File - https://d.docs.live.net/f312ce15ac7eb348/Desktop/Healthcare-Project/Production/Health-Care-Project-Production-Nurse-Task-4-Admission-v2-20240617.docx

Supplementary Figure 31: Result page – 31 for admission discrete event.

These admission discrete events were recorded using offline method by nursing staff. The demographic data was excluded in during the data collection.

The thumbnails of observation forms used in this figure were blurred for privacy reasons.

Supplementary Figure 32: Result Page 32

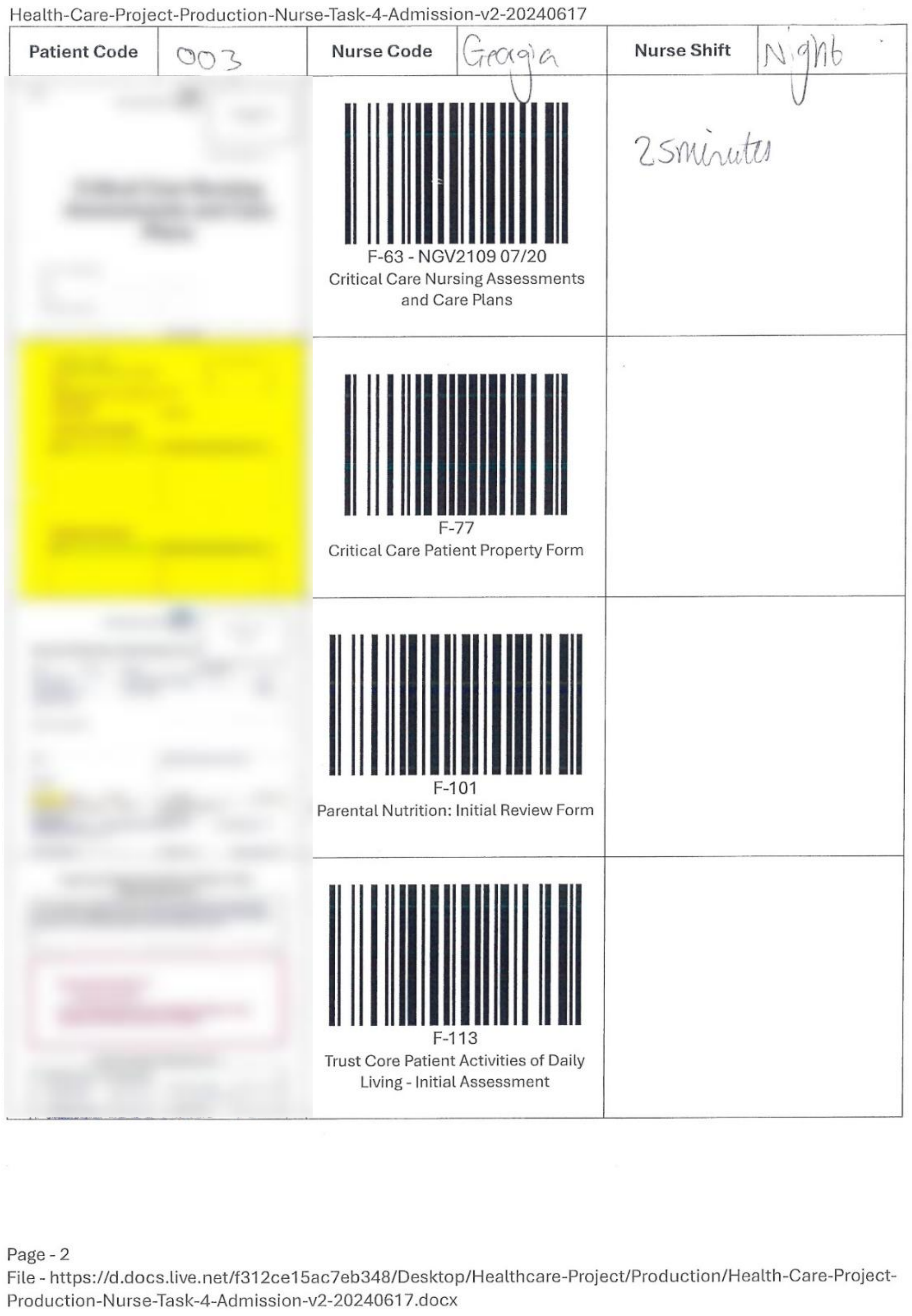

Health-Care-Project-Production-Nurse-Task-4-Admission-v2-20240617

Patient Code	003	Nurse Code	Georgia	Nurse Shift	Night
		F-63 - NGV2109 07/20 Critical Care Nursing Assessments and Care Plans		25minutes	
		F-77 Critical Care Patient Property Form			
		F-101 Parental Nutrition: Initial Review Form			
		F-113 Trust Core Patient Activities of Daily Living - Initial Assessment			

Page - 2
File - https://d.docs.live.net/f312ce15ac7eb348/Desktop/Healthcare-Project/Production/Health-Care-Project-Production-Nurse-Task-4-Admission-v2-20240617.docx

Supplementary Figure 32: Result page – 32 for admission discrete event.

This admission discrete event was recorded using offline method by nursing staff. The demographic data was excluded in during the data collection.

The thumbnails of observation forms used in this figure were blurred for privacy reasons.

Supplementary Figure 33: Result Page 33

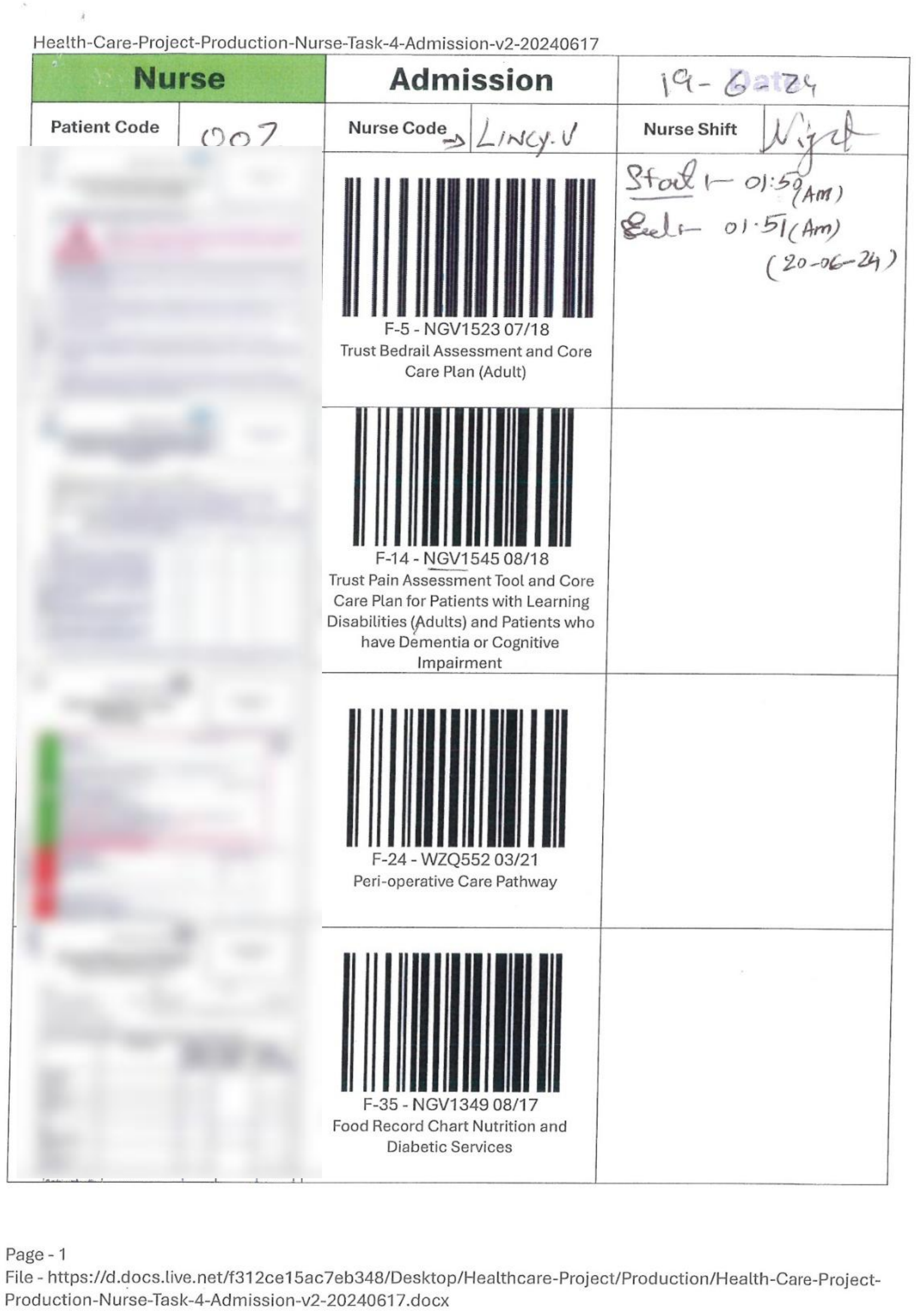

Health-Care-Project-Production-Nurse-Task-4-Admission-v2-20240617

Nurse		Admission		19- [illegible]
Patient Code	007	Nurse Code →	LINCY.V	Nurse Shift	Night
		F-5 - NGV1523 07/18 Trust Bedrail Assessment and Core Care Plan (Adult)		Start – 01:59 (Am) End – 01:51 (Am) (20-06-24)	
		F-14 - NGV1545 08/18 Trust Pain Assessment Tool and Core Care Plan for Patients with Learning Disabilities (Adults) and Patients who have Dementia or Cognitive Impairment			
		F-24 - WZQ552 03/21 Peri-operative Care Pathway			
		F-35 - NGV1349 08/17 Food Record Chart Nutrition and Diabetic Services			

Page - 1
File - https://d.docs.live.net/f312ce15ac7eb348/Desktop/Healthcare-Project/Production/Health-Care-Project-Production-Nurse-Task-4-Admission-v2-20240617.docx

Supplementary Figure 33: Result page – 33 for admission discrete event.

These admission discrete events were recorded using offline method by nursing staff. The demographic data was excluded in during the data collection.

The thumbnails of observation forms used in this figure were blurred for privacy reasons.

Supplementary Figure 34: Result Page 34

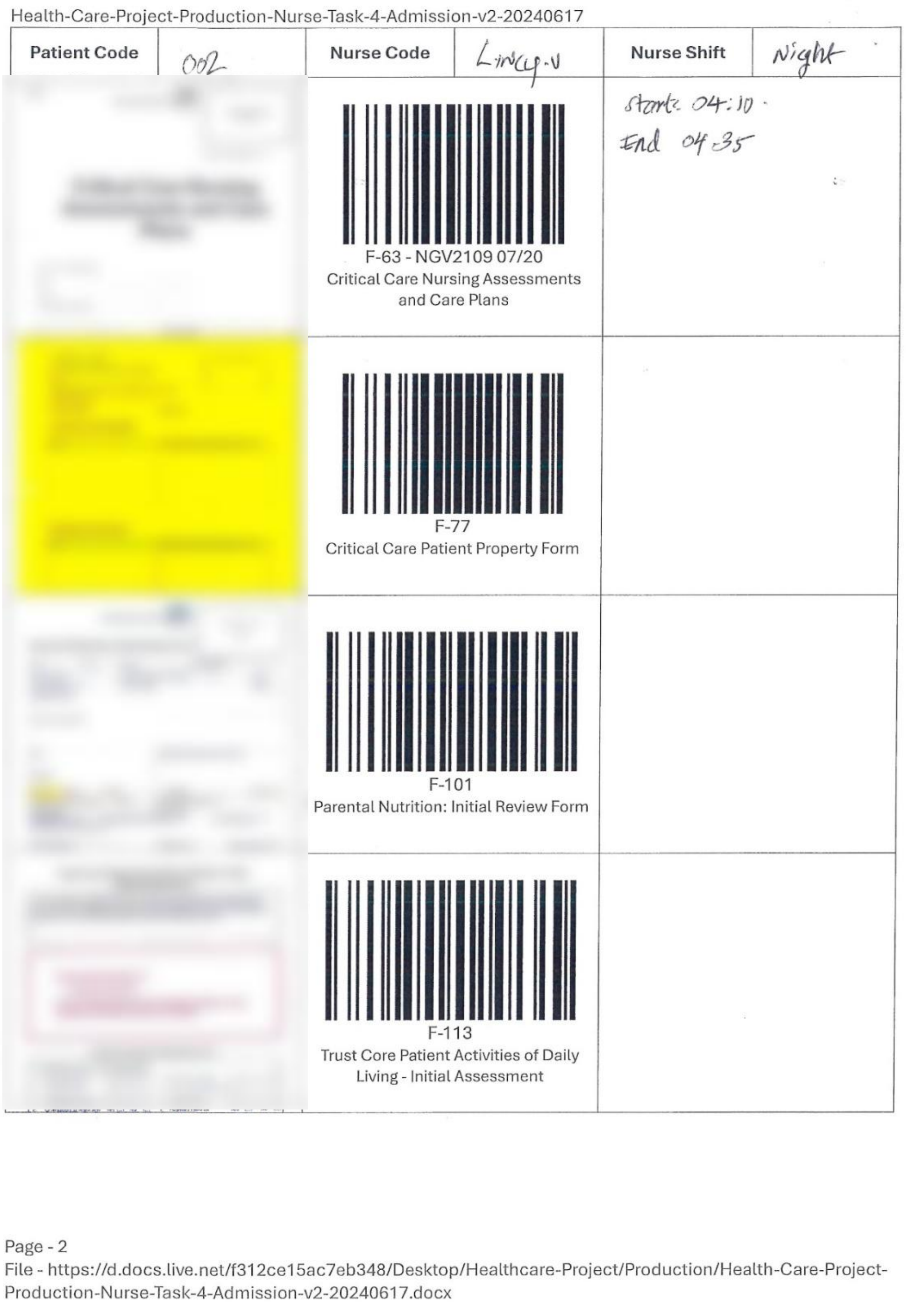

Health-Care-Project-Production-Nurse-Task-4-Admission-v2-20240617

Patient Code	002	Nurse Code	Lincy.V	Nurse Shift	Night
		F-63 - NGV2109 07/20<br>Critical Care Nursing Assessments and Care Plans		Start: 04:10<br>End 04:35	
		F-77<br>Critical Care Patient Property Form			
		F-101<br>Parental Nutrition: Initial Review Form			
		F-113<br>Trust Core Patient Activities of Daily Living - Initial Assessment			

Page - 2
File - https://d.docs.live.net/f312ce15ac7eb348/Desktop/Healthcare-Project/Production/Health-Care-Project-Production-Nurse-Task-4-Admission-v2-20240617.docx

Supplementary Figure 34: Result page – 34 for admission discrete event.

These admission discrete events were recorded using offline method by nursing staff. The demographic data was excluded in during the data collection.

The thumbnails of observation forms used in this figure were blurred for privacy reasons.

Supplementary Figure 35: Result Page 35

Health-Care-Project-Production-Nurse-Task-4-Admission-v2-20240617

Nurse		Admission		19-6-24.	
Patient Code	001	Nurse Code → Kate [illegible]		Nurse Shift	Night.
		F-5 - NGV1523 07/18<br>Trust Bedrail Assessment and Core Care Plan (Adult)		0200 – 0201	
		F-14 - NGV1545 08/18<br>Trust Pain Assessment Tool and Core Care Plan for Patients with Learning Disabilities (Adults) and Patients who have Dementia or Cognitive Impairment		—	
		F-24 - WZQ552 03/21<br>Peri-operative Care Pathway		—	
		F-35 - NGV1349 08/17<br>Food Record Chart Nutrition and Diabetic Services		—	

Page - 1
File - https://d.docs.live.net/f312ce15ac7eb348/Desktop/Healthcare-Project/Production/Health-Care-Project-Production-Nurse-Task-4-Admission-v2-20240617.docx

Supplementary Figure 35: Result page – 35 for admission discrete event.

This admission discrete event was recorded using offline method by nursing staff. The demographic data was excluded in during the data collection.

The thumbnails of observation forms used in this figure were blurred for privacy reasons.

Supplementary Figure 36: Result Page 36

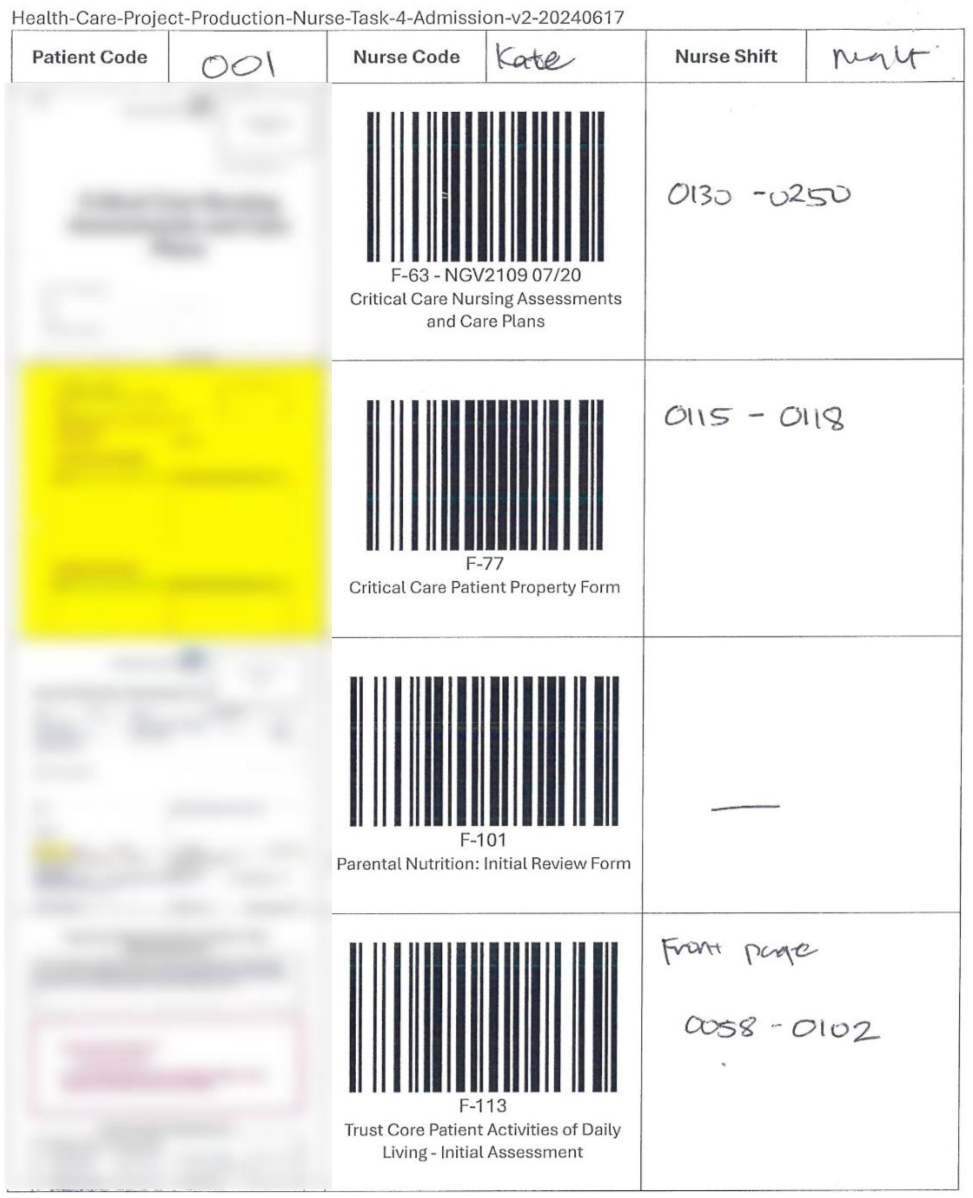

Health-Care-Project-Production-Nurse-Task-4-Admission-v2-20240617

Patient Code	001	Nurse Code	Kate	Nurse Shift	night
		F-63 - NGV2109 07/20<br>Critical Care Nursing Assessments and Care Plans		0130 - 0250	
		F-77<br>Critical Care Patient Property Form		0115 - 0118	
		F-101<br>Parental Nutrition: Initial Review Form		—	
		F-113<br>Trust Core Patient Activities of Daily Living - Initial Assessment		Front page<br>0058 - 0102	

Page - 2
File - https://d.docs.live.net/f312ce15ac7eb348/Desktop/Healthcare-Project/Production/Health-Care-Project-Production-Nurse-Task-4-Admission-v2-20240617.docx

Supplementary Figure 36: Result page – 36 for admission discrete event.

These admission discrete events were recorded using offline method by nursing staff. The demographic data was excluded in during the data collection.

The thumbnails of observation forms used in this figure were blurred for privacy reasons.

Supplementary Figure 37: Result Page 37

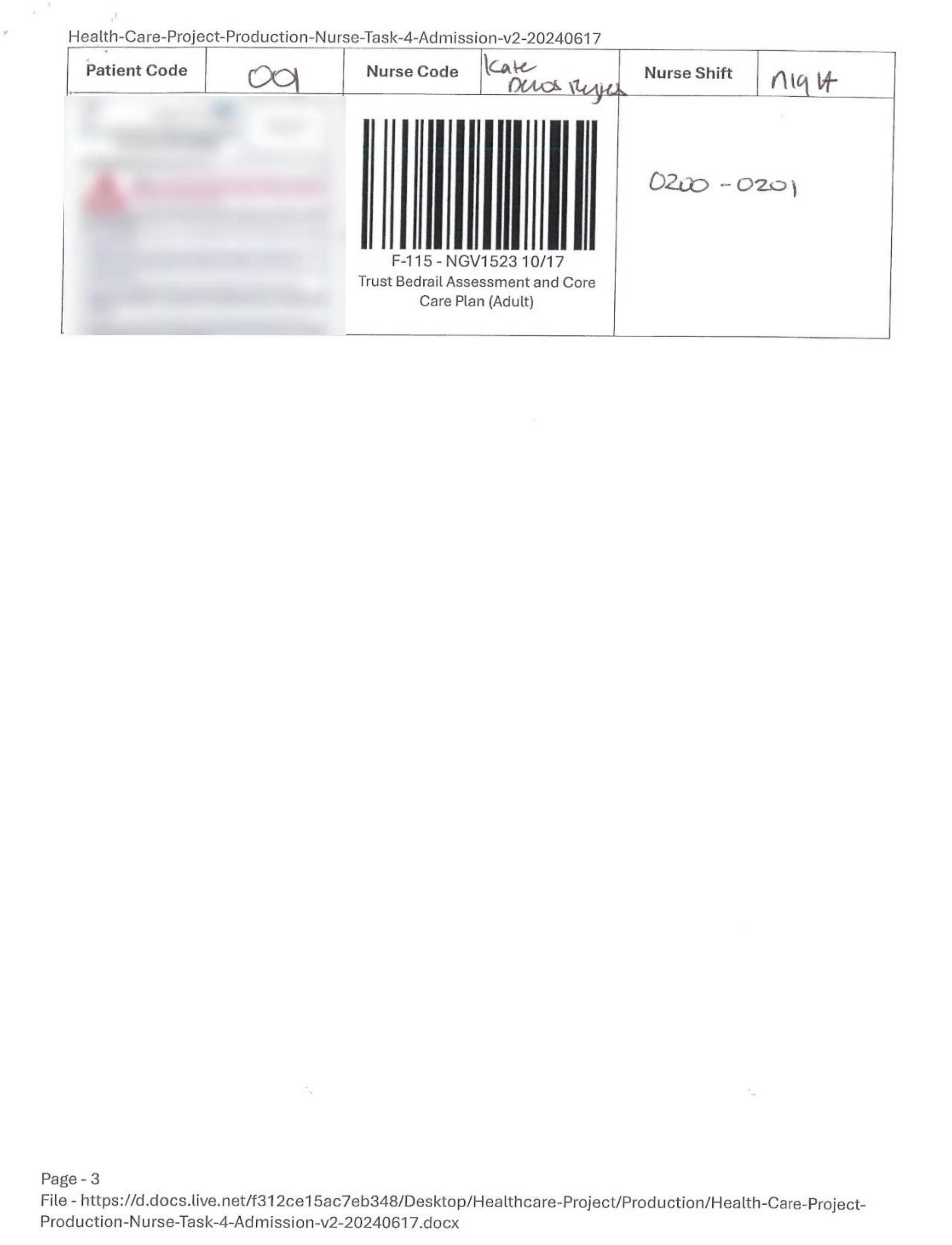

Health-Care-Project-Production-Nurse-Task-4-Admission-v2-20240617

Patient Code	001	Nurse Code	Kate [illegible]	Nurse Shift	night
		F-115 - NGV1523 10/17 Trust Bedrail Assessment and Core Care Plan (Adult)		0200 - 0201	

Page - 3
File - https://d.docs.live.net/f312ce15ac7eb348/Desktop/Healthcare-Project/Production/Health-Care-Project-Production-Nurse-Task-4-Admission-v2-20240617.docx

Supplementary Figure 37: Result page – 37 for admission discrete event.

This admission discrete event was recorded using offline method by nursing staff. The demographic data was excluded in during the data collection.

The thumbnails of observation forms used in this figure were blurred for privacy reasons.

Supplementary Figure 38: Result Page 38

Health-Care-Project-Production-Nurse-Task-4-Admission-v2-20240617

Nurse		Admission		Date 20/06/24	
Patient Code		Nurse Code		Nurse Shift	40
		F-5 - NGV1523 07/18<br>Trust Bedrail Assessment and Core Care Plan (Adult)		30 seconds	
		F-14 - NGV1545 08/18<br>Trust Pain Assessment Tool and Core Care Plan for Patients with Learning Disabilities (Adults) and Patients who have Dementia or Cognitive Impairment		N/A	
		F-24 - WZQ552 03/21<br>Peri-operative Care Pathway		N/A	
		F-35 - NGV1349 08/17<br>Food Record Chart Nutrition and Diabetic Services		N/A	

Page - 1

File - https://d.docs.live.net/f312ce15ac7eb348/Desktop/Healthcare-Project/Production/Health-Care-Project-Production-Nurse-Task-4-Admission-v2-20240617.docx

Supplementary Figure 38: Result page – 38 for admission discrete event.

This admission discrete event was recorded using offline method by nursing staff. The demographic data was excluded in during the data collection.

The thumbnails of observation forms used in this figure were blurred for privacy reasons.

Supplementary Figure 39: Result Page 39

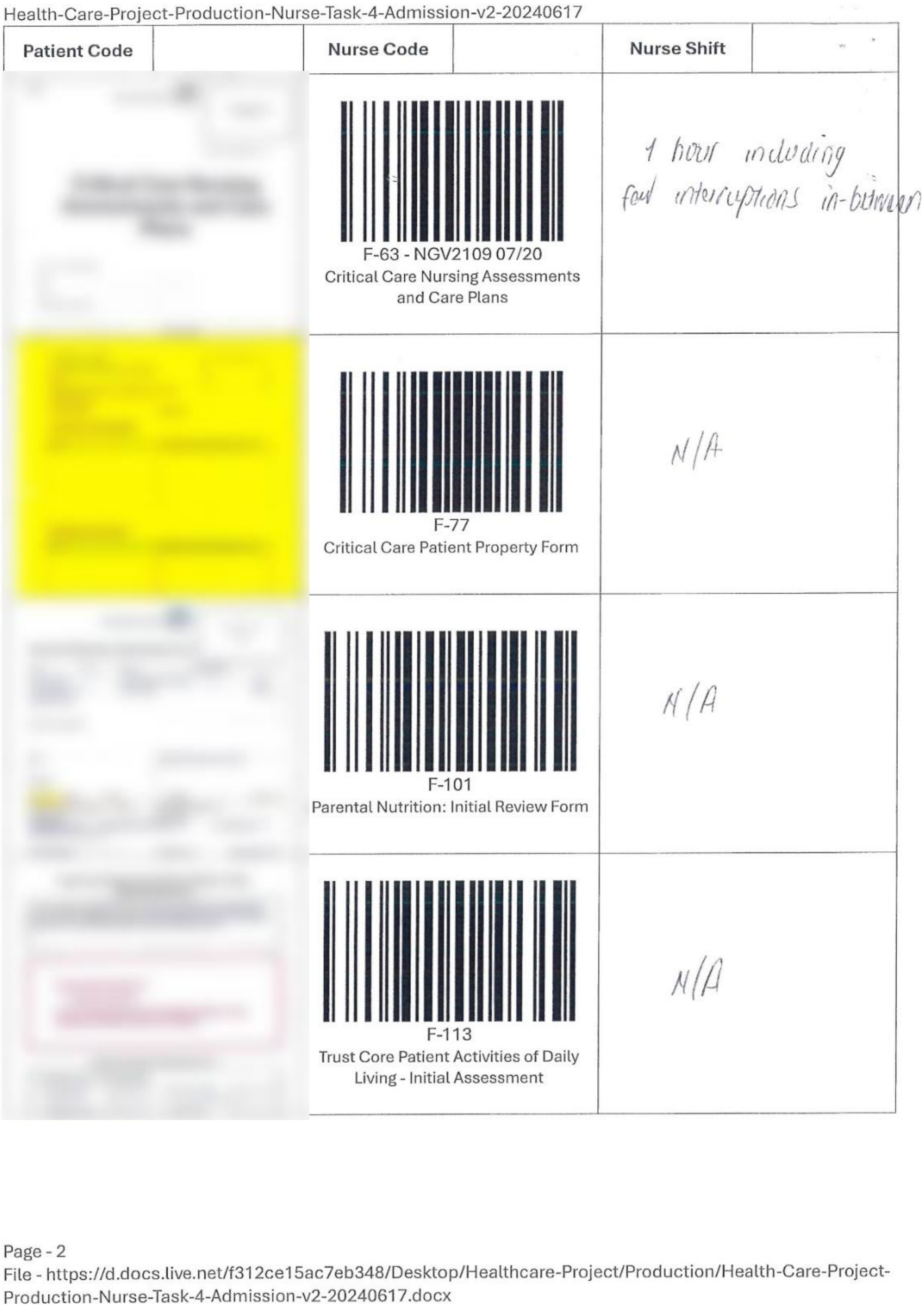

Health-Care-Project-Production-Nurse-Task-4-Admission-v2-20240617

Patient Code		Nurse Code		Nurse Shift	
		F-63 - NGV2109 07/20<br>Critical Care Nursing Assessments and Care Plans		1 hour including four interruptions in-between	
		F-77<br>Critical Care Patient Property Form		N/A	
		F-101<br>Parental Nutrition: Initial Review Form		N/A	
		F-113<br>Trust Core Patient Activities of Daily Living - Initial Assessment		N/A	

Page - 2
File - https://d.docs.live.net/f312ce15ac7eb348/Desktop/Healthcare-Project/Production/Health-Care-Project-Production-Nurse-Task-4-Admission-v2-20240617.docx

Supplementary Figure 39: Result page – 39 for admission discrete event.

This admission discrete event was recorded using offline method by nursing staff. The demographic data was excluded in during the data collection.

The thumbnails of observation forms used in this figure were blurred for privacy reasons.

Supplementary Figure 40: Result Page 40

Health-Care-Project-Production-Nurse-Task-4-Admission-v2-20240617

Patient Code		Nurse Code		Nurse Shift	LD
[illegible]		F-115 - NGV1523 10/17<br>Trust Bedrail Assessment and Core Care Plan (Adult)		30 secs	

Page - 3
File - https://d.docs.live.net/f312ce15ac7eb348/Desktop/Healthcare-Project/Production/Health-Care-Project-Production-Nurse-Task-4-Admission-v2-20240617.docx

Supplementary Figure 40: Result page – 40 for admission discrete event.

This admission discrete event was recorded using offline method by nursing staff. The demographic data was excluded in during the data collection.

The thumbnails of observation forms used in this figure were blurred for privacy reasons.

Supplementary Figure 41: Result Page 41

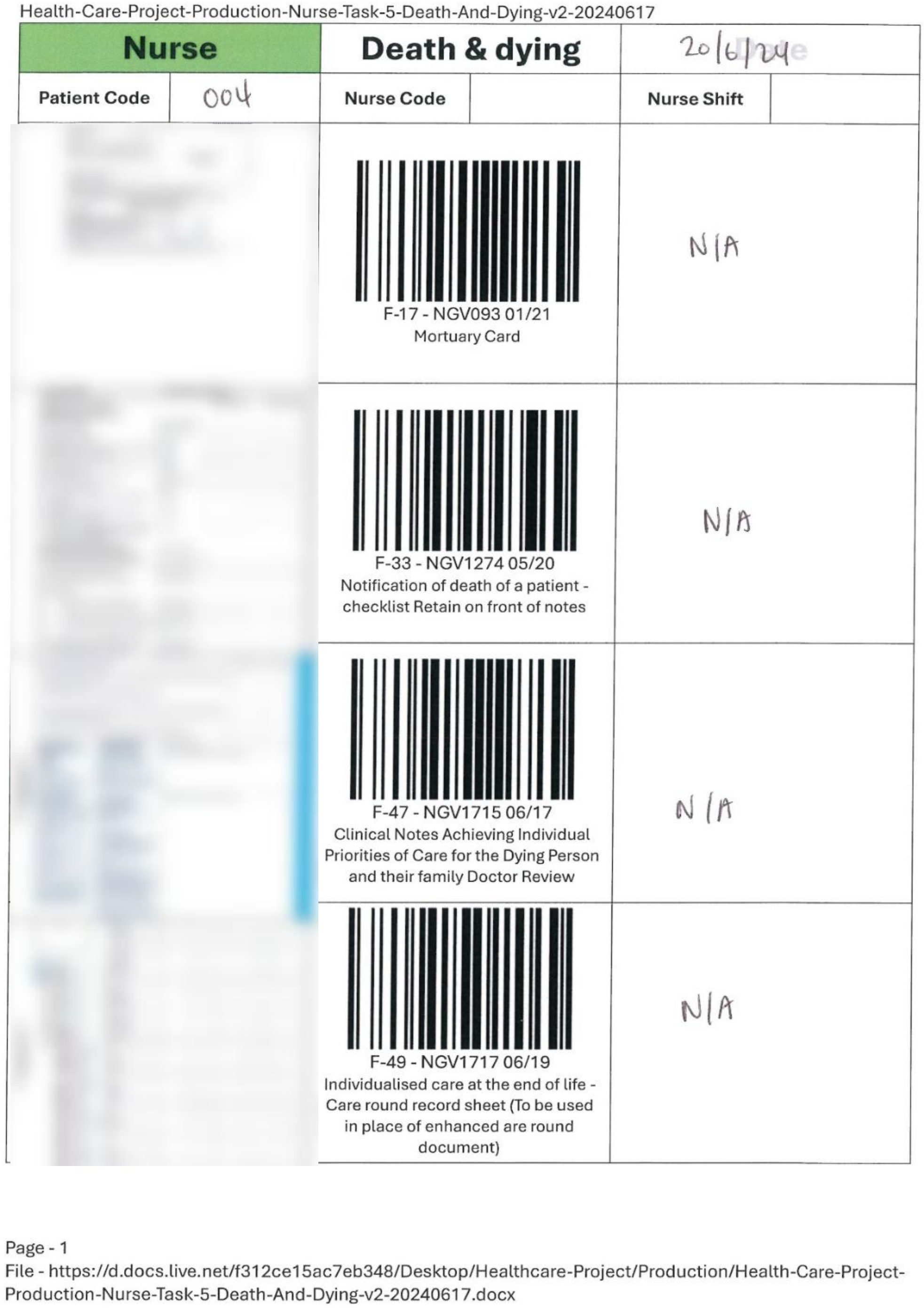

Health-Care-Project-Production-Nurse-Task-5-Death-And-Dying-v2-20240617

Nurse		Death & dying		20/6/24	
Patient Code	004	Nurse Code		Nurse Shift	

	F-17 - NGV093 01/21<br>Mortuary Card	N/A
	F-33 - NGV1274 05/20<br>Notification of death of a patient - checklist Retain on front of notes	N/A
	F-47 - NGV1715 06/17<br>Clinical Notes Achieving Individual Priorities of Care for the Dying Person and their family Doctor Review	N/A
	F-49 - NGV1717 06/19<br>Individualised care at the end of life - Care round record sheet (To be used in place of enhanced are round document)	N/A

Page - 1
File - https://d.docs.live.net/f312ce15ac7eb348/Desktop/Healthcare-Project/Production/Health-Care-Project-Production-Nurse-Task-5-Death-And-Dying-v2-20240617.docx

Supplementary Figure 41: Result page – 41 for death and dying discrete event.

No death and dying discrete events were recorded using offline method by nursing staff. The demographic data was excluded in during the data collection.

The thumbnails of observation forms used in this figure were blurred for privacy reasons.

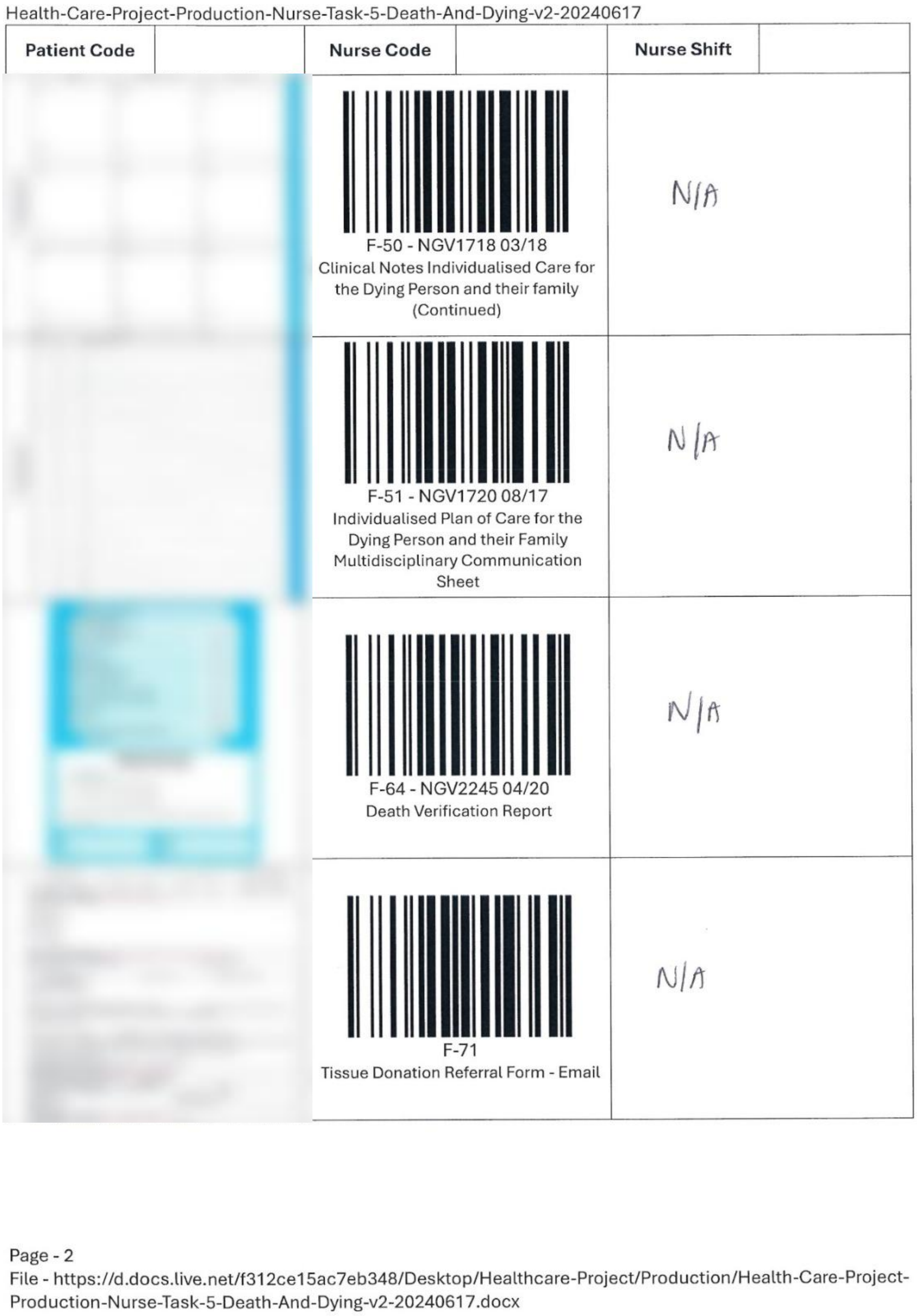

Health-Care-Project-Production-Nurse-Task-5-Death-And-Dying-v2-20240617

Patient Code		Nurse Code		Nurse Shift	
		F-50 - NGV1718 03/18<br>Clinical Notes Individualised Care for the Dying Person and their family (Continued)		N/A	
		F-51 - NGV1720 08/17<br>Individualised Plan of Care for the Dying Person and their Family Multidisciplinary Communication Sheet		N/A	
		F-64 - NGV2245 04/20<br>Death Verification Report		N/A	
		F-71<br>Tissue Donation Referral Form - Email		N/A	

Page - 2
File - https://d.docs.live.net/f312ce15ac7eb348/Desktop/Healthcare-Project/Production/Health-Care-Project-Production-Nurse-Task-5-Death-And-Dying-v2-20240617.docx

Supplementary Figure 42: Result page – 42 for death and dying discrete event.

No death and dying discrete events were recorded using offline method by nursing staff. The demographic data was excluded in during the data collection.

The thumbnails of observation forms used in this figure were blurred for privacy reasons.

Supplementary Figure 43: Result Page 43

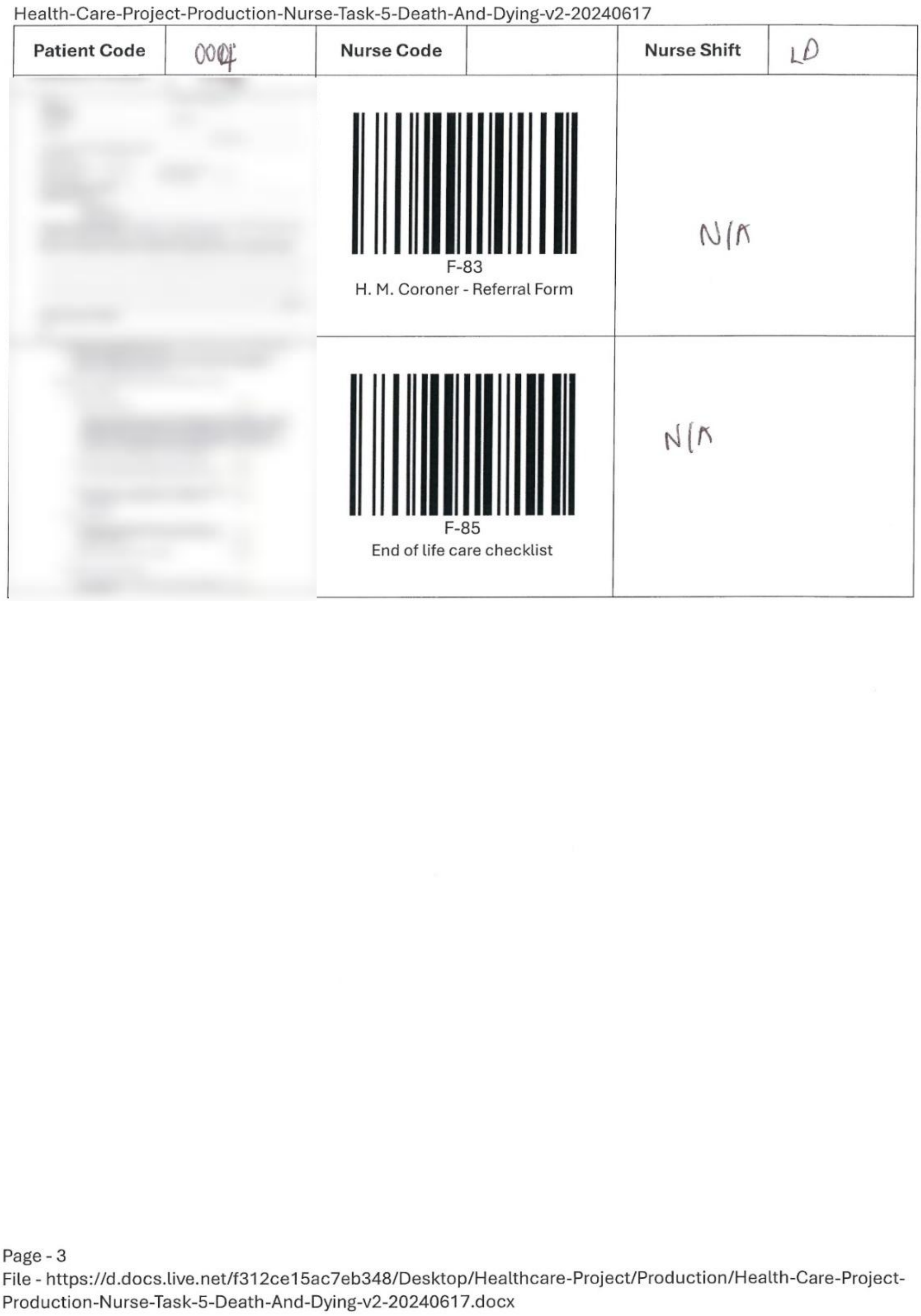

Health-Care-Project-Production-Nurse-Task-5-Death-And-Dying-v2-20240617

Patient Code	0004	Nurse Code		Nurse Shift	LD
		F-83<br>H. M. Coroner - Referral Form		N/A	
		F-85<br>End of life care checklist		N/A	

Page - 3
File - https://d.docs.live.net/f312ce15ac7eb348/Desktop/Healthcare-Project/Production/Health-Care-Project-Production-Nurse-Task-5-Death-And-Dying-v2-20240617.docx

Supplementary Figure 43: Result page – 43 for death and dying discrete event.

No death and dying discrete events were recorded using offline method by nursing staff. The demographic data was excluded in during the data collection.

The thumbnails of observation forms used in this figure were blurred for privacy reasons.

Supplementary Figure 44: Result Page 44

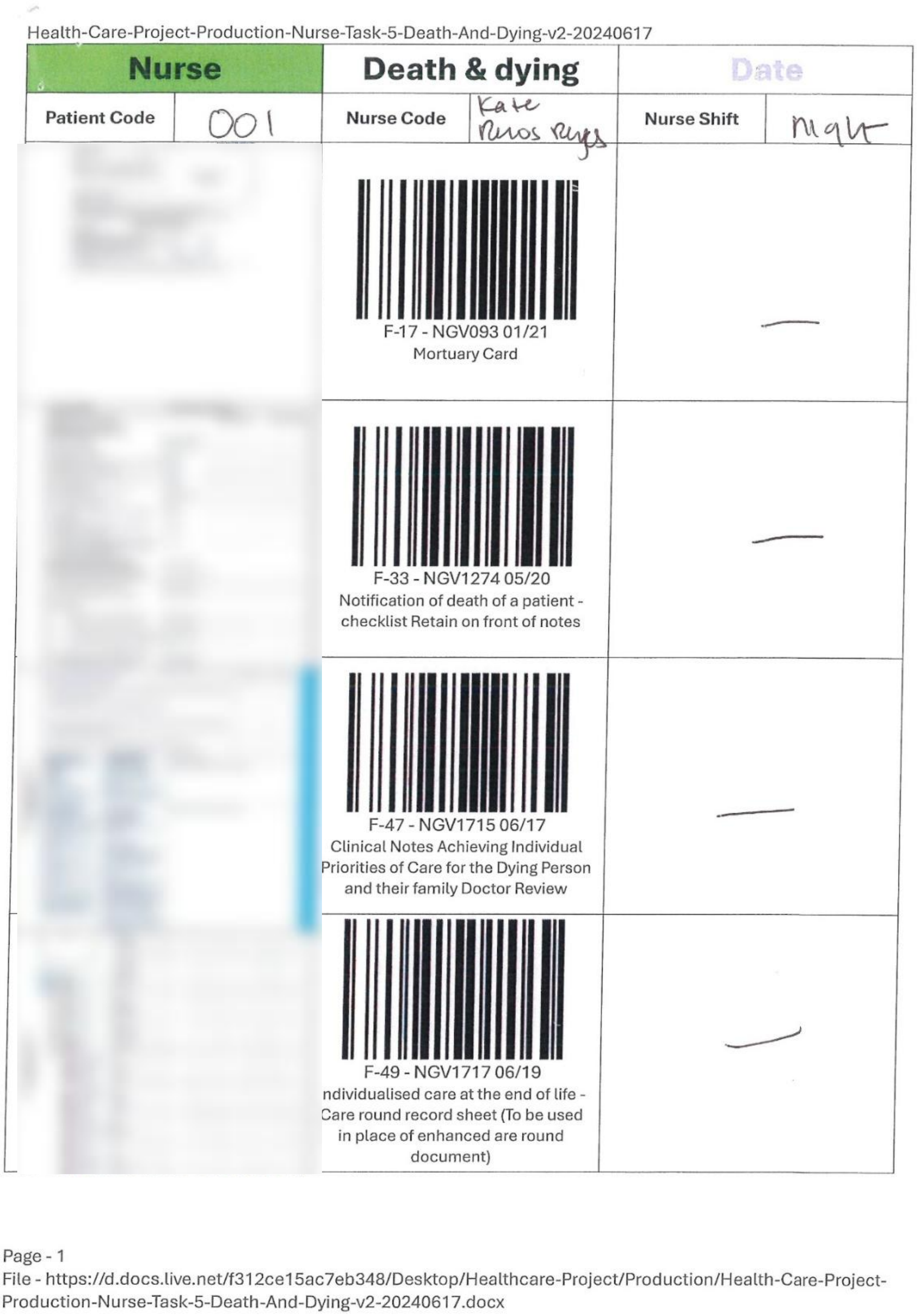

Health-Care-Project-Production-Nurse-Task-5-Death-And-Dying-v2-20240617

Nurse		Death & dying		Date	
Patient Code	001	Nurse Code	Kate Rios Reyes	Nurse Shift	night
		F-17 - NGV093 01/21<br>Mortuary Card		—	
		F-33 - NGV1274 05/20<br>Notification of death of a patient - checklist Retain on front of notes		—	
		F-47 - NGV1715 06/17<br>Clinical Notes Achieving Individual Priorities of Care for the Dying Person and their family Doctor Review		—	
		F-49 - NGV1717 06/19<br>ndividualised care at the end of life - Care round record sheet (To be used in place of enhanced are round document)		—	

Page - 1
File - https://d.docs.live.net/f312ce15ac7eb348/Desktop/Healthcare-Project/Production/Health-Care-Project-Production-Nurse-Task-5-Death-And-Dying-v2-20240617.docx

Supplementary Figure 44: Result page – 44 for death and dying discrete event.

No death and dying discrete events were recorded using offline method by nursing staff. The demographic data was excluded in during the data collection.

The thumbnails of observation forms used in this figure were blurred for privacy reasons.

Supplementary Figure 45: Result Page 45

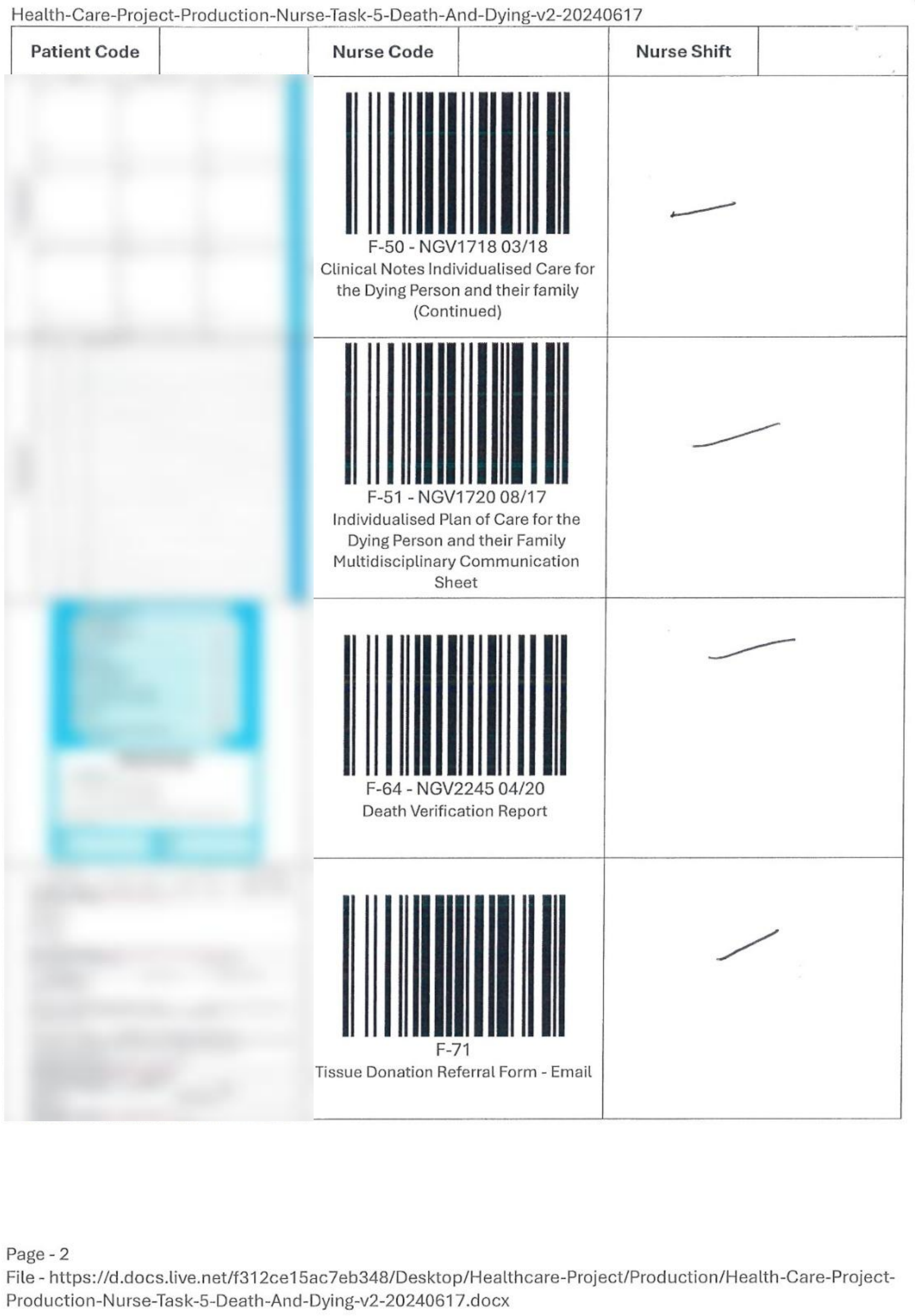

Health-Care-Project-Production-Nurse-Task-5-Death-And-Dying-v2-20240617

Patient Code		Nurse Code		Nurse Shift	
		F-50 - NGV1718 03/18<br>Clinical Notes Individualised Care for the Dying Person and their family (Continued)			
		F-51 - NGV1720 08/17<br>Individualised Plan of Care for the Dying Person and their Family Multidisciplinary Communication Sheet			
		F-64 - NGV2245 04/20<br>Death Verification Report			
		F-71<br>Tissue Donation Referral Form - Email			

Page - 2
File - https://d.docs.live.net/f312ce15ac7eb348/Desktop/Healthcare-Project/Production/Health-Care-Project-Production-Nurse-Task-5-Death-And-Dying-v2-20240617.docx

Supplementary Figure 45: Result page – 45 for death and dying discrete event.

No death and dying discrete events were recorded using offline method by nursing staff. The demographic data was excluded in during the data collection.

The thumbnails of observation forms used in this figure were blurred for privacy reasons.

Supplementary Figure 46: Result Page 46

Health-Care-Project-Production-Nurse-Task-5-Death-And-Dying-v2-20240617

Patient Code		Nurse Code		Nurse Shift	
		F-83<br>H. M. Coroner - Referral Form			
		F-85<br>End of life care checklist			

Page - 3
File - https://d.docs.live.net/f312ce15ac7eb348/Desktop/Healthcare-Project/Production/Health-Care-Project-Production-Nurse-Task-5-Death-And-Dying-v2-20240617.docx

Supplementary Figure 46: Result page – 46 for death and dying discrete event.

No death and dying discrete events were recorded using offline method by nursing staff. The demographic data was excluded in during the data collection.

The thumbnails of observation forms used in this figure were blurred for privacy reasons.

Supplementary Figure 47: Result Page 47

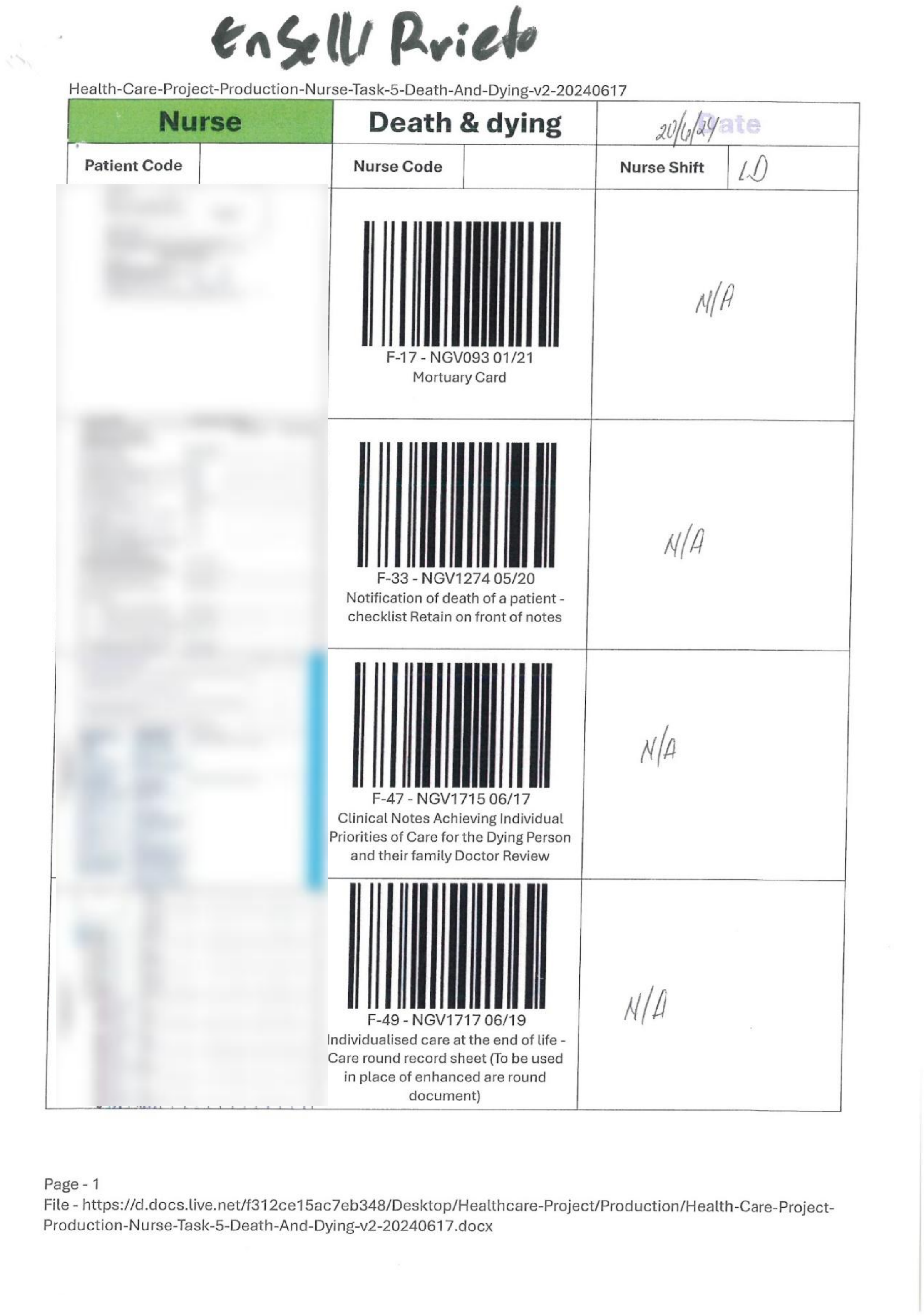

Ensell Prieto

Health-Care-Project-Production-Nurse-Task-5-Death-And-Dying-v2-20240617

Nurse		Death & dying		20/6/24 Date	
Patient Code		Nurse Code		Nurse Shift	LD
		F-17 - NGV093 01/21 Mortuary Card		N/A	
		F-33 - NGV1274 05/20 Notification of death of a patient - checklist Retain on front of notes		N/A	
		F-47 - NGV1715 06/17 Clinical Notes Achieving Individual Priorities of Care for the Dying Person and their family Doctor Review		N/A	
		F-49 - NGV1717 06/19 Individualised care at the end of life - Care round record sheet (To be used in place of enhanced are round document)		N/A	

Page - 1
File - https://d.docs.live.net/f312ce15ac7eb348/Desktop/Healthcare-Project/Production/Health-Care-Project-Production-Nurse-Task-5-Death-And-Dying-v2-20240617.docx

Supplementary Figure 47: Result page – 47 for death and dying discrete event.

No death and dying discrete events were recorded using offline method by nursing staff. The demographic data was excluded in during the data collection.

The thumbnails of observation forms used in this figure were blurred for privacy reasons.

Supplementary Figure 48: Result Page 48

[illegible]

Health-Care-Project-Production-Nurse-Task-5-Death-And-Dying-v2-20240617

Patient Code		Nurse Code		Nurse Shift	
		F-50 - NGV1718 03/18<br>Clinical Notes Individualised Care for the Dying Person and their family (Continued)		N/A	
		F-51 - NGV1720 08/17<br>Individualised Plan of Care for the Dying Person and their Family Multidisciplinary Communication Sheet		N/A	
		F-64 - NGV2245 04/20<br>Death Verification Report		N/A	
		F-71<br>Tissue Donation Referral Form - Email		N/A	

Page - 2
File - https://d.docs.live.net/f312ce15ac7eb348/Desktop/Healthcare-Project/Production/Health-Care-Project-Production-Nurse-Task-5-Death-And-Dying-v2-20240617.docx

Supplementary Figure 48: Result page – 48 for death and dying discrete event.

No death and dying discrete events were recorded using offline method by nursing staff. The demographic data was excluded in during the data collection.

The thumbnails of observation forms used in this figure were blurred for privacy reasons.

Supplementary Figure 49: Result Page 49

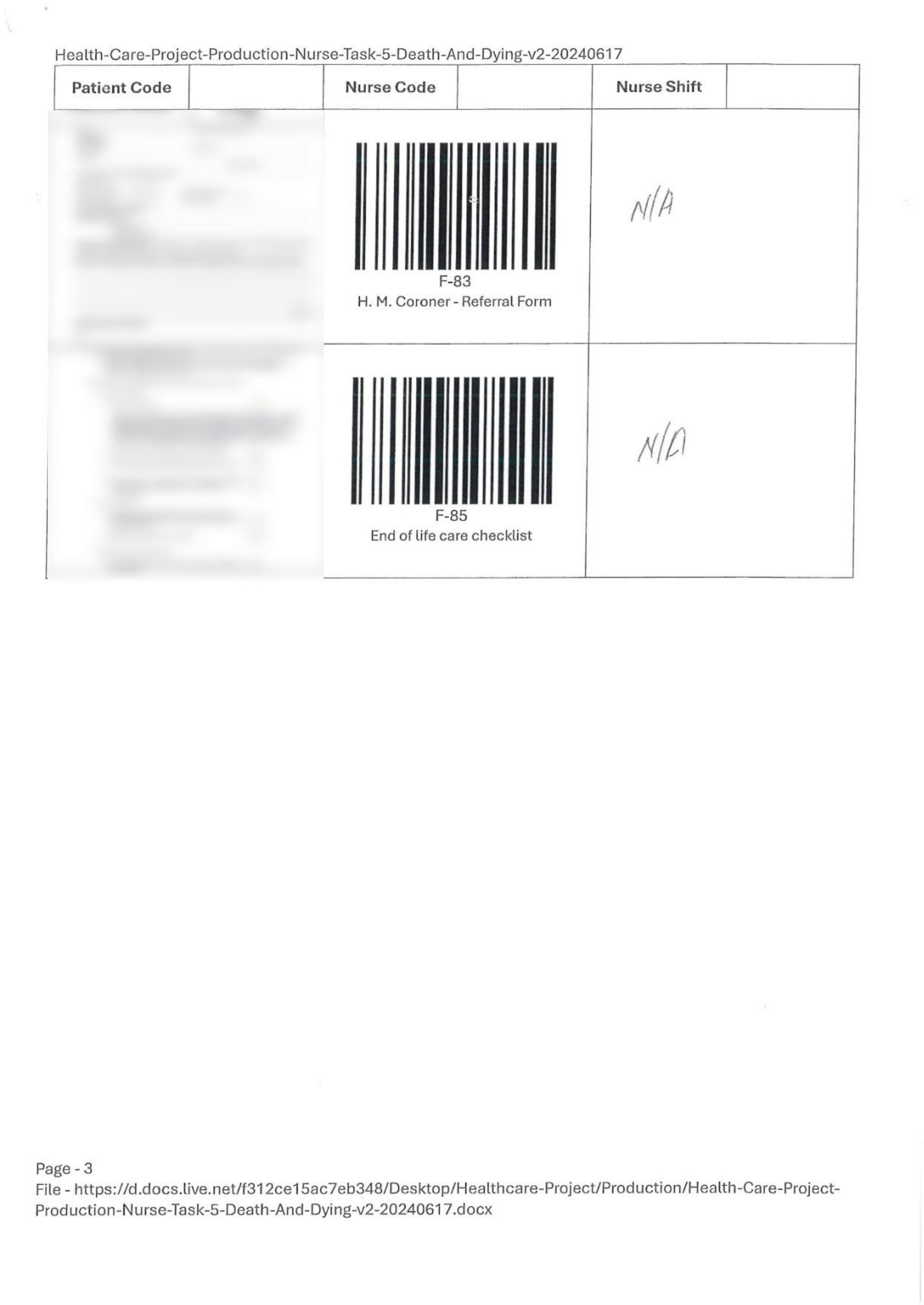

Health-Care-Project-Production-Nurse-Task-5-Death-And-Dying-v2-20240617

Patient Code		Nurse Code		Nurse Shift	
		F-83<br>H. M. Coroner - Referral Form		N/A	
		F-85<br>End of life care checklist		N/A	

Page - 3
File - https://d.docs.live.net/f312ce15ac7eb348/Desktop/Healthcare-Project/Production/Health-Care-Project-Production-Nurse-Task-5-Death-And-Dying-v2-20240617.docx

Supplementary Figure 49: Result page – 49 for death and dying discrete event.

No death and dying discrete events were recorded using offline method by nursing staff. The demographic data was excluded in during the data collection.

The thumbnails of observation forms used in this figure were blurred for privacy reasons.

Supplementary Figure 50: Result Page 50

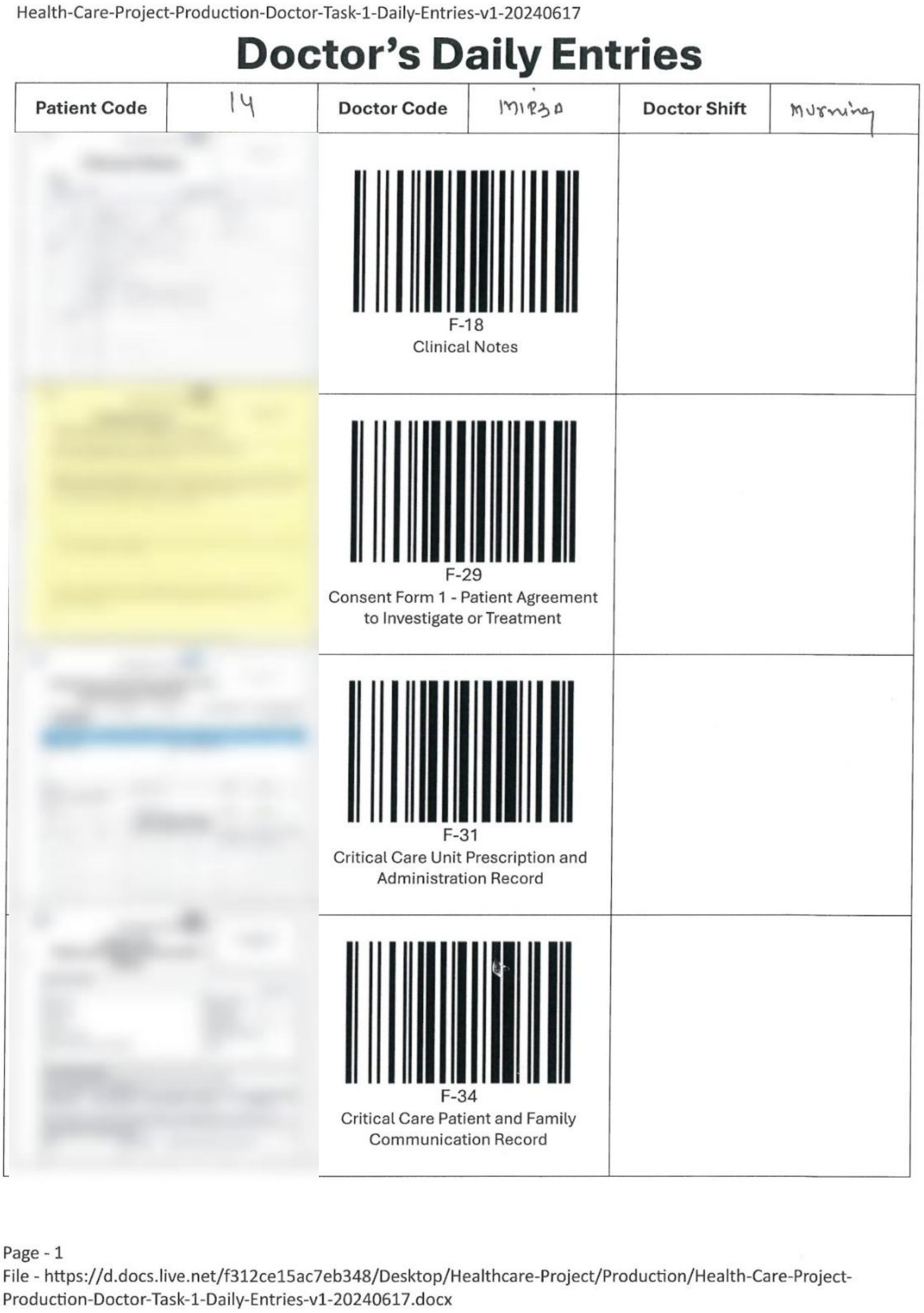

Health-Care-Project-Production-Doctor-Task-1-Daily-Entries-v1-20240617

Doctor's Daily Entries

Patient Code	14	Doctor Code	M1P3D	Doctor Shift	Morning
		F-18 Clinical Notes			
		F-29 Consent Form 1 - Patient Agreement to Investigate or Treatment			
		F-31 Critical Care Unit Prescription and Administration Record			
		F-34 Critical Care Patient and Family Communication Record			

Page - 1
File - https://d.docs.live.net/f312ce15ac7eb348/Desktop/Healthcare-Project/Production/Health-Care-Project-Production-Doctor-Task-1-Daily-Entries-v1-20240617.docx

Supplementary Figure 50: Result page – 50 for daily entries discrete event.

No daily entries discrete events were recorded using offline method by doctors staff. The demographic data was excluded in during the data collection.

The thumbnails of observation forms used in this figure were blurred for privacy reasons.

Supplementary Figure 51: Result Page 51

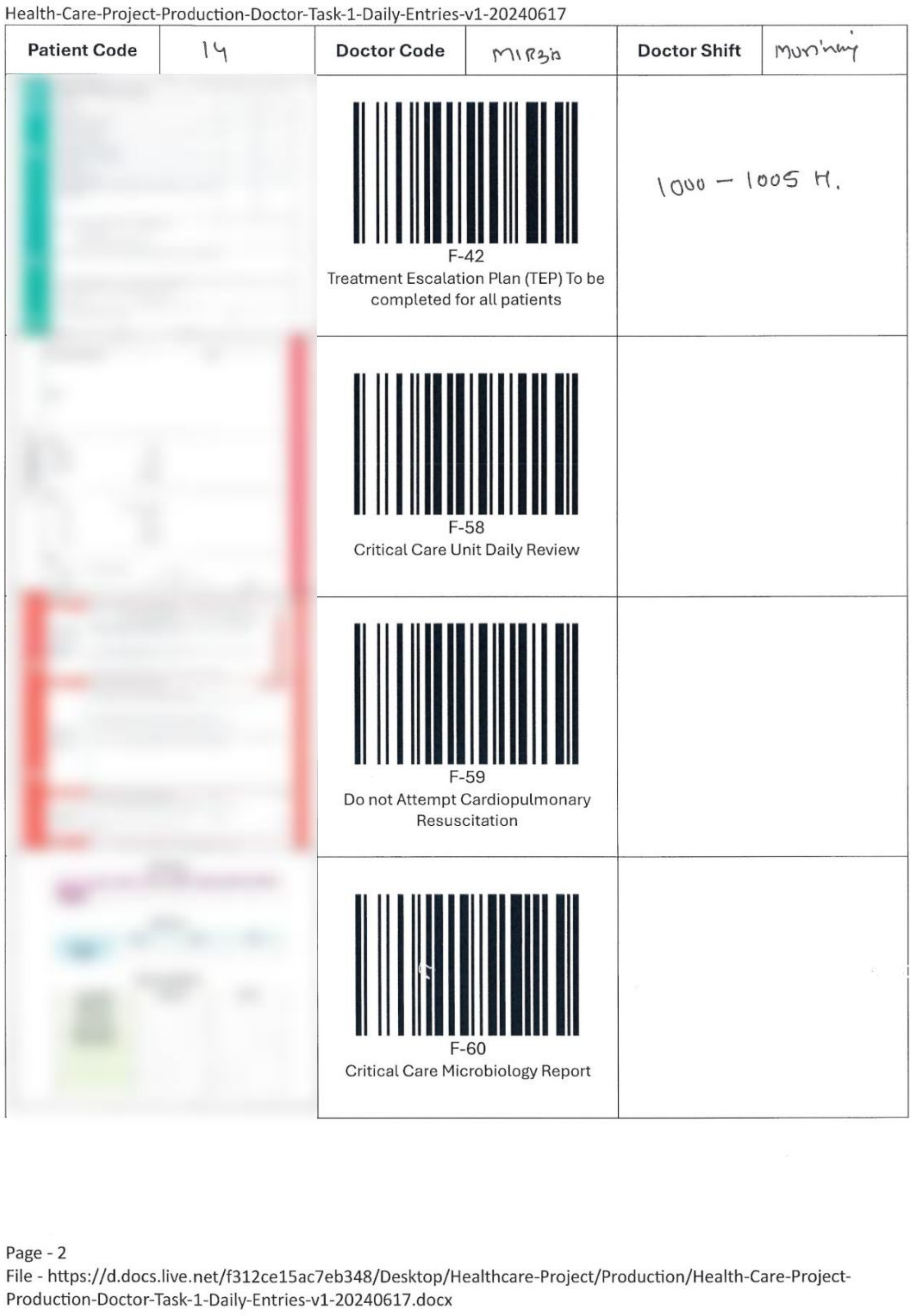

Health-Care-Project-Production-Doctor-Task-1-Daily-Entries-v1-20240617

Patient Code	14	Doctor Code	MIRza	Doctor Shift	Morning
		F-42 Treatment Escalation Plan (TEP) To be completed for all patients		1000 – 1005 H.	
		F-58 Critical Care Unit Daily Review			
		F-59 Do not Attempt Cardiopulmonary Resuscitation			
		F-60 Critical Care Microbiology Report			

Page - 2
File - https://d.docs.live.net/f312ce15ac7eb348/Desktop/Healthcare-Project/Production/Health-Care-Project-Production-Doctor-Task-1-Daily-Entries-v1-20240617.docx

Supplementary Figure 51: Result page – 51 for daily entries discrete event.

This daily entries discrete event was recorded using offline method by doctor staff. The demographic data was excluded in during the data collection.

The thumbnails of observation forms used in this figure were blurred for privacy reasons.

Supplementary Figure 52: Result Page 52

Health-Care-Project-Production-Doctor-Task-1-Daily-Entries-v2-20240617

Doctor		Daily Entries		Date	
Patient Code		Doctor Code	MIR2A	Doctor Shift	Morning.
		F-18 - WZW101 09/17<br>Clinical Notes			
		F-29 - NGV903 03/19<br>Consent Form 1 - Patient Agreement to Investigate or Treatment			
		F-31 - NGV1220 04/23<br>Critical Care Unit Prescription and Administration Record			
		F-34 - NGV1284 10/17<br>Critical Care Patient and Family Communication Record			

Page - 1

File - https://d.docs.live.net/f312ce15ac7eb348/Desktop/Healthcare-Project/Production/Health-Care-Project-Production-Doctor-Task-1-Daily-Entries-v2-20240617.docx

Supplementary Figure 52: Result page – 52 for daily entries discrete event.

No daily entries discrete events were recorded using offline method by doctor staff. The demographic data was excluded in during the data collection.

The thumbnails of observation forms used in this figure were blurred for privacy reasons.

Supplementary Figure 53: Result Page 53

Health-Care-Project-Production-Doctor-Task-1-Daily-Entries-v2-20240617

Patient Code		Doctor Code		Doctor Shift	Morning
		F-42 - NGV1550A 09/23<br>Treatment Escalation Plan (TEP) To be completed for all patients			
		F-58 - NGV1914 01/18<br>Critical Care Unit Daily Review		09:00 TO 0920H	
		F-59 - NGV2031 11/20<br>Do not Attempt Cardiopulmonary Resuscitation			
		F-60 - NGV2035 04/18<br>Critical Care Microbiology Report			

Page - 2
File - https://d.docs.live.net/f312ce15ac7eb348/Desktop/Healthcare-Project/Production/Health-Care-Project-Production-Doctor-Task-1-Daily-Entries-v2-20240617.docx

Supplementary Figure 53: Result page – 53 for daily entries discrete event.

This daily entries discrete event was recorded using offline method by doctor staff. The demographic data was excluded in during the data collection.

The thumbnails of observation forms used in this figure were blurred for privacy reasons.

Supplementary Figure 54: Result Page 54

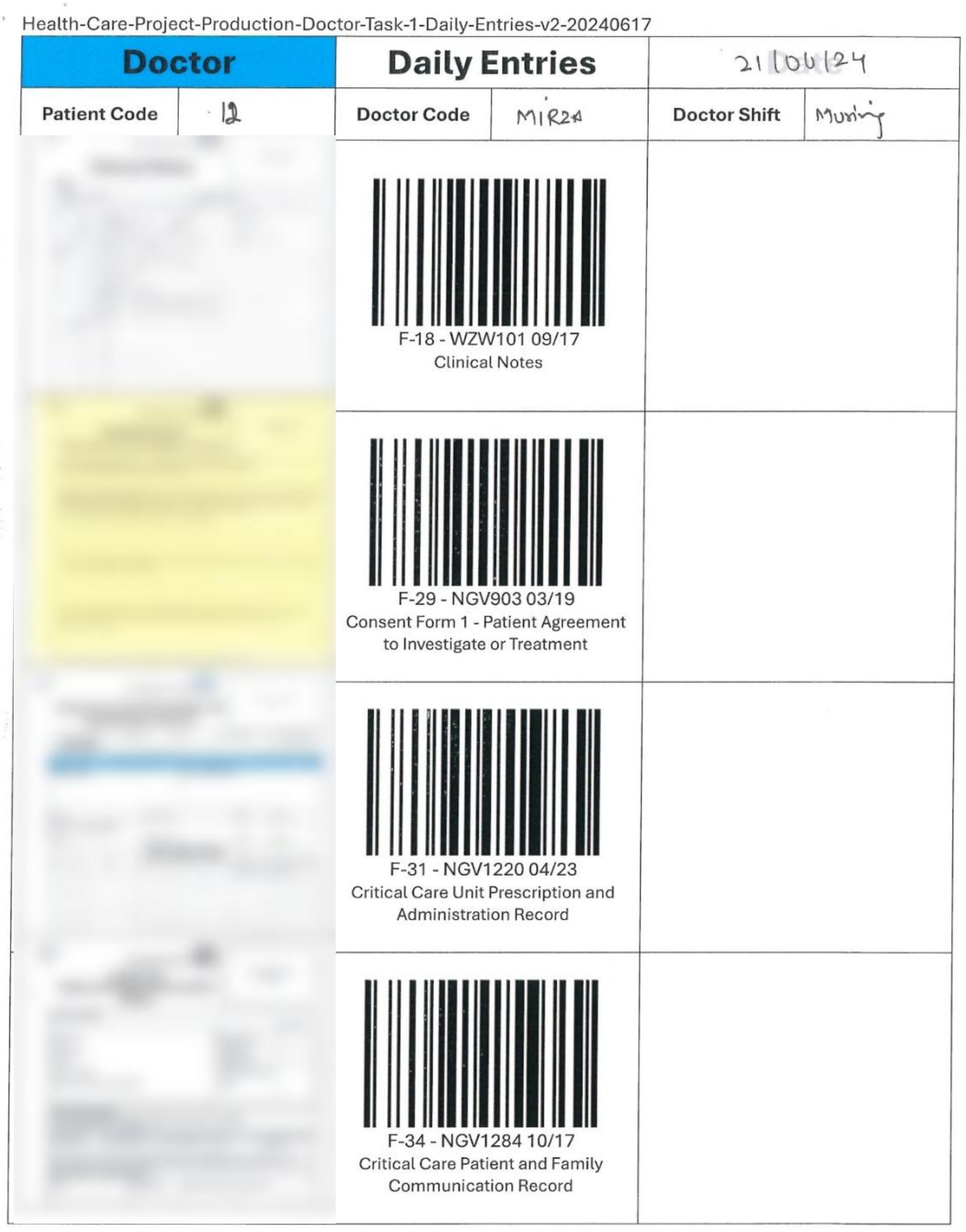

Health-Care-Project-Production-Doctor-Task-1-Daily-Entries-v2-20240617

Doctor		Daily Entries		21/06/24	
Patient Code	12	Doctor Code	MIR24	Doctor Shift	Morning
		F-18 - WZW101 09/17<br>Clinical Notes			
		F-29 - NGV903 03/19<br>Consent Form 1 - Patient Agreement to Investigate or Treatment			
		F-31 - NGV1220 04/23<br>Critical Care Unit Prescription and Administration Record			
		F-34 - NGV1284 10/17<br>Critical Care Patient and Family Communication Record			

Page - 1
File - https://d.docs.live.net/f312ce15ac7eb348/Desktop/Healthcare-Project/Production/Health-Care-Project-Production-Doctor-Task-1-Daily-Entries-v2-20240617.docx

Supplementary Figure 54: Result page – 54 for daily entries discrete event.

These daily entries discrete events were recorded using offline method by doctor staff. The demographic data was excluded in during the data collection.

The thumbnails of observation forms used in this figure were blurred for privacy reasons.

Supplementary Figure 55: Result Page 55

Health-Care-Project-Production-Doctor-Task-1-Daily-Entries-v2-20240617

Patient Code	12	Doctor Code	MIRZA	Doctor Shift	Morning
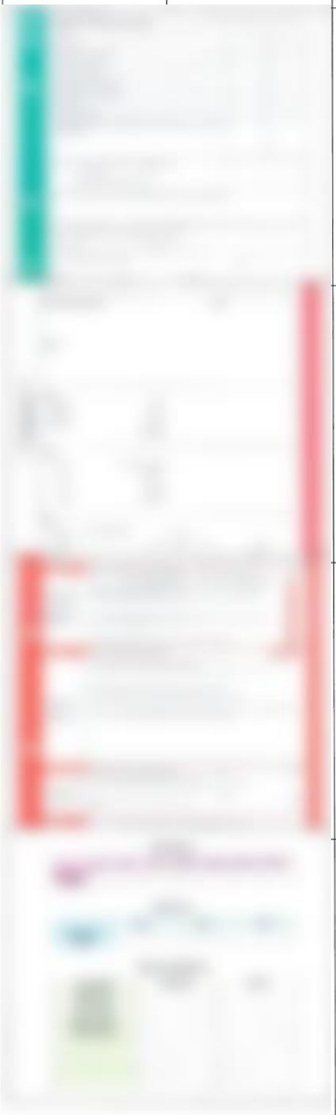		F-42 - NGV1550A 09/23 Treatment Escalation Plan (TEP) To be completed for all patients		[illegible] :	
		F-58 - NGV1914 01/18 Critical Care Unit Daily Review		0920 : 0935 -	
		F-59 - NGV2031 11/20 Do not Attempt Cardiopulmonary Resuscitation			
		F-60 - NGV2035 04/18 Critical Care Microbiology Report			

Page - 2
File - https://d.docs.live.net/f312ce15ac7eb348/Desktop/Healthcare-Project/Production/Health-Care-Project-Production-Doctor-Task-1-Daily-Entries-v2-20240617.docx

Supplementary Figure 55: Result page – 55 for daily entries discrete event.

This daily entries discrete event was recorded using offline method by doctor staff. The demographic data was excluded in during the data collection.

The thumbnails of observation forms used in this figure were blurred for privacy reasons.

Supplementary Figure 56: Result Page 56

Health-Care-Project-Production-Doctor-Task-1-Daily-Entries-v1-20240617

Doctor’s Daily Entries

Patient Code	13	Doctor Code	MIR3A	Doctor Shift	Morning
		F-18<br>Clinical Notes			
		F-29<br>Consent Form 1 - Patient Agreement to Investigate or Treatment			
		F-31<br>Critical Care Unit Prescription and Administration Record			
		F-34<br>Critical Care Patient and Family Communication Record			

Page - 1
File - https://d.docs.live.net/f312ce15ac7eb348/Desktop/Healthcare-Project/Production/Health-Care-Project-Production-Doctor-Task-1-Daily-Entries-v1-20240617.docx

Supplementary Figure 56: Result page – 56 for daily entries discrete event.

No daily entries discrete event were recorded using offline method by doctor staff. The demographic data was excluded in during the data collection.

The thumbnails of observation forms used in this figure were blurred for privacy reasons.

Supplementary Figure 57: Result Page 57

Health-Care-Project-Production-Doctor-Task-1-Daily-Entries-v1-20240617

Patient Code	13	Doctor Code	MIR30	Doctor Shift	Morning
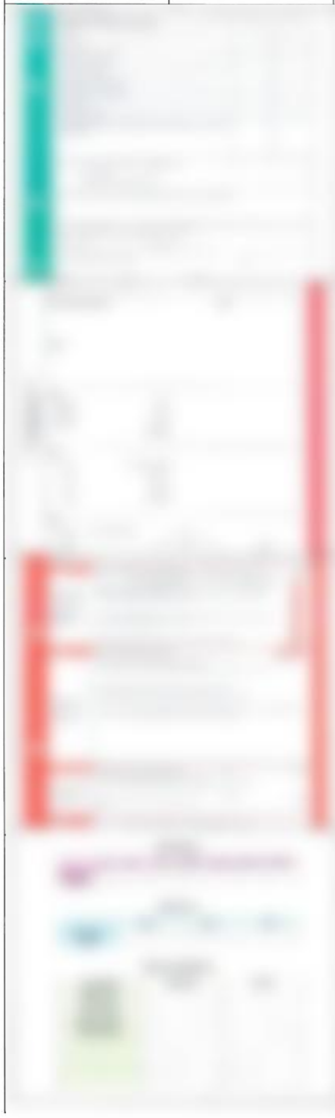		F-42<br>Treatment Escalation Plan (TEP) To be completed for all patients		11:00 TO 11:05	
		F-58<br>Critical Care Unit Daily Review			
		F-59<br>Do not Attempt Cardiopulmonary Resuscitation			
		F-60<br>Critical Care Microbiology Report			

Page - 2
File - https://d.docs.live.net/f312ce15ac7eb348/Desktop/Healthcare-Project/Production/Health-Care-Project-Production-Doctor-Task-1-Daily-Entries-v1-20240617.docx

Supplementary Figure 57: Result page – 57 for daily entries discrete event.

This daily entries discrete event was recorded using offline method by doctor staff. The demographic data was excluded in during the data collection.

The thumbnails of observation forms used in this figure were blurred for privacy reasons.

Supplementary Figure 58: Result Page 58

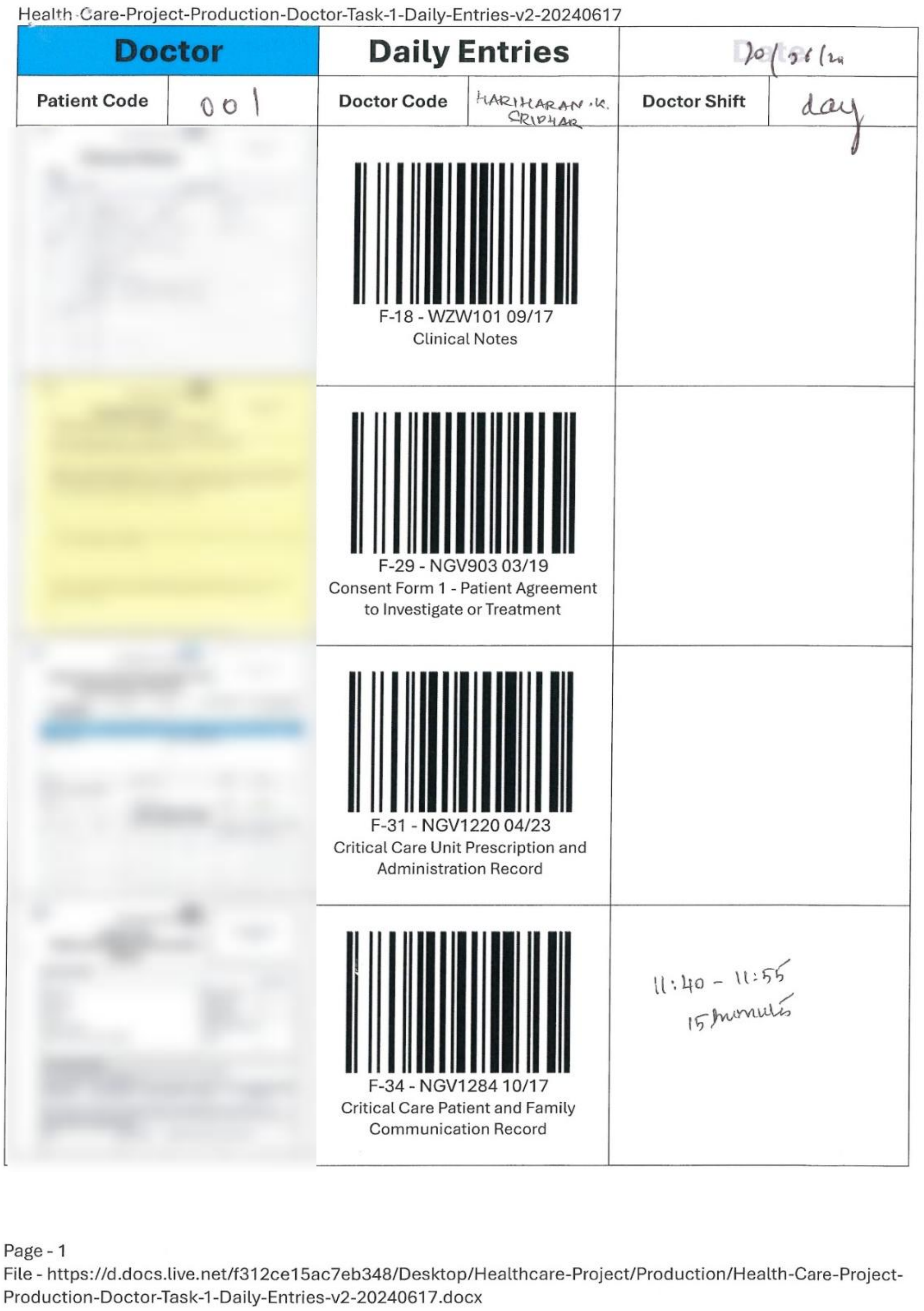

Health-Care-Project-Production-Doctor-Task-1-Daily-Entries-v2-20240617

Doctor		Daily Entries		Date 20/06/24	
Patient Code	001	Doctor Code	HARIHARAN.K. SRIDHAR	Doctor Shift	day

	F-18 - WZW101 09/17<br>Clinical Notes	
	F-29 - NGV903 03/19<br>Consent Form 1 - Patient Agreement to Investigate or Treatment	
	F-31 - NGV1220 04/23<br>Critical Care Unit Prescription and Administration Record	
	F-34 - NGV1284 10/17<br>Critical Care Patient and Family Communication Record	11:40 - 11:55<br>15 minutes

Page - 1
File - https://d.docs.live.net/f312ce15ac7eb348/Desktop/Healthcare-Project/Production/Health-Care-Project-Production-Doctor-Task-1-Daily-Entries-v2-20240617.docx

Supplementary Figure 58: Result page – 58 for daily entries discrete event.

This daily entries discrete event was recorded using offline method by doctor staff. The demographic data was excluded in during the data collection.

The thumbnails of observation forms used in this figure were blurred for privacy reasons.

Supplementary Figure 59: Result Page 59

Health-Care-Project-Production-Doctor-Task-1-Daily-Entries-v2-20240617

Patient Code		Doctor Code		Doctor Shift	
[illegible]		F-42 - NGV1550A 09/23<br>Treatment Escalation Plan (TEP) To be completed for all patients			
[illegible]		F-58 - NGV1914 01/18<br>Critical Care Unit Daily Review		08:33 to 08:56 Am<br>23 minutes	
[illegible]		F-59 - NGV2031 11/20<br>Do not Attempt Cardiopulmonary Resuscitation			
[illegible]		F-60 - NGV2035 04/18<br>Critical Care Microbiology Report			

Page - 2
File - https://d.docs.live.net/f312ce15ac7eb348/Desktop/Healthcare-Project/Production/Health-Care-Project-Production-Doctor-Task-1-Daily-Entries-v2-20240617.docx

Supplementary Figure 59: Result page – 59 for daily entries discrete event.

This daily entries discrete event was recorded using offline method by doctor staff. The demographic data was excluded in during the data collection.

The thumbnails of observation forms used in this figure were blurred for privacy reasons.

Supplementary Figure 60: Result Page 60

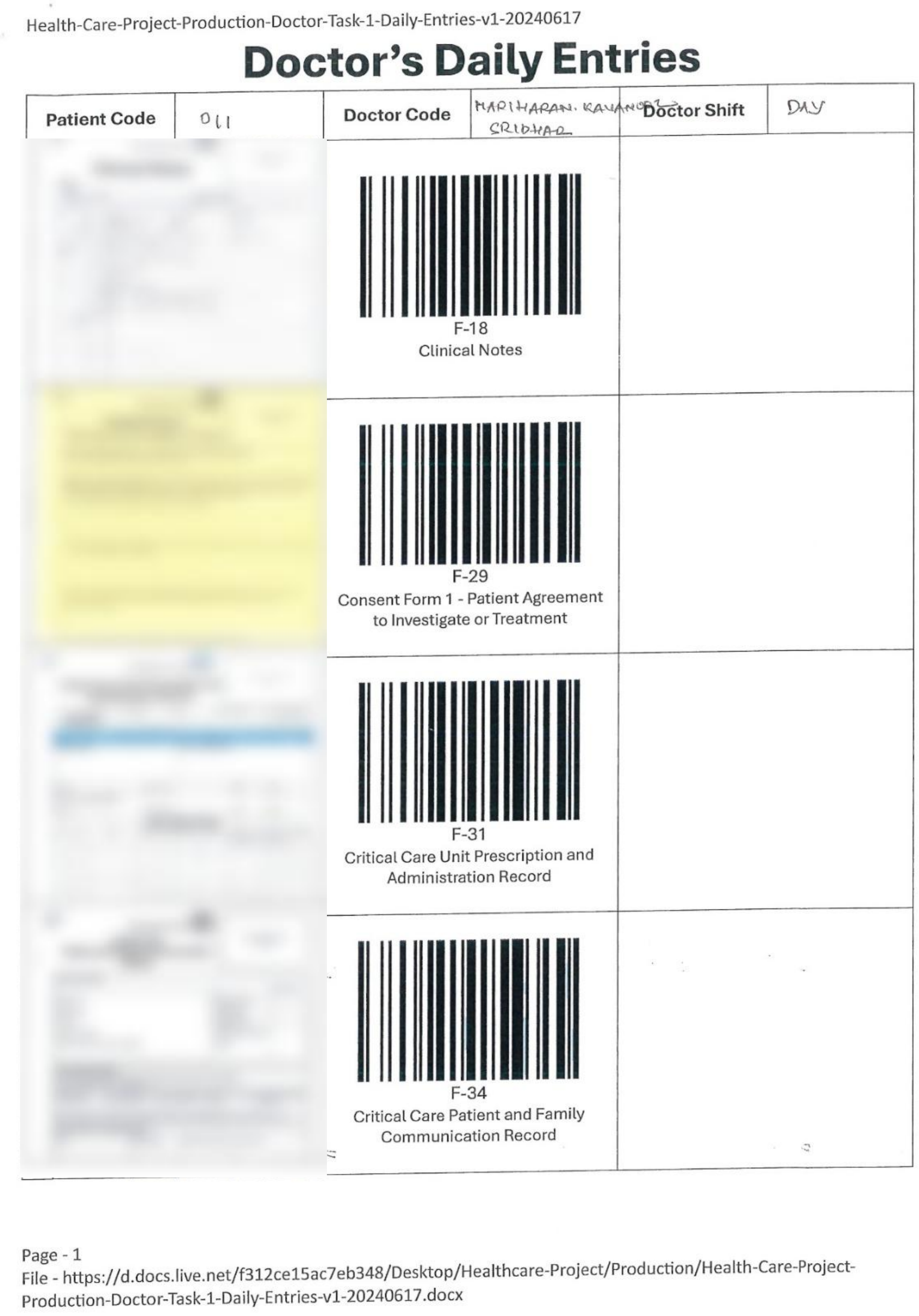

Health-Care-Project-Production-Doctor-Task-1-Daily-Entries-v1-20240617

Doctor's Daily Entries

Patient Code	011	Doctor Code	HARIHARAN. KAVANOOR SRIDHAR	Doctor Shift	DAY

	F-18 Clinical Notes	
	F-29 Consent Form 1 - Patient Agreement to Investigate or Treatment	
	F-31 Critical Care Unit Prescription and Administration Record	
	F-34 Critical Care Patient and Family Communication Record	

Page - 1
File - https://d.docs.live.net/f312ce15ac7eb348/Desktop/Healthcare-Project/Production/Health-Care-Project-Production-Doctor-Task-1-Daily-Entries-v1-20240617.docx

Supplementary Figure 60: Result page – 60 for daily entries discrete event.

No daily entries discrete events were recorded using offline method by doctor staff. The demographic data was excluded in during the data collection.

The thumbnails of observation forms used in this figure were blurred for privacy reasons.

Supplementary Figure 61: Result Page 61

Health-Care-Project-Production-Doctor-Task-1-Daily-Entries-v1-20240617

Patient Code		Doctor Code		Doctor Shift	
		F-42 Treatment Escalation Plan (TEP) To be completed for all patients			
		F-58 Critical Care Unit Daily Review		08: 30 - 08:40 Am Am 10 minutes	
		F-59 Do not Attempt Cardiopulmonary Resuscitation			
		F-60 Critical Care Microbiology Report			

Page - 2
File - https://d.docs.live.net/f312ce15ac7eb348/Desktop/Healthcare-Project/Production/Health-Care-Project-Production-Doctor-Task-1-Daily-Entries-v1-20240617.docx

Supplementary Figure 61: Result page – 61 for daily entries discrete event.

This daily entries discrete event was recorded using offline method by doctor staff. The demographic data was excluded in during the data collection.

The thumbnails of observation forms used in this figure were blurred for privacy reasons.

Supplementary Figure 62: Result Page 62

Health-Care-Project-Production-Doctor-Task-1-Daily-Entries-v1-20240617

Patient Code		Doctor Code		Doctor Shift	
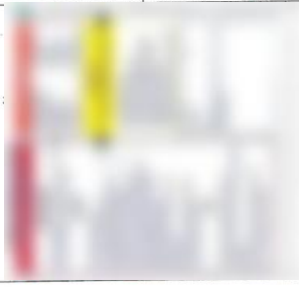		F-108<br>Procedure Checklist: Un-Proning			

Procedure - Intubation

15:00 - 15:15
Total - 15 minutes

Page - 6
File - https://d.docs.live.net/f312ce15ac7eb348/Desktop/Healthcare-Project/Production/Health-Care-Project-Production-Doctor-Task-1-Daily-Entries-v1-20240617.docx

Supplementary Figure 62: Result page – 62 for daily entries discrete event.

This daily entries discrete event was recorded using offline method by doctor staff. The demographic data was excluded in during the data collection.

The thumbnails of observation forms used in this figure were blurred for privacy reasons.

Supplementary Figure 63: Result Page 63

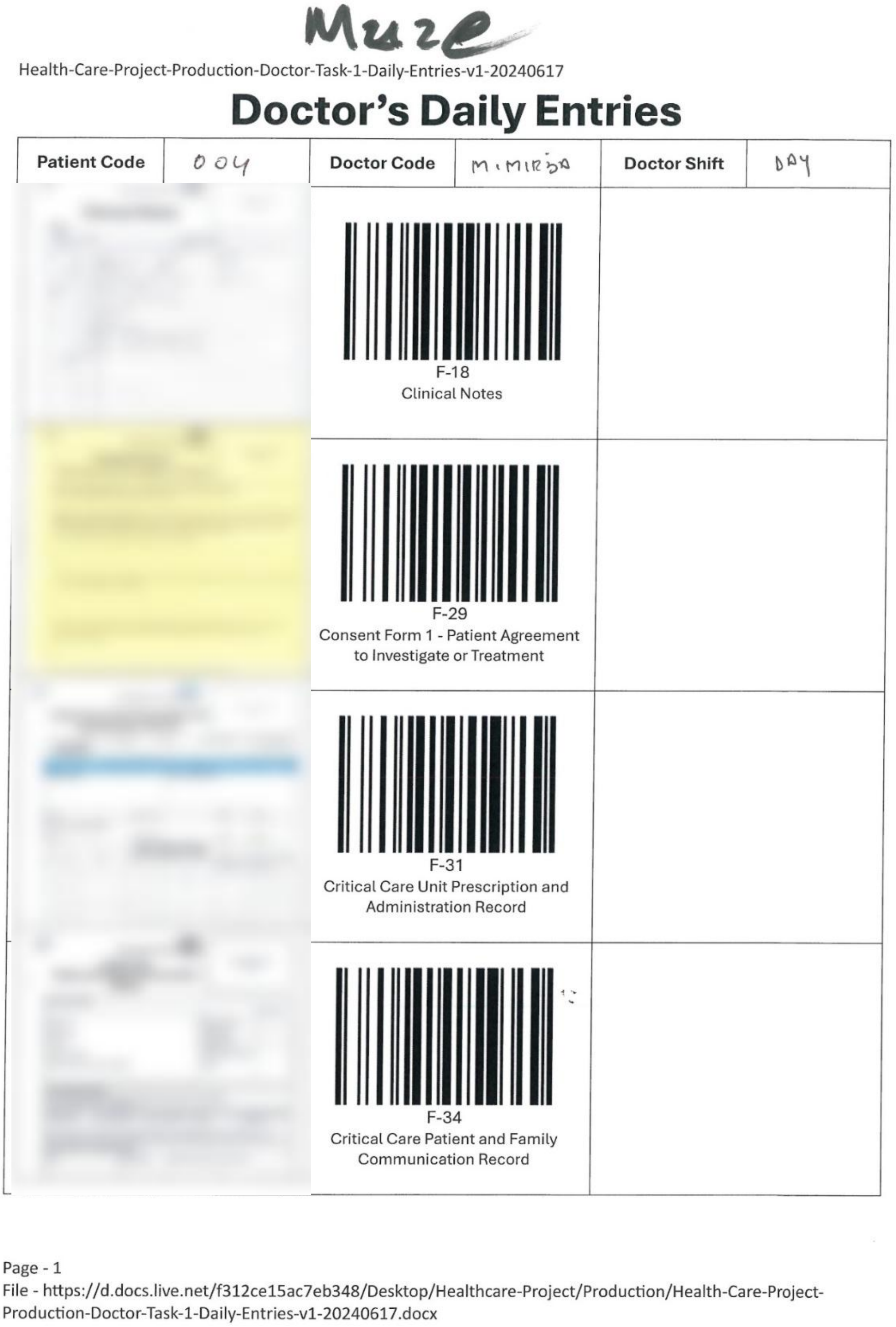

Muze

Health-Care-Project-Production-Doctor-Task-1-Daily-Entries-v1-20240617

Doctor's Daily Entries

Patient Code	004	Doctor Code	M.MIRZA	Doctor Shift	DAY
		F-18 Clinical Notes			
		F-29 Consent Form 1 - Patient Agreement to Investigate or Treatment			
		F-31 Critical Care Unit Prescription and Administration Record			
		F-34 Critical Care Patient and Family Communication Record			

Page - 1

File - https://d.docs.live.net/f312ce15ac7eb348/Desktop/Healthcare-Project/Production/Health-Care-Project-Production-Doctor-Task-1-Daily-Entries-v1-20240617.docx

Supplementary Figure 63: Result page – 63 for daily entries discrete event.

No daily entries discrete events were recorded using offline method by doctor staff. The demographic data was excluded in during the data collection.

The thumbnails of observation forms used in this figure were blurred for privacy reasons.

Supplementary Figure 64: Result Page 64

Health-Care-Project-Production-Doctor-Task-1-Daily-Entries-v1-20240617

Patient Code		Doctor Code		Doctor Shift	
		F-42 Treatment Escalation Plan (TEP) To be completed for all patients			
		F-58 Critical Care Unit Daily Review		0830 - 0900H	
		F-59 Do not Attempt Cardiopulmonary Resuscitation			
		F-60 Critical Care Microbiology Report			

Page - 2
File - https://d.docs.live.net/f312ce15ac7eb348/Desktop/Healthcare-Project/Production/Health-Care-Project-Production-Doctor-Task-1-Daily-Entries-v1-20240617.docx

Supplementary Figure 64: Result page – 64 for daily entries discrete event.

This daily entries discrete event was recorded using offline method by doctor staff. The demographic data was excluded in during the data collection.

The thumbnails of observation forms used in this figure were blurred for privacy reasons.

Supplementary Figure 65: Result Page 65

Health-Care-Project-Production-Doctor-Task-1-Daily-Entries-v1-20240617

Patient Code		Doctor Code	Fyroz mohamed	Doctor Shift	Night
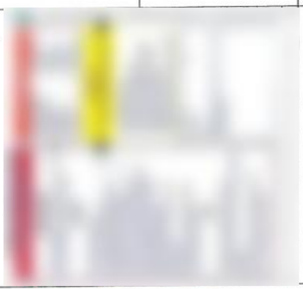		F-108<br>Procedure Checklist: Un-Proning			

Blood prescription

03.29 ~ [illegible] 03.31

Page - 6
File - https://d.docs.live.net/f312ce15ac7eb348/Desktop/Healthcare-Project/Production/Health-Care-Project-Production-Doctor-Task-1-Daily-Entries-v1-20240617.docx

Supplementary Figure 65: Result page – 65 for daily entries discrete event.

This daily entries discrete event was recorded using offline method by doctor staff. The demographic data was excluded in during the data collection.

The thumbnails of observation forms used in this figure were blurred for privacy reasons.

Supplementary Figure 66: Result Page 66

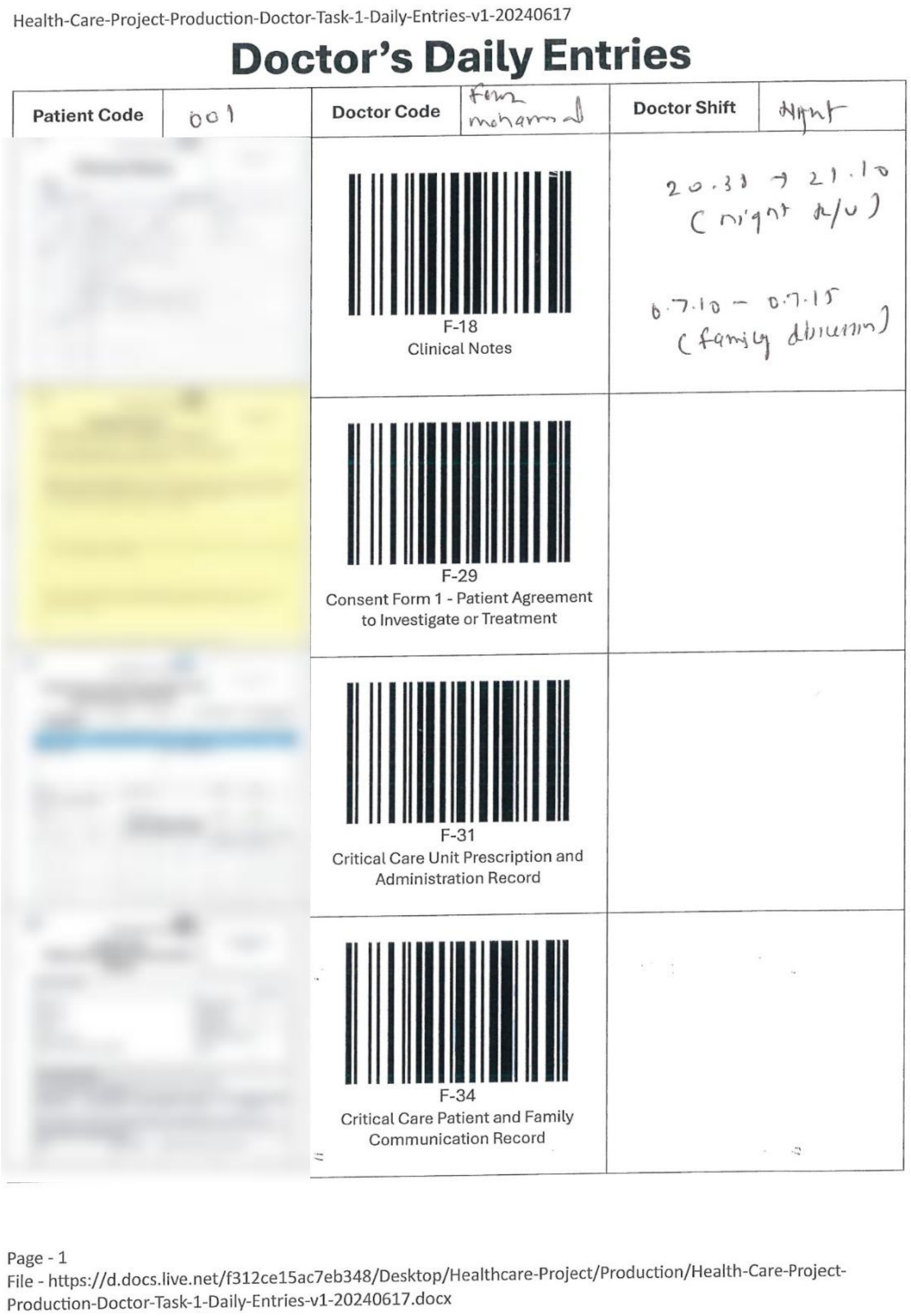
Health-Care-Project-Production-Doctor-Task-1-Daily-Entries-v1-20240617

Doctor's Daily Entries

Patient Code	001	Doctor Code	Form mohammad	Doctor Shift	Night
		F-18 Clinical Notes		20.30 → 21.10 (night f/u) 07.10 – 07.15 (family discussion)	
		F-29 Consent Form 1 - Patient Agreement to Investigate or Treatment			
		F-31 Critical Care Unit Prescription and Administration Record			
		F-34 Critical Care Patient and Family Communication Record			

Page - 1
File - https://d.docs.live.net/f312ce15ac7eb348/Desktop/Healthcare-Project/Production/Health-Care-Project-Production-Doctor-Task-1-Daily-Entries-v1-20240617.docx

Supplementary Figure 66: Result page – 66 for daily entries discrete event.

These daily entries discrete events were recorded using offline method by doctor staff. The demographic data was excluded in during the data collection.

The thumbnails of observation forms used in this figure were blurred for privacy reasons.

Supplementary Figure 67: Result Page 67

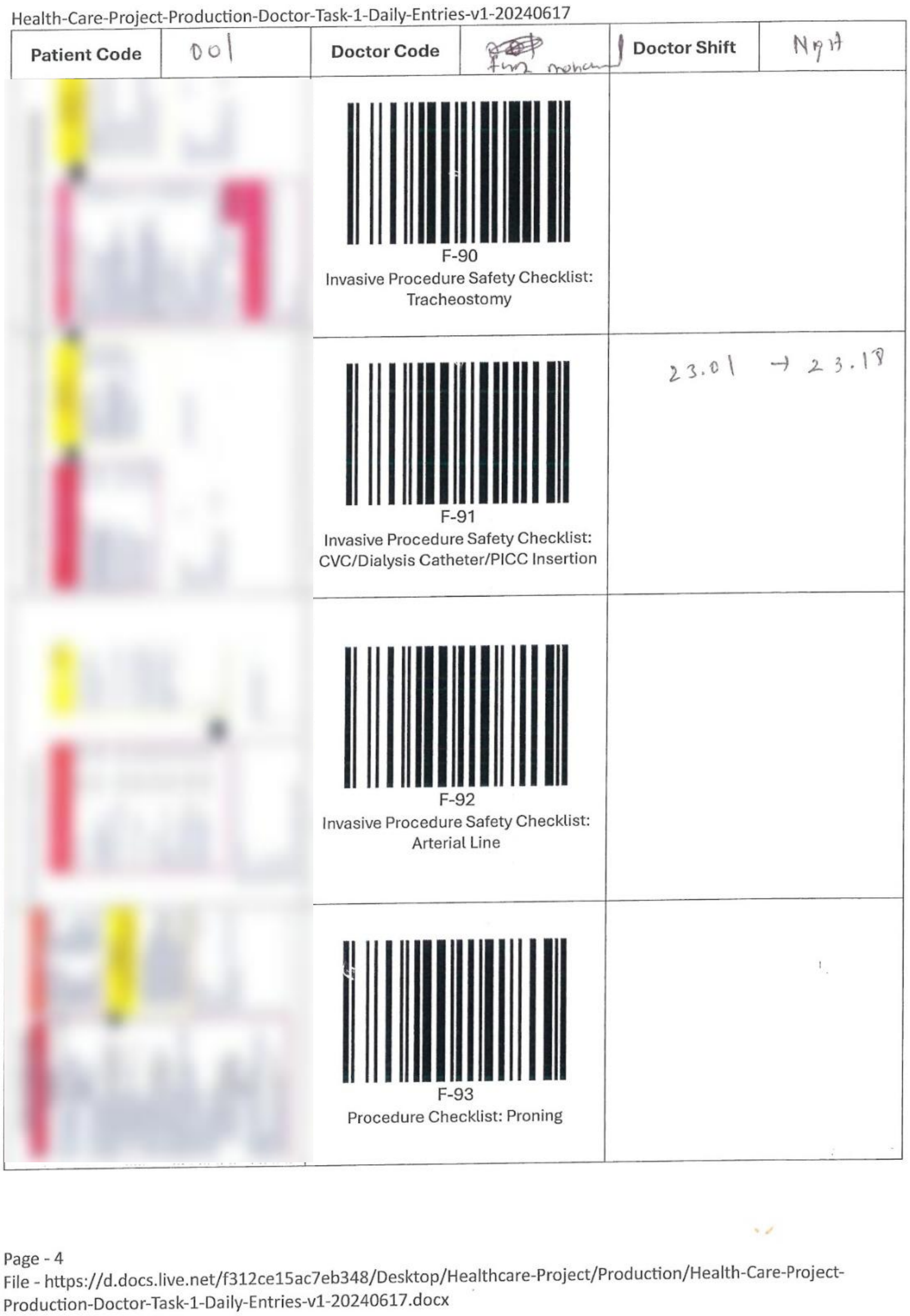

Health-Care-Project-Production-Doctor-Task-1-Daily-Entries-v1-20240617

Patient Code	001	Doctor Code	[illegible]	Doctor Shift	Night
		F-90 Invasive Procedure Safety Checklist: Tracheostomy			
		F-91 Invasive Procedure Safety Checklist: CVC/Dialysis Catheter/PICC Insertion		23.01 → 23.18	
		F-92 Invasive Procedure Safety Checklist: Arterial Line			
		F-93 Procedure Checklist: Proning			

Page - 4
File - https://d.docs.live.net/f312ce15ac7eb348/Desktop/Healthcare-Project/Production/Health-Care-Project-Production-Doctor-Task-1-Daily-Entries-v1-20240617.docx

Supplementary Figure 67: Result page – 67 for daily entries discrete event.

This daily entries discrete event was recorded using offline method by doctor staff. The demographic data was excluded in during the data collection.

The thumbnails of observation forms used in this figure were blurred for privacy reasons.

Supplementary Figure 68: Result Page 68

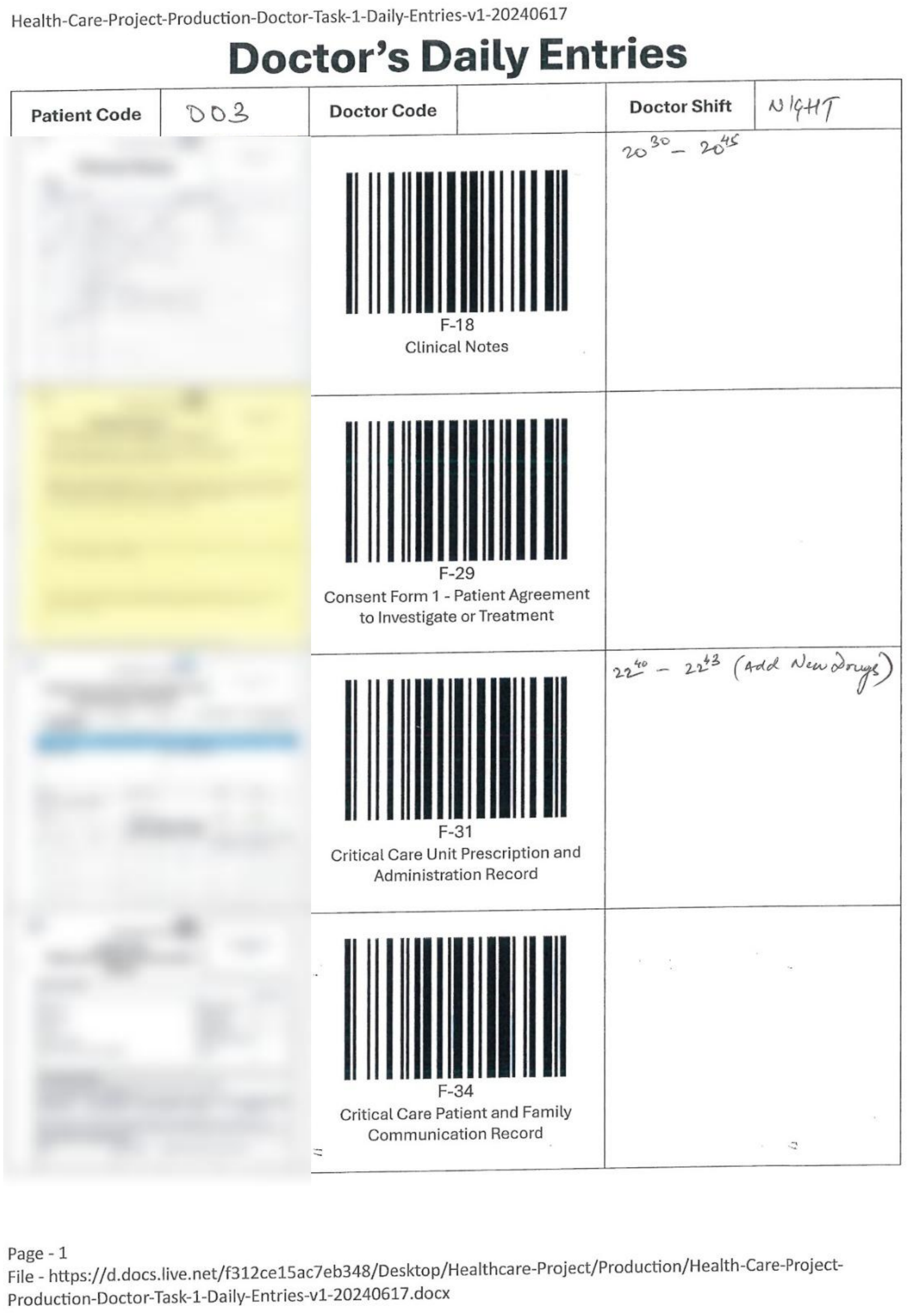

Health-Care-Project-Production-Doctor-Task-1-Daily-Entries-v1-20240617

Doctor's Daily Entries

Patient Code	D03	Doctor Code		Doctor Shift	NIGHT
		F-18 Clinical Notes		$20^{30} - 20^{45}$	
		F-29 Consent Form 1 - Patient Agreement to Investigate or Treatment			
		F-31 Critical Care Unit Prescription and Administration Record		$22^{40} - 22^{43}$ (Add New Drugs)	
		F-34 Critical Care Patient and Family Communication Record			

Page - 1
File - https://d.docs.live.net/f312ce15ac7eb348/Desktop/Healthcare-Project/Production/Health-Care-Project-Production-Doctor-Task-1-Daily-Entries-v1-20240617.docx

Supplementary Figure 68: Result page – 68 for daily entries discrete events.

These daily entries discrete events were recorded using offline method by doctor staff. The demographic data was excluded in during the data collection.

The thumbnails of observation forms used in this figure were blurred for privacy reasons.

Supplementary Figure 69: Test Schedule

Distance - 10 Meters
BCR

Staff	Patient	Task	Activity	Start Time	End Time
S-1	P-2	F-1	A1	10:30:00	10:35:00
S-1	P-2	F-2	A2	10:40:00	10:50:00
S-1	P-2	F-3	A3	10:50:00	11:15:00
S-1	P-2	F-4	A4	11:15:00	11:30:00
S-1	P-2	F-5	A5	12:00:00	12:45:00
S-1	P-1	F-6	A6	12:45:00	13:00:00
S-1	P-1	F-7	A7	13:00:00	14:00:00
S-1	P-1	F-8	A8	14:00:00	15:00:00
S-1	P-1	F-9	A9	15:00:00	15:45:00
S-1	P-1	F-10	A10	15:45:00	16:00:00

Distance - Nearby
BCR

Staff	Patient	Task	Activity	Start Time	End Time
S-3	P-3	F-1	A11	10:35:00	10:40:00
S-3	P-3	F-2	A12	10:45:00	10:55:00
S-3	P-3	F-3	A13	10:55:00	11:20:00
S-3	P-3	F-4	A14	11:20:00	11:35:00
S-3	P-3	F-5	A15	12:05:00	12:50:00
S-3	P-3	F-6	A16	12:50:00	13:05:00
S-3	P-3	F-7	A17	13:05:00	14:05:00
S-3	P-3	F-8	A18	14:05:00	15:05:00
S-3	P-3	F-9	A19	15:05:00	15:50:00
S-3	P-3	F-10	A20	15:50:00	16:00:00

Distance - 10 Meters
BCR

Staff	Patient	Task	Activity	Start Time	End Time
S-2	P-1	F-1	A11	10:35:00	10:40:00
S-2	P-1	F-2	A12	10:45:00	10:55:00
S-2	P-1	F-3	A13	10:55:00	11:20:00
S-2	P-1	F-4	A14	11:20:00	11:35:00
S-2	P-1	F-5	A15	12:05:00	12:50:00
S-2	P-2	F-6	A16	12:50:00	13:05:00
S-2	P-2	F-7	A17	13:05:00	14:05:00
S-2	P-2	F-8	A18	14:05:00	15:05:00
S-2	P-2	F-9	A19	15:05:00	15:50:00
S-2	P-2	F-10	A20	15:50:00	16:00:00

Online
Remote

Staff	Patient	Task	Activity	Start Time	End Time
S-4	P-1	F-1	A11	10:40:00	10:45:00
S-4	P-1	F-2	A12	10:50:00	11:00:00
S-4	P-1	F-3	A13	11:00:00	11:25:00
S-4	P-1	F-4	A14	11:25:00	11:40:00
S-4	P-1	F-5	A15	12:10:00	12:55:00
S-4	P-2	F-6	A16	12:55:00	13:10:00
S-4	P-2	F-7	A17	13:10:00	14:10:00
S-4	P-2	F-8	A18	14:10:00	15:10:00
S-4	P-2	F-9	A19	15:10:00	15:55:00
S-4	P-2	F-10	A20	15:55:00	16:00:00

Online
Remote

Staff	Patient	Task	Activity	Start Time	End Time
S-5	S-4	F-1	A11	00:05:00	00:05:00
S-5	S-4	F-2	A12	00:05:00	00:05:00
S-5	S-4	F-3	A13	00:05:00	00:05:00
S-5	S-4	F-4	A14	00:05:00	00:05:00
S-5	S-4	F-5	A15	00:05:00	00:05:00
S-5	P-3	F-6	A16	00:05:00	00:05:00
S-5	P-3	F-7	A17	00:05:00	00:05:00
S-5	P-3	F-8	A18	00:05:00	00:05:00
S-5	P-3	F-9	A19	00:05:00	00:05:00
S-5	P-3	F-10	A20	00:05:00	00:00:00

1- [illegible]
2- Rukhsar
3- Wasan
4- Asad
5- Saeed

Supplementary Figure 69: Test schedule.

This figure shows schedules which were designed during the testing phase. During this phase this study selected test subjects (n=5) and received responses (n=3) of comments on user interface and usability. Based on responses this study improved the interfaces for non-technical users to navigate through digital twins.

Supplementary Figure 70: Test Result Page 1

S-20

Distance - 10 Meters

BCR

Staff	Patient	Task	Activity	Start Time	End Time	Time Elapsed	Method	Check
S-1	P-2	F-1	A1	13:00:00	13:05:00	5 Mins	Online	✓ [illegible]
S-1	P-2	F-2	A2	13:05:00	13:30:00	25 Mins	Online	✓ [illegible]
S-1	P-2	F-3	A3	13:30:00	13:40:00	10 Mins	Online	✓ [illegible]
S-1	P-2	F-4	A4	13:40:00	13:45:00	5 Mins	Online	✓ [illegible]
S-1	P-2	F-5	A5	13:45:00	14:00:00	15 Mins	Online	✓ [illegible] ②
S-1	P-1	F-6	A6	14:00:00	14:15:00	15 Mins	Offline	✓ [illegible]
S-1	P-1	F-7	A7	14:15:00	~~14:25:00~~	10 Mins	Offline	✓
S-1	P-1	F-8	A8	14:25:00	~~14:30:00~~	5 Mins	Offline	✓ [illegible]
S-1	P-1	F-9	A9	~~14:30:00~~	~~14:45:00~~	15 Mins	Offline	[illegible]
S-1	P-1	F-10	A10	14:45:00	~~15:00:00~~	15 Mins	Offline	[illegible]

Margin notes: S-14.24; -E-14.51; -E-14.30; 14.32; 14.33; E-1449; same shart tian

* Online Submit

– the Submit button takes too long to Submit, forcing the user to click Several times (PC could use Status loading ...)

A2 – F-2 hours/min don't allign with input: Start: 13:48 × end 13:30 ✓

A3 – F-3 " " " start: 12:56 × end 13:40

---

* Offline

A7 – Had to refresh the Dashboard several times to get result - "Complete"

– Dashboard is not very clear

* the Date/time is Correct in the barcode Scanner approach :)

A8 – Note – Scanned F-8 at 14:30, as the first scan was not recorded. (Maybe I didn't Scan Correctly)

A8–A9 [To Complete F8 & F9 I had to Scan twice, Could be to user experience issue.

A10 : Scanned but didn't Show on System

Supplementary Figure 70: Test Result Page 1.

This figure shows responses from test subject - 1.

Supplementary Figure 71: Test Result Page 2

Distance - 10 Meters

BCR

Staff	Patient	Task	Activity	Start Time	End Time	Time Elapsed	Method	Check
S-2	P-2	F-1	A1	13:45:00	13:50:00	5 Mins	Offline	✓
S-2	P-2	F-2	A2	13:50:00	14:00:00	10 Mins	Offline	✓
S-2	P-2	F-3	A3	14:00:00	14:05:00	5 Mins	Offline	✓
S-2	P-2	F-4	A4	14:05:00	14:10:00	5 Mins	Offline	✓
S-2	P-2	F-5	A5	14:05:00	14:15:00	10 Mins	Offline	✓
S-2	P-1	F-6	A6	14:15:00	14:25:00	10 Mins	Online	✓
S-2	P-1	F-7	A7	14:05:00	14:25:00	20 Mins	Online	✓
S-2	P-1	F-8	A8	14:25:00	14:30:00	5 Mins	Online	✓
S-2	P-1	F-9	A9	14:30:00	14:50:00	20 Mins	Online	✓
S-2	P-1	F-10	A10	14:45:00	15:00:00	15 Mins	Online	✓

Supplementary Figure 71: Test Result Page 2.

This figure shows responses from test subject - 2.

Supplementary Figure 72: Test Result Page 3

S-22

Online
Remote

Staff	Patient	Task	Activity	Start Time	End Time	Time Elapsed	Method	Check
S-3	P-1	F-1	A1	13:45:00	13:55:00	10 Mins	Online	✓
S-3	P-1	F-2	A2	13:50:00	14:05:00	15 Mins	Online	✓
S-3	P-1	F-3	A3	14:00:00	14:05:00	5 Mins	Online	✓
S-3	P-1	F-4	A4	14:07:00	14:10:00	3 Mins	Online	✓
S-3	P-1	F-5	A5	14:10:00	14:18:00	8 Mins	Online	✓
S-3	P-2	F-6	A6	14:15:00	14:26:00	11 Mins	Online	✓
S-3	P-2	F-7	A7	14:28:00	14:35:00	7 Mins	Online	✓
S-3	P-2	F-8	A8	14:30:00	14:35:00	5 Mins	Online	✓
S-3	P-2	F-9	A9	14:30:00	14:55:00	25 Mins	Online	✓
S-3	P-2	F-10	A10	14:45:00	15:00:00	15 Mins	Online	✓

Supplementary Figure 72: Test Result Page 3.

This figure shows responses from test subject - 3.
